# Discovery of 1,3-disubstituted prop-2-en-1-one derivatives as inhibitors of neutrophilic inflammation via modulation of MAPK and Akt pathways

**DOI:** 10.1080/14756366.2024.2402988

**Published:** 2024-09-19

**Authors:** Mohammad Abdel-Halim, Dalia S. El-Gamil, Mennatallah A. Hammam, Mohamed El-Shazly, Yi-Hsuan Wang, Po-Hsiung Kung, Yu-Cheng Chen, Michal Korinek, Ashraf H. Abadi, Matthias Engel, Tsong-Long Hwang

**Affiliations:** a Department of Pharmaceutical Chemistry, Faculty of Pharmacy and Biotechnology, German University in Cairo, Cairo, Egypt; b Department of Chemistry, Faculty of Pharmacy, Ahram Canadian University, Cairo, Egypt; c School of Life and Medical Sciences, University of Hertfordshire Hosted by Global Academic Foundation, Cairo, Egypt; d Department of Pharmacognosy, Faculty of Pharmacy, Ain-Shams University, Cairo, Egypt; e Graduate Institute of Natural Products, College of Medicine, Chang Gung University, Taoyuan, Taiwan; f Graduate Institute of Healthy Industry Technology, Research Center for Chinese Herbal Medicine, College of Human Ecology, Chang Gung University of Science and Technology, Taoyuan, Taiwan; g Graduate Institute of Natural Products, College of Pharmacy, Kaohsiung Medical University, Kaohsiung, Taiwan; h Pharmaceutical and Medicinal Chemistry, Saarland University, Saarbrücken, Germany; i Department of Anesthesiology, Chang Gung Memorial Hospital, Taoyuan, Taiwan; j Department of Chemical Engineering, Ming Chi University of Technology, New Taipei City, Taiwan

**Keywords:** Enone derivatives, superoxide, elastase, human neutrophils, inflammation

## Abstract

Targeting neutrophil function has gained attention as a propitious therapeutic strategy for diverse inflammatory diseases. Accordingly, a series of enone-based derivatives were developed to inhibit neutrophil-mediated inflammation, showing promise for treating inflammatory diseases. These compounds fall into two clusters with distinct effects: one inhibits neutrophilic superoxide (SO) anion production and elastase release triggered by N-formyl-Met-Leu-Phe (fMLF), with compound **6a** being most effective (IC_50_ values of 1.23 and 1.37 μM, respectively), affecting c-Jun N-terminal kinase (JNK) and Akt phosphorylation. The second cluster suppresses formation of SO anion without affecting elastase levels, surpassed by compound **26a** (IC_50_ of 1.56 μM), which attenuates various mitogen-activated protein kinases (MAPKs) with minimal Akt impact. Notably, none of the tested compounds showed cytotoxicity in human neutrophils, underscoring their potential as therapeutic agents against inflammatory diseases.

## Graphical Abstract

**Figure:**
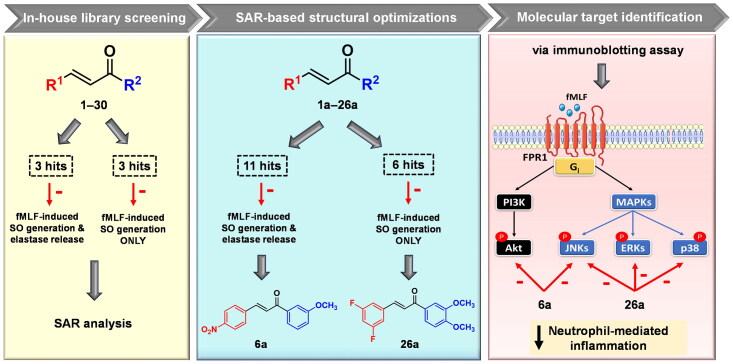

## Introduction

Inflammation is a cornerstone of the immune system, protecting hosts from harmful stimuli and pathogens. Tightly regulated inflammatory responses also contribute to stress adaptation, homeostasis restoration, tissue repair, and regeneration^1^^,^^2^. On the contrary, dysregulated inflammatory pathways have been correlated to a plethora of chronic and degenerative diseases such as cancer, atherosclerosis, diabetes mellitus, disease, rheumatoid arthritis, non-alcoholic fatty liver, and autoimmune disorders^1^^,^^3^.

Since neutrophils are the most numerous circulating leukocytes, they act as the initial line of host defence where mounting levels of chemoattractants direct them to infection sites^4^. Neutrophils detect, capture, and eliminate invading pathogens through a series of events such as phagocytosis, degranulation, and the release of neutrophil extracellular traps (NETs), which are partially fuelled by the generation of reactive oxygen species (ROS)^4^^,^^5^. Moreover, neutrophils are inflammation modulators, which, through the production of immunomodulatory cytokines, can recruit and govern the activities of other inflammatory cells, such as macrophages^6^^,^^7^. Oxidative burst is a crucial component of the neutrophil-mediated inflammatory and anti-pathogenic pathways. When stimulated, neutrophils stimulate their nicotinamide adenine dinucleotide phosphate (NADPH) oxidase complex, which produces large quantities of superoxide (SO) anions. Sequentially, precursors of free radicals, hydrogen peroxide and hypochlorous acid, are produced by neutrophils’ SO dismutase and myeloperoxidase enzymes, respectively^8^^,^^9^.

**Figure 1.**
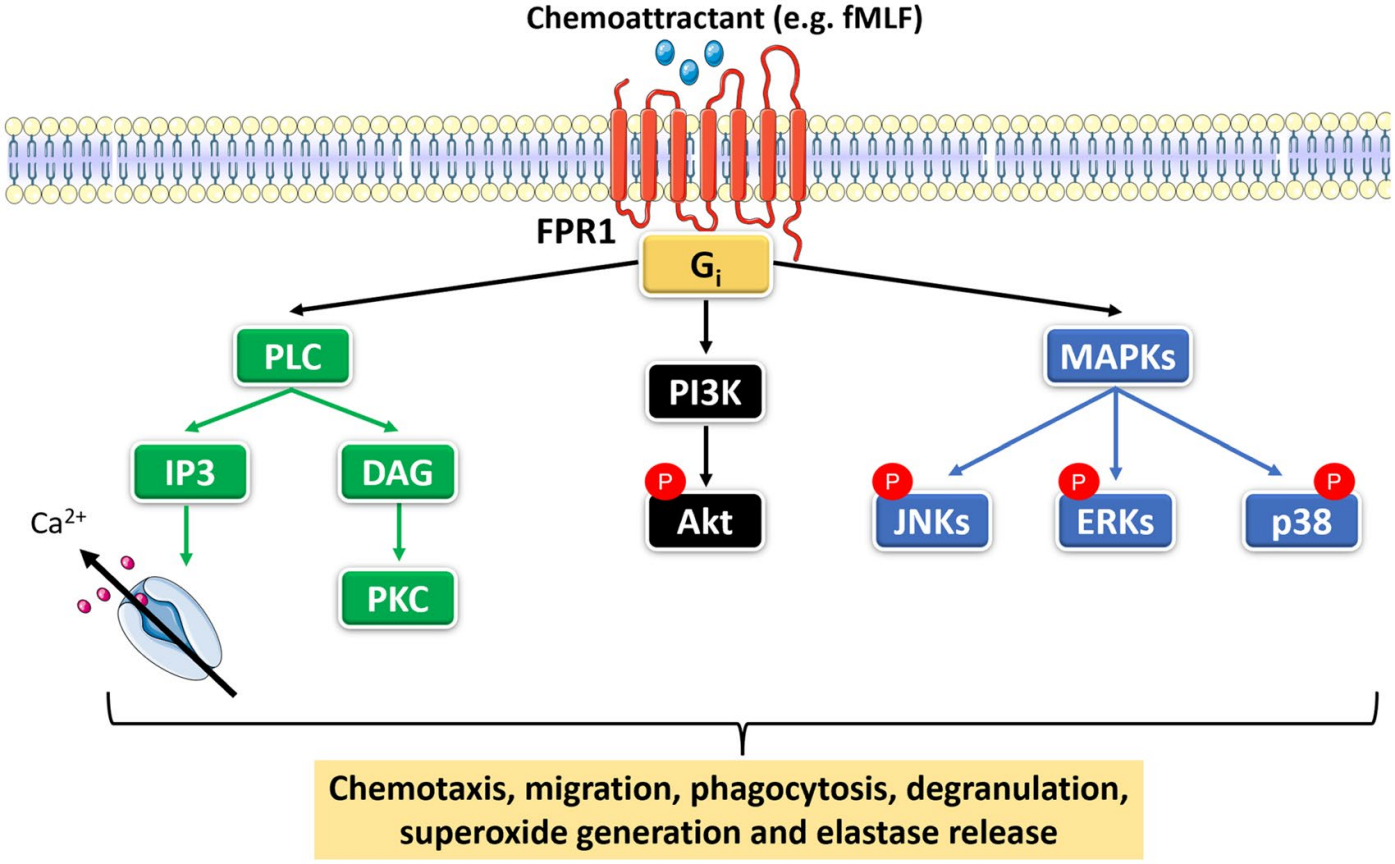
fMLF-induced signalling cascade for neutrophil activation. Chemoattractants such as formyl-l-methionyl-l-leucyl-l-phenylalanine (fMLF), are “formyl peptide receptor 1” (FPR1) agonists that orchestrate various neutrophil-activating signalling cascades. The activation of phosphoinositide-specific phospholipase C (PLC) leads to the production of inositol triphosphate (IP3) and diacylglycerol (DAG), which in turn triggers the activation of protein kinase C (PKC) and the release of calcium. Moreover, FPR activation modulates mitogen-activated protein kinases (MAPKs) and phosphoinositide 3-kinase (PI3K)/Akt signalling axes which play pivotal roles in elastase release, chemotaxis, NADPH oxidase activation, and superoxide anion generation (Self-created image).

Chemotactic *N*-formylated peptides such as formyl-l-methionyl-l-leucyl-l-phenylalanine (fMLF), are potent agonists of the G protein-coupled receptors (GPCRs) (the high-affinity “formyl peptide receptor 1” FPR1 and its low-affinity variant FPR2) that govern various signalling cascades promoting phagocytosis, degranulation, cell migration, and proinflammatory mediators generation (Figure 1)^10^. Phospholipase C (PLC) activation generates inositol triphosphate (IP3) that prompts intracellular Ca^2+^ mobilisation, along with diacylglycerol (DAG) that activates distinct isoforms of protein kinase C (PKC)^11^^,^^12^. Furthermore, FPR1 activation triggers phosphorylation of neutrophilic mitogen-activated protein kinases (MAPKs), namely extracellular signal-regulated kinases (ERKs), cJNK, and the p38 MAPK, that promote ROS generation and trigger chemotaxis via the regulation of surface receptor expression^2^^,^^13–15^. In addition, the phosphoinositide-3-kinase (PI3K)/Akt signalling axis can modulate neutrophil-mediated chemotaxis, NADPH oxidase activation, degranulation, elastase release, and SO anion production^16–18^.

Discrepancies in activation, clearance, or recruitment levels of neutrophils during the abovementioned processes can cause exaggerated inflammatory responses, neutrophil degranulation, and excessive tissue injury^7^. Several studies have confirmed that the accumulation of neutrophils, NETs generation, and excessive production of serine protease are likely mechanisms that exacerbate various inflammatory diseases, including inflammatory bowel disease, asthma, atherosclerosis, diabetes mellitus, psoriasis, chronic obstructive pulmonary disease, rheumatoid arthritis, lung diseases, life-threatening sepsis, and cancer^19–22^. Recently, neutrophils were also linked with flu- or coronavirus-associated acute respiratory distress syndrome^23^. Accordingly, targeting neutrophil-mediated inflammation has gained attention as a promising strategy for management of inflammatory diseases^24^.

Enones are compounds possessing electrophilic Michael accepting α,β-unsaturated carbonyl functionalities that exhibit cytoprotective, anti-inflammatory, and antioxidant properties via regulation of cysteine-dependent signal transduction pathways^25^. In addition to their carbonyl group, some enones have phenolic hydroxyls that can reinstall redox homeostasis via directly scavenging various types of ROS^26–28^.

Several enone-based natural products, including food polyphenols, curcumin, butein, and isoliquiritigenin have demonstrated noticeable anti-inflammatory activities^29^^,^^30^. Licochalcone A, a constituent of liquorice, showed moderate inhibition of interleukins IL-β and IL-6 and tissue necrosis factor-α (TNF-α)^31^^,^^32^, while phloretin in apples diminished nuclear factor-κB (NF-κB) and TNF-α levels^33^. In addition, synthetic derivatives retaining the di-enone linker of the natural product curcumin or having contracted mono-enone linker, were reported as stimulators of transcription factor nuclear factor erythroid 2 related factor 2 (Nrf2), a master regulator of a multitude of anti-inflammatory and anti-oxidant downstream proteins^28^^,^^34^^,^^35^.

Moreover, many semisynthetic and patented synthetic chalcones exhibited a variety of other anti-inflammatory mechanisms, including inhibition of inducible nitric oxide synthase (iNOS) expression, suppression of prostanoid and proinflammatory cytokines production, direct neutralisation of ROS or induction of quinone oxidoreductase-1 (NQO-1) and antioxidant heme oxygenase-1 (HO-1) genes^36–38^. In addition, cyclooxygenase (COX), lipooxygenase (LOX), leukotriene D4 (LTD4), monocyte chemoattractant protein-1 (MCP-1), and intracellular cell adhesion molecule-1 (ICAM-1) were reported among the therapeutic targets that are potentially inhibited by enones^39^.

The present study identified enone derivatives as anti-inflammatory hits for attenuating neutrophilic inflammation via an in-house library screening. Structure–activity relationship (SAR)-guided optimisation of these hits granted non-cytotoxic agents that can effectively suppress neutrophilic production of SO anion only or both SO and elastase when triggered by fMLF. An immunoblotting assay for the most potent analogues revealed the inhibition of MAPKs and/or Akt signalling pathways as plausible anti-neutrophilic mechanisms.

## Results and discussion

### SAR for screened in-house library and compound design

Superoxide anions are the primary ROS whose excessive and prolonged production by overly activated neutrophils is a primary cause of host cell damage, leading to chronic inflammatory response^40^. Additionally, human neutrophil elastase, released from azurophilic granules during lysis, is a prominent component of the antipathogenic NETs^41^. Given the significant roles of elevated levels of elastase and SO anions in mediating inflammatory diseases^42–45^, and guided by the esteemed activities of natural and synthetic enone derivatives as antioxidant and anti-inflammatory agents, we aimed to screen an in-house library of 1,3-disubstituted prop-2-en-1-one analogues (**1**–**30**)^46–54^ for potential inhibitory effects on elastase release and SO production in fMLF/cytochalasin B (CB)-activated human neutrophils (Table 1). Cytochalasin B served as a priming agent cotreated together with the exogenous FPR1 stimulator fMLF. The screened library examined the influence of using aliphatic, aryl, or heteroaryl moieties at position 1 of the prop-2-en-1-one scaffold on the modulation of anti-inflammatory activity. In addition, the effect of employing aryl, heteroaryl, fused, or isolated bicyclic systems at position 3 on activity was explored.

**Table 1.** Effects of screened in-house compounds (**1–30**) on neutrophilic levels of superoxide anion and elastase upon stimulation by fMLF and CB.

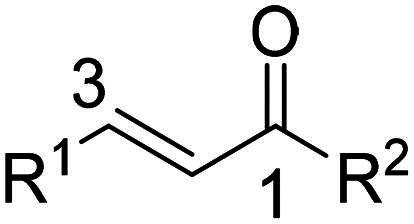						
			Superoxide anion		Elastase	
Cpd	R^1^	R^2^	Inhibition %	IC_50_ (μM)^a^	Inhibition %	IC_50_ (μM)^a^
**1**	4-Chlorophenyl	*t*-Bu	23.44 ± 7.96*		8.43 ± 4.28	
**2**	4-Chlorophenyl	Phenyl	94.7 ± 3.22***	3.01 ± 0.68	62.00 ± 6.31**	7.78 ± 0.96
**3**	4-Bromophenyl	*t*-Bu	25.04 ± 2.48***		9.09 ± 2.01*	
**4**	4-Trifluoromethylphenyl	*t*-Bu	11.72 ± 5.39		1.18 ± 0.98	
**5**	2-Trifluoromethylphenyl	2-Ethoxyphenyl	68.28 ± 8.99**	1.81 ± 0.22	8.13 ± 3.43	
**6**	4-Nitrophenyl	*t*-Bu	23.34 ± 2.30		^b^	
**7**	4-Nitrophenyl	Pyridin-2-yl	94.45 ± 2.89***	4.35 ± 0.30	78.10 ± 0.36***	6.17 ± 0.10
**8**	3-Nitrophenyl	*t*-Bu	10.31 ± 2.86*		4.89 ± 0.91**	
**9**	4-Cyanophenyl	*t*-Bu	20.93 ± 5.55*		3.76 ± 2.69	
**10**	*p*-Tolyl	*t*-Bu	8.56 ± 3.04*		3.91 ± 2.82	
**11**	4-*t*-Butoxyphenyl	*t*-Bu	16.81 ± 3.85*		12.11 ± 3.43*	
**12**	4-*t*-Butoxyphenyl	Furan-2-yl	27.31 ± 2.07***		19.16 ± 4.05**	
**13**	4-*t*-Butoxyphenyl	Pyrrol-2-yl	^b^		^b^	
**14**	3,5-Difluorophenyl	*t*-Bu	69.14 ± 6.71***	6.52 ± 0.57	12.00 ± 3.52*	
**15**	2,4-Dichlorophenyl	*t*-Bu	42.97 ± 2.78***		^b^	
**16**	3,4-Dichlorophenyl	*t*-Bu	16.49 ± 4.67*		10.19 ± 0.59***	
**17**	4-Chloro-3-fluorophenyl	*t*-Bu	40.63 ± 3.89***		9.13 ± 1.78**	
**18**	3-Fluoro-*p*-tolyl	*t*-Bu	31.33 ± 4.71**		7.77 ± 0.80***	
**19**	3-Fluoro-4-methoxyphenyl	*t*-Bu	2.87 ± 2.80		3.90 ± 1.68	
**20**	3-Fluoro-4-methoxyphenyl	*o*-tolyl	49.78 ± 5.82***		10.56 ± 2.28**	
**21**	3-Fluoro-4-methoxyphenyl	2,4-Dimethoxyphenyl	17.10 ± 3.58**		31.63 ± 5.88**	
**22**	2,3-Difluoro-4-methoxyphenyl	*t*-Bu	31.45 ± 3.36***		4.84 ± 2.66	
**23**	2,5-Difluoro-4-methoxyphenyl	*t*-Bu	8.10 ± 1.88*		2.50 ± 1.53	
**24**	5-Bromothiophen-2-yl	*t*-Bu	13.02 ± 6.88		25.68 ± 5.36**	
**25**	5-Bromothiophen-2-yl	2-Ethoxyphenyl	67.81 ± 3.19***	6.21 ± 0.36	26.85 ± 5.50**	
**26**	Naphthalen-2-yl	*t*-Bu	10.61 ± 3.34*		^b^	
**27**	Quinolin-2-yl	*t*-Bu	2.26 ± 0.97		5.21 ± 2.72	
**28**	Quinolin-2-yl	Phenyl	105.94 ± 1.73***	1.73 ± 0.07	99.08 ± 7.32***	2.17 ± 0.30
**29**	[1,1′-Biphenyl]-4-yl	*t*-Bu	2.23 ± 0.94		^b^	
**30**	[1,1′-Biphenyl]-4-yl	2-Ethoxyphenyl	8.69 ± 2.43*		15.81 ± 2.57**	
**LY294002** ^c^			101.58 ± 2.64***	2.02 ± 0.84	82.53 ± 3.76***	4.93 ± 2.48

Inhibition percentages (inhibition %) are assessed at 10 μM concentration. Results are provided as mean ± SEM (*n* = 3–5).

Table entities that showed more than 50% inhibition of either superoxide anion generation or elastase release or both are shaded.

*p < 0.05, **p < 0.01, and ***p < 0.001 in comparison with 0.1% DMSO induced by fMLF/CB (set as control with 0% inhibition).

^a^ Half-maximal inhibitory concentration (IC_50_).

^b^ 10 μM of compounds **6**, **13**, **15**, **26**, and **29** induced elastase release while 10 μM of compound **13** induced production of superoxide anion when subjected to cytochalasin B only.

^c^ Positive control.

The inhibitory percentages of both SO anion and elastase production were determined at a screening dose of 10 μM. Compounds attaining inhibition percentages higher than 50% were promoted to further determination of the respective IC_50_ values. The results of the primary screening are demonstrated in Table 1, upon which the following key SAR conclusions can be made:

(i) Fixing an aliphatic *tert*-butyl (*t*-Bu) group at position 1 of prop-2-en-1-one while exploring substituents at position 3 of various size, electronic effect, substitution pattern, and polarity yielded a potent hit (**14**) bearing a 3-(3,5-difluorophenyl). Compound **14** could potently inhibit fMLF-induced neutrophilic SO production with an IC_50_ of 6.52 ± 0.57 μM while elastase release was minimally inhibited. Neither the production of SO nor the release of elastase was noticeably affected when the 3-aryl was monosubstituted with electron-withdrawing (EW) groups (in compounds **1**, **3–4**, **6**, **8–9**) or electron-donating (ED) groups (in compounds **10** and **11**). Unlike compound **14**, all other tested disubstitution patterns of the 3-phenyl ring using EW groups (**15–17**) or combined EW and ED groups (**18–19** and **22–23**) resulted in diminished anti-neutrophilic activity. Similarly, enone’s position 3 substitution with the heteroaryl 5-bromo-2-thienyl (**24**), the bulkier fused aromatic systems (2-naphthyl in **26** and 2-quinolyl in **27**) or the biaryl system (4-biphenyl in **29**) demonstrated low inhibitory activities of fMLF-induced SO generation and elastase release where compounds **26** and **29** even induced elastase release at the 10 μM screening dose.

(ii) The use of 1-aryl/heteroaryl rings instead of the aliphatic 1-*t*-Bu granted a general positive impact on overall anti-neutrophilic activity. The use of 1-phenyl substituent in compounds **2** and **28** prompted a significant boost of potency which extended to inhibition of both SO production (**2**; IC_50_ = 3.01 ± 0.68 μM and **28**; IC_50_ = 1.73 ± 0.07 μM) and elastase secretion (**2**; IC_50_ = 7.78 ± 0.96 μM and **28**; IC_50_ = 2.17 ± 0.30 μM) when compared to their 1-*t*-Bu substituted congeners (**1** and **27**, respectively). A similar remarkable increase in potency was achieved when the 1-*t*-Bu substituent in compound **6** was modified to 2-pyridyl, the more polar isostere of phenyl, in compound **7**. On the contrary, the anti-neutrophilic potency of compound **11** did not greatly increase upon employing the heteroaryl 2-furyl (in compound **12**) while activity was totally abolished when 2-pyrrolyl (in compound **13**) was utilised. Substitution of 1-aryl with an ED 2-ethoxy group substantially enhanced the SO generation inhibitory activity of compounds **4** and **24** to give the potent compounds **5** and **25** with IC_50_ values of 1.81 ± 0.22 μM and 6.21 ± 0.36 μM, respectively, yet the ability to inhibit elastase release was not improved by both analogues. However, a similar 1-phenyl substitution with 2-ethoxy group (compare **29** and **30**), or other ED groups like 2-methyl (compare **19** and **20**) and 2,4-dimethoxy (compare **19** and **21**) resulted in a less pronounced increase of anti-inflammatory potency.

Interestingly, compounds **6**, **13**, **15**, **26**, and **29** induced elastase release while compound **13** induced also SO anion generation in the presence of solely CB, indicating an immune-modulating effect of these compounds, in particular, the 3-(4-*tert*-butoxyphenyl) and 1-(pyrrol-2-yl) derivative (**13**).

**Figure 2.**
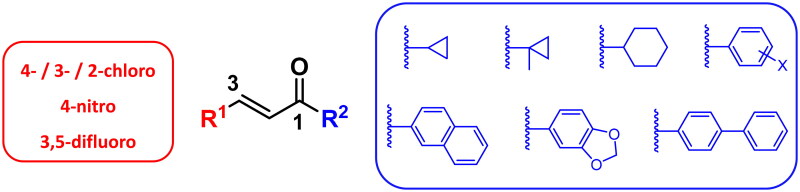
The adopted structural diversification for the synthesised prop-2-en-1-one derivatives.

In summary, 1-aryl/heteroaryl substituted enones were found superior as inhibitors of neutrophil mediated inflammation compared to 1-*t*-Bu substituted analogues. Furthermore, substituting position 3 of prop-2-en-1-one scaffold with 2-quinolyl or EW substituted aryl/heteroaryl moieties granted the highest overall anti-inflammatory potency where levels of neutrophilic SO and elastase were significantly suppressed. Accordingly, we planned to synthesise a focused library of 1-substituted-3-phenyl prop-2-en-1-one analogues where the most optimum EW substituents on 3-phenyl during the screening stage (namely; 4-nitro and 4-chloro) were primarily employed. The influence of changing the position of the EW group on the 3-phenyl ring was also investigated. In addition, it seemed promising to adopt 3,5-difluoro substitution of the 3-phenyl since it granted the highest SO generation inhibitory activity among all screened 1-*t*-Bu substituted analogues. Further diversification was attained via examining the effect of position 1 substitution with various alicyclic, aryl, fused and isolated bicyclic systems on potency. Figure 2 summarises all planned structural modifications.

### Chemistry

**Scheme 1.**
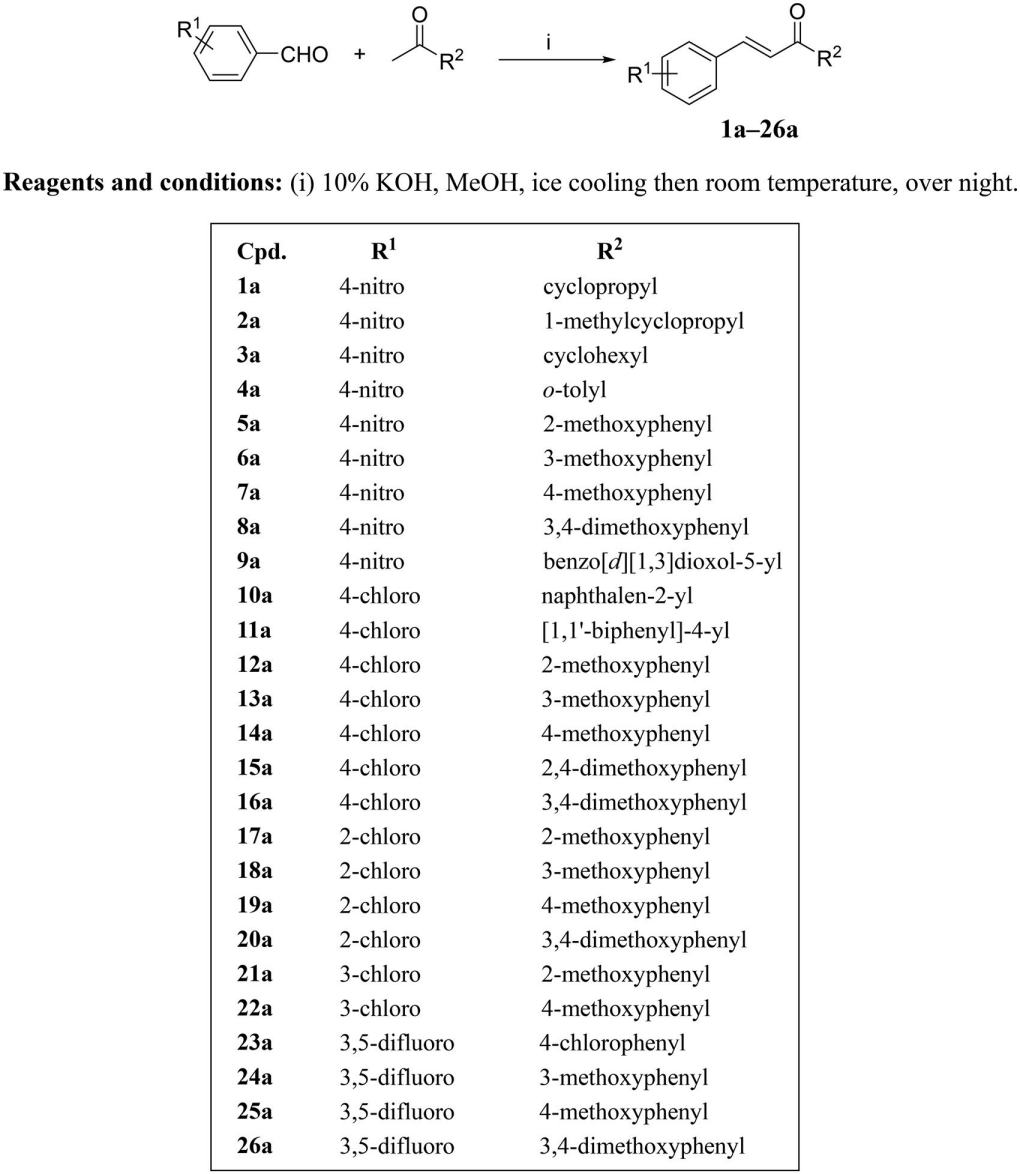
Synthesis of prop-2-en-1-one derivatives **(1a–26a).**

The intended enone derivatives were synthesised through a Claisen–Schmidt condensation reaction between aromatic aldehydes and suitable ketones, employing 10% aqueous KOH in methanol. This process produced compounds (**1a–26a**) in varying yields, as outlined in Scheme 1.

### Biological evaluation

All synthesised 1-substituted 3-aryl prop-2-en-1-one derivatives (**1a–26a**) were evaluated for their ability to inhibit neutrophilic production of SO anion and secretion of elastase upon stimulation by fMLF and CB. As implemented in the in-house library screening stage, the biological evaluation of tested compounds was accomplished in two steps: a primary screening was conducted at a concentration of 10 μM followed by IC_50_ determination for compounds exceeding 50% inhibition percentages. The results are presented in Table 2.

**Table 2.** Effects of prop-2-en-1-one derivatives (**1a–26a**) on neutrophilic levels of superoxide anion and elastase upon stimulation by fMLF and CB.

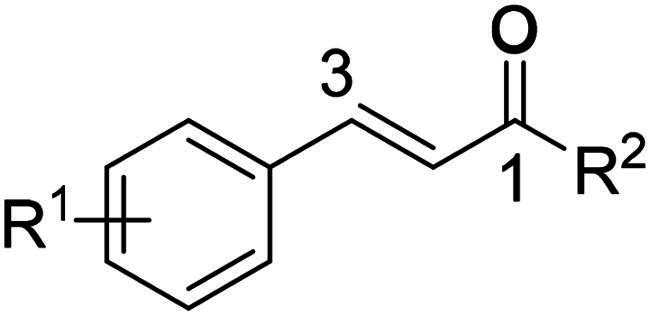						
			Superoxide anion		Elastase	
Cpd	R^1^	R^2^	Inhibition %	IC_50_ (μM)^a^	Inhibition %	IC_50_ (μM)^a^
**1a**	4-Nitro	Cyclopropyl	20.52 ± 4.05		2.36 ± 3.30	
**2a**	4-Nitro	1-Methylcyclopropyl	8.75 ± 5.03		8.39 ± 2.25*	
**3a**	4-Nitro	Cyclohexyl	11.42 ± 1.60**		11.94 ± 4.68	
**4a**	4-Nitro	*o*-Tolyl	100.18 ± 2.65***	4.55 ± 0.1	63.75 ± 5.98***	7.22 ± 1.16
**5a**	4-Nitro	2-Methoxyphenyl	101.74 ± 1.92***	3.77 ± 0.33	75.57 ± 5.02***	6.25 ± 0.52
**6a**	4-Nitro	3-Methoxyphenyl	99.69 ± 0.18***	1.23 ± 0.27	84.47 ± 5.73***	2.22 ± 0.12
**7a**	4-Nitro	4-Methoxyphenyl	59.67 ± 1.41***	6.90 ± 0.67	63.78 ± 6.59***	6.33 ± 1.15
**8a**	4-Nitro	3,4-Dimethoxyphenyl	80.97 ± 2.57***	2.72 ± 0.35	56.91 ± 3.22***	8.31 ± 0.78
**9a**	4-Nitro	Benzo[*d*][1,3]dioxol-5-yl	18.11 ± 3.34**		16.68 ± 6.07	
**10a**	4-Chloro	Naphthalen-2-yl	14.22 ± 6.87		33.19 ± 6.47**	
**11a**	4-Chloro	[1,1′-Biphenyl]-4-yl	^b^		^b^	
**12a**	4-Chloro	2-Methoxyphenyl	93.77 ± 3.02***	3.47 ± 0.60	62.16 ± 5.35***	7.56 ± 1.05
**13a**	4-Chloro	3-Methoxyphenyl	101.72 ± 0.44***	1.37 ± 0.73	91.92 ± 1.63***	3.30 ± 0.68
**14a**	4-Chloro	4-Methoxyphenyl	58.92 ± 3.26	6.09 ± 7.19	5.49 ± 4.32	
**15a**	4-Chloro	2,4-Dimethoxyphenyl	13.94 ± 2.43		4.42 ± 3.90	
**16a**	4-Chloro	3,4-Dimethoxyphenyl	68.15 ± 7.74	5.53 ± 1.48	22.90 ± 7.11	
**17a**	2-Chloro	2-Methoxyphenyl	99.05 ± 0.64***	2.95 ± 0.40	75.78 ± 6.19***	5.36 ± 0.67
**18a**	2-Chloro	3-Methoxyphenyl	47.64 ± 5.41***		31.34 ± 7.29*	
**19a**	2-Chloro	4-Methoxyphenyl	80.15 ± 1.98***	5.23 ± 0.62	37.57 ± 3.54**	
**20a**	2-Chloro	3,4-Dimethoxyphenyl	95.57 ± 1.14***	3.55 ± 0.56	67.66 ± 5.37***	6.79 ± 0.72
**21a**	3-Chloro	2-Methoxyphenyl	89.90 ± 2.38***	3.79 ± 0.69	80.43 ± 4.44***	5.63 ± 0.51
**22a**	3-Chloro	4-Methoxyphenyl	65.18 ± 3.73***	6.60 ± 0.60	36.15 ± 6.48*	
**23a**	3,5-Difluoro	4-Chlorophenyl	96.04 ± 0.09***	2.03 ± 0.27	82.97 ± 2.64***	2.32 ± 0.26
**24a**	3,5-Difluoro	3-Methoxyphenyl	3.12 ± 5.02		37.14 ± 7.65**	
**25a**	3,5-Difluoro	4-Methoxyphenyl	78.19 ± 9.5**	4.70 ± 0.60	20.91 ± 3.64**	
**26a**	3,5-Difluoro	3,4-Dimethoxyphenyl	92.38 ± 2.19***	1.56 ± 0.27	20.22 ± 3.19**	

Inhibition percentages (inhibition %) are assessed at 10 μM concentration. Results are provided as mean ± SEM (*n* = 3–5).

Table entities that showed more than 50% inhibition of either superoxide anion generation or elastase release or both are shaded.

*p < 0.05, **p < 0.01, and ***p < 0.001 in comparison with 0.1% DMSO induced by fMLF/CB (set as control with 0% inhibition).

^a^ Half-maximal inhibitory concentration (IC_50_).

^b^ 10 μM of compound **11a** raised levels of both superoxide and elastase when subjected to cytochalasin B only.

#### SAR for superoxide anion production inhibition

##### Modifications at position 1 of enone

*(a) Aliphatic alkyl vs. alicyclic vs. monocyclic/bicyclic aromatic moieties*. It was deduced from the screening stage that the use of the aliphatic alkyl moiety (1-*t*-Bu) was generally detrimental for SO generation inhibitory activity. Thus, we needed first to investigate if other types of aliphatic groups could improve activity. Compared to compound **6**, the alicyclic groups (cyclopropyl, 1-methylcyclopropyl, and cyclohexyl) in compounds **1a–3a** did not improve the inhibition of neutrophilic SO production, indicating that further aliphatic substitutions at that position are unlikely to be beneficial. Next, we explored the effect of employing enlarged aromatic systems like 2-naphthyl or 4-biphenyl at position 1 of enone on inhibitory activity (compare **10a** and **11a**, respectively, to compound **2**). This structural extension led to a marked deterioration of inhibitory activity to the extent that **11a** became an inducer rather than an inhibitor at the used 10 μM concentration. At this stage, it was judicious to mainly pursue our further attempts for optimisation of SO generation inhibitory activity with 1-phenyl substituted prop-2-en-1-one derivatives (chalcones).

*(b) Influence of 1-phenyl substituents*. We started examining the influence of different substitution patterns of 1-phenyl on SO generation inhibitory activity by comparing the lipophilic ED group *o*-methyl (in compound **4a**) with the more polar *o*-methoxy group (in compound **5a**) that were found almost equipotent with a slight superiority for the methoxy substituent. Afterwards, methoxy groups were probed at different positions of the 1-phenyl ring where all *ortho*-methoxylated derivatives exhibited higher SO generation inhibitory potency than their *para*-methoxylated analogues, irrespective of the 3-phenyl substituent (compare IC_50_s of compounds **5a**, **12a**, **17a**, and **21a** to those of compounds **7a**, **14a**, **19a**, and **22a**, respectively). On the other hand, the potency of the *meta*-methoxylated derivatives was apparently dictated by the nature of the 3-phenyl substituent where **6a** and **13a** exhibited the utmost suppression of SO with IC_50_ of 1.23 ± 0.27 and 1.37 ± 0.73 μM, respectively, while the inhibitory activities of **18a** and **24a** were notably diminished. Disubstitution of 1-phenyl with 3,4-dimethoxy groups resulted in markedly potent SO generation inhibitors (compounds **8a**, **16a**, **20a**, and **26a**), irrespective of the 3-phenyl substituent while bridging the 3,4-dimethoxy with a methylene spacer (compare **8a** to **9a**) or the use of 2,4-dimethoxy groups (compare **15a** to **16a**) abolished the activity.

##### Modifications at position 3 of enone

Generally, all investigated 3-phenyl substituents were well tolerated and efficiently suppressed fMLF/CB-driven neutrophilic production of SO anion. No subtle differences in potency were observed upon replacing the stronger EW 4-nitro with the more lipophilic weaker EW 4-chloro or with 3,5-difluoro groups. Similarly, the position of the chloro substituent on the 1-phenyl ring did not greatly affect SO generation inhibitory potency.

#### SAR for elastase release suppression

##### Modifications at position 1 of enone

In great analogy to the SAR for SO generation inhibition, none of the examined alicyclic, fused aromatic, or biaryl systems at position 1 of enone could attain better suppression of neutrophilic elastase compared to the screened 1-*t*-Bu substituted congener (compound **6**). However, in contrast to SAR for SO generation inhibition, varying the electronic nature or pattern of 1-phenyl substituents prompted noticeable differences in elastase release inhibitory activity. 1-Phenyl substitution with *o*-methyl (**4a**) or *o*-methoxy (**5a**, **12a**, **17a**, and **21a**) groups yielded potent elastase release inhibitors of comparable IC_50_ values, irrespective of the type of 3-phenyl substituent. On the other hand, the elastase release inhibitory activity of *m*-methoxy, *p*-methoxy or 3,4-dimethoxy substituted derivatives greatly varied based on the 3-phenyl substituent type where combinations with 3-(4-nitrophenyl) in particular consistently retained activity. Furthermore, replacing the ED *p*-methoxy (in compound **25a**) with the EW *p*-chloro substituent (in compound **23a**) prompted a marked boost of activity with an IC_50_ reaching 2.32 ± 0.26 μM.

##### Modifications at position 3 of enone

Unlike the SAR for SO generation inhibition, the nature and position of 3-phenyl substituents substantially affected elastase release inhibitory activity and were not similarly tolerated. All 4-nitro substituted derivatives (**4a–8a**) exhibited high potencies quite tolerating a variety of 1-phenyl substituents. In contrast, the potency of *o*-, *m*-, and *p*-chloro substituted derivatives was primarily dependent on the type of 1-phenyl substituent. Moreover, 3,5-difluoro substitution led to marked deterioration of elastase release inhibitory activity especially when combined with methoxylated 1-phenyl rings (compounds **24a–26a**).

**Figure 3.**
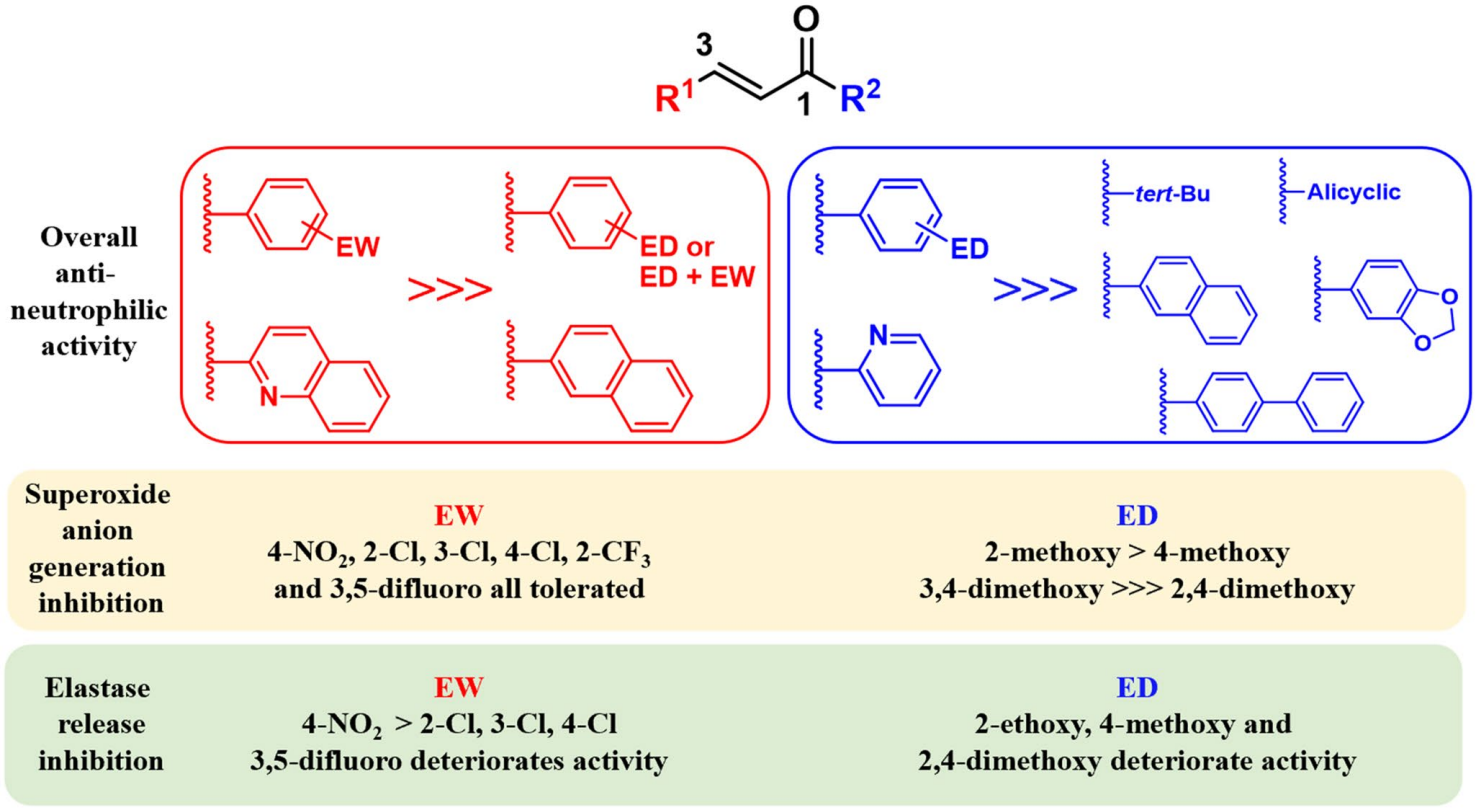
Summary for the SAR of screened and synthesised prop-2-en-1-one derivatives. EW: electron-withdrawing; ED: electron-donating.

Collectively, the results shown in Tables 1 and 2 and the SAR summary depicted in Figure 3 for both screened and synthesised prop-2-en-1-one derivatives proposed two clusters of neutrophil-targeting anti-inflammatory agents that possess distinct pharmacological profiles based on the deployed structural features; (i) compounds bearing unsubstituted, mono- or di-methoxylated 1-aryl/heteroaryl rings along with 3-heteroaryl or chlorinated/*para*-nitrated 3-phenyl rings can be regarded as suppressors of both SO and elastase levels with compound **6a** being the most potent. (ii) Compounds whose 1-phenyl is substituted with 2-ethoxy, 4-methoxy, or 3,4-dimethoxy groups and their 3-aryl/heteroaryl rings are substituted with EW groups (basically 3,5-difluoro) exhibited sole inhibition of SO anion production with compound **26a** being the most potent.

#### Evaluating the effect of prop-2-en-1-one derivatives on human neutrophils viability

**Figure 4.**
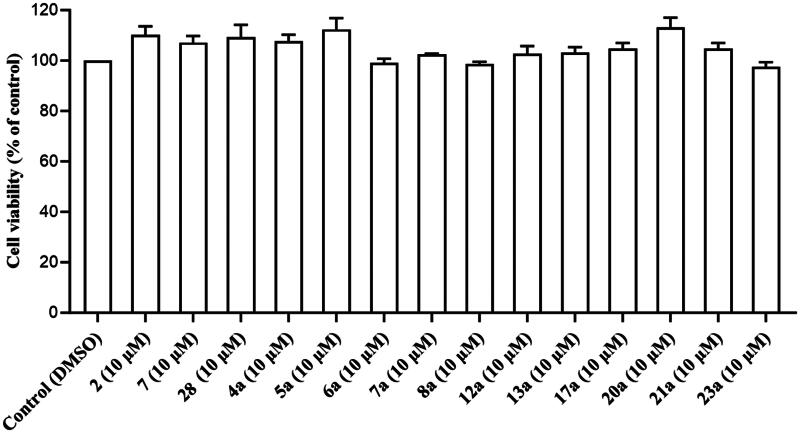
Potent prop-2-en-1-one derivatives do not impact human neutrophils survival. Human neutrophils were subjected to DMSO (0.1%), or tested compound (10 μM) for 15 min. LDH levels in the cell-free supernatant were used for estimating cell viability. Cells were lysed with 0.1% Triton X-100 for 30 min at 37 °C in order to assess total LDH release. All data are provided as the mean ± SEM (*n* = 3).

To assess the suitability of the enone scaffold as potential anti-neutrophilic agents, all analogues that demonstrated dual suppressive effects on neutrophilic SO and elastase levels from both screened and synthesised series were additionally investigated for any possible cytotoxic effects on human neutrophils. Ten micromolars of all tested prop-2-en-1-one derivatives prompted minimal release of lactate dehydrogenase (LDH) in human neutrophils indicating their intactness (Figure 4). Based on their low cytotoxicity, enone derivatives can be regarded as promising anti-inflammatory agents especially in pathologies that involve significant neutrophil activation reactions.

#### Anti-neutrophilic molecular mechanism elucidation: chalcones 6a and 26a impede MAPKs and Akt phosphorylation in FPR1 agonist-triggered neutrophils

The intricate intracellular signalling pathways governing neutrophil activation remain elusive and highly complex. Protein kinases such as the MAPK cascades (JNK, ERK, and p38) and the PI3K/Akt signalling pathways are influenced by fMLF receptor interplay and play pivotal roles in orchestrating various intracellular and extracellular events. Particularly, the PI3K pathway is well-established for its significant role in neutrophil activation triggered by GPCR agonists such as fMLF. Akt is subjected to phosphorylation and activation downstream PI3K activation^55^.

MAPKs or Akt phosphorylation and activation modulate neutrophilic actions, such as adhesion, chemotaxis, degranulation, and oxidative burst^56^. In the further experiment, we aimed to identify the particular target(s) along those signal transduction cascades in activated human neutrophils that are potentially affected by the most potent anti-neutrophilic prop-2-en-1-one derivatives synthesised in this work. Compound **6a**, the most potent inhibitor of both fMLF-induced SO generation and elastase release, and compound **26a**, the most potent inhibitor of SO generation alone, were selected for such investigation with the aim of revealing the molecular mechanism(s) that dictated their different pharmacological profiles.

**Figure 5.**
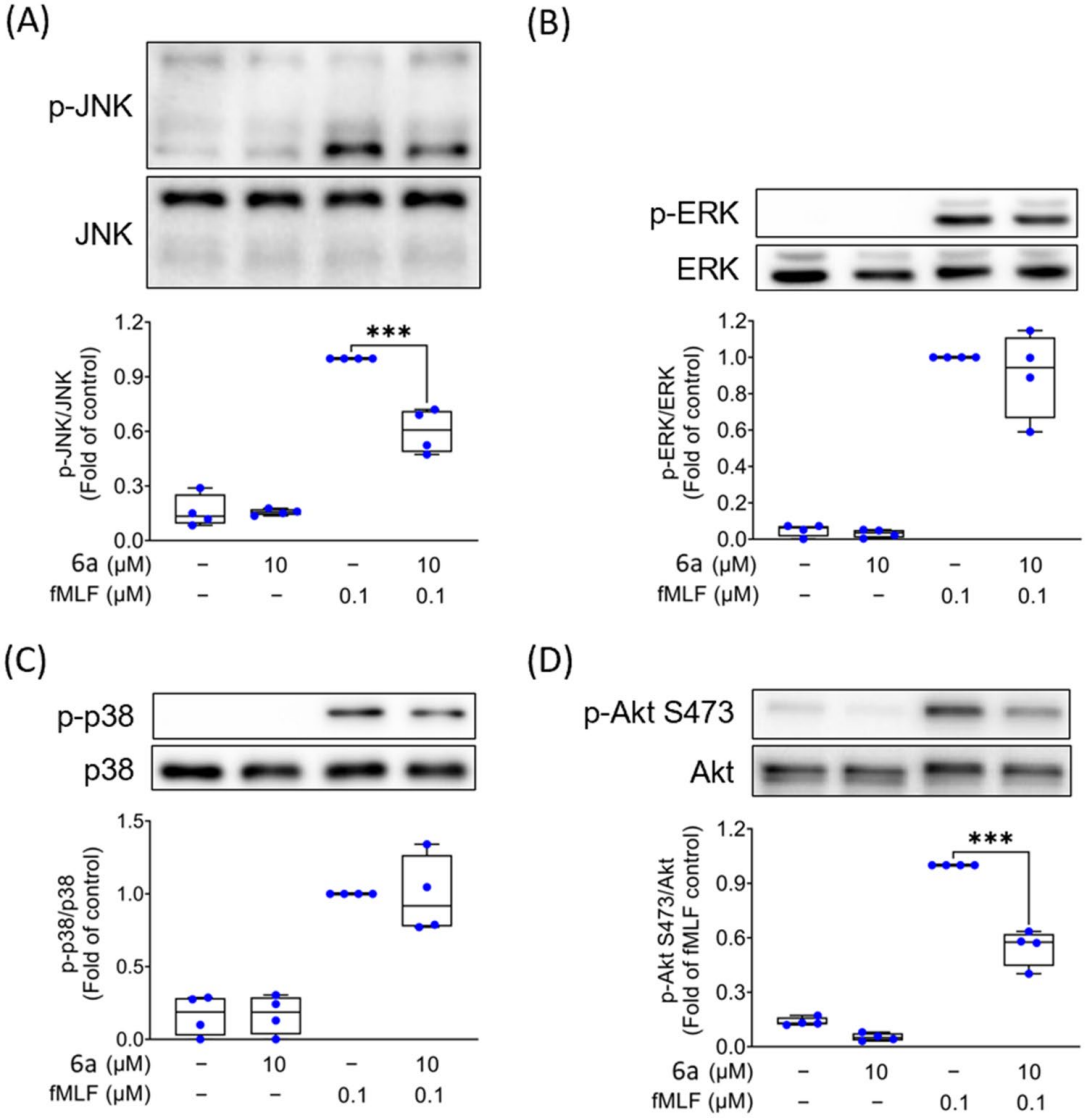
Compound **6a** diminishes JNK and Akt phosphorylation in neutrophils activated by fMLF. Human neutrophils were subjected to either DMSO (0.1%) or compound **6a** (10 μM) for 5 min, followed by stimulation with or without fMLF (0.1 μM) for 30 s. Subsequently, the cells were lysed and underwent immunoblotting analysis. The phosphorylation of (A) JNK, (B) ERK, (C) p38 MAPK, and (D) Akt was evaluated using antibodies that recognise both the total and phosphorylated forms of these proteins. The data were normalised to the levels of the total assessed protein and are presented as the mean ± SEM. ***p < 0.001 in comparison with DMSO + fMLF (*n* = 3).

**Figure 6.**
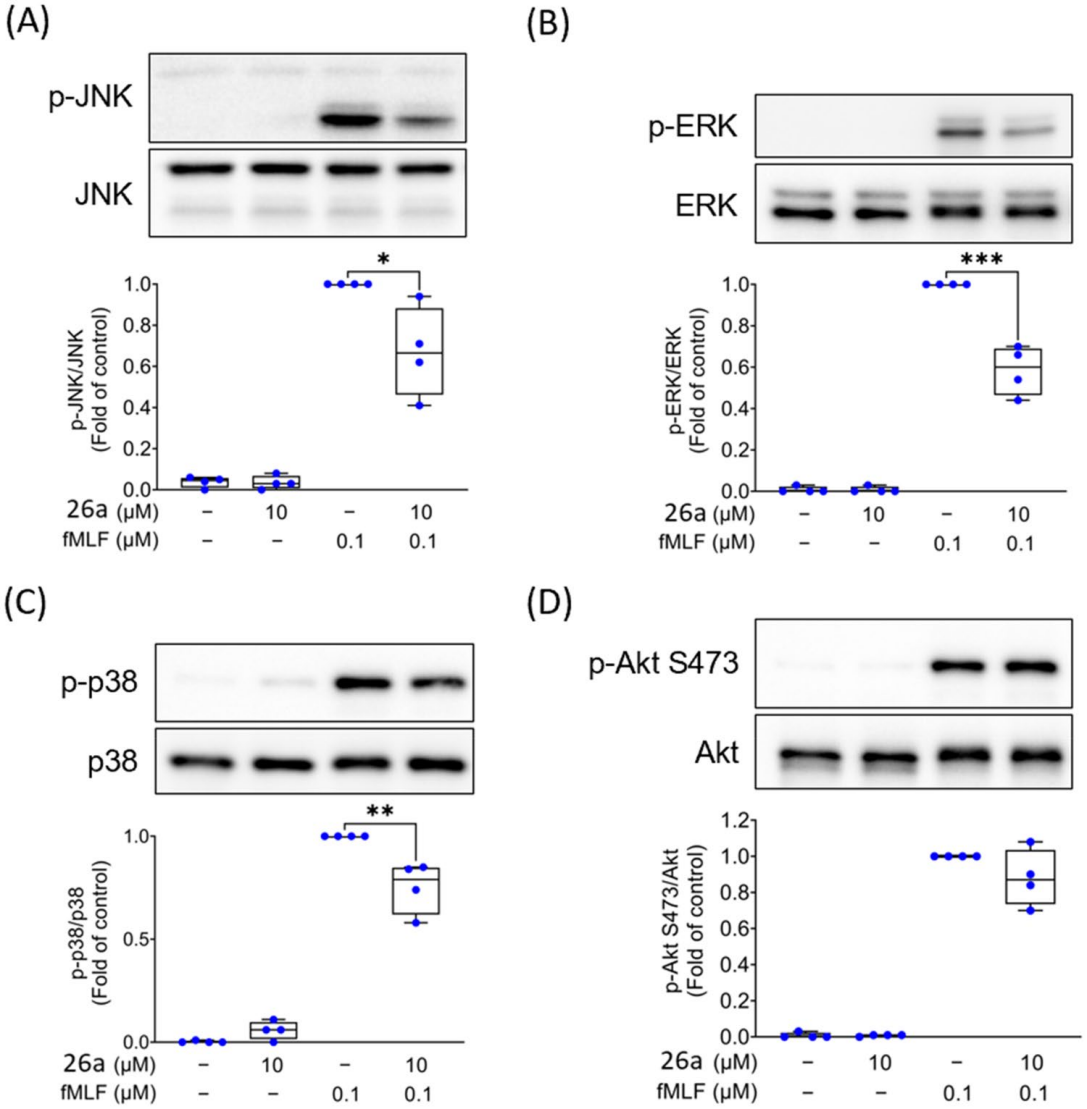
Compound **26a** diminishes JNK, ERK, and p38 MAPK phosphorylation in neutrophils activated by fMLF. Human neutrophils were subjected to either DMSO (0.1%) or compound **26a** (10 μM) for 5 min, followed by stimulation with or without fMLF (0.1 μM) for 30 s. Subsequently, the cells were lysed and underwent immunoblotting analysis. The phosphorylation of (A) JNK, (B) ERK, (C) p38 MAPK, and (D) Akt was evaluated using antibodies that recognise both the total and phosphorylated forms of these proteins. The data were normalised to the levels of the total assessed protein and are presented as the mean ± SEM. *p < 0.05; **p < 0.01; ***p < 0.001 in comparison with DMSO + fMLF (*n* = 3).

As demonstrated in Figures 5 and 6, phosphorylated forms of MAPKs (JNK, ERK, and p38) and Akt protein were activated in human neutrophils treated with 0.1 μM of fMLF. Immunoblotting experiments revealed that compound **6a** significantly suppressed phosphorylation of JNK and Akt in fMLF-activated neutrophils while it did not alter the phosphorylation of ERK and p38 (Figure 5). On the other hand, compound **26a** did not affect fMLF-triggered phosphorylation of Akt but markedly diminished phosphorylation of all tested MAPKs: JNK, ERK, and p38 (Figure 6). In contrast to compound **26a**, compound **6a** could uniquely inhibit both fMLF-induced elastase release and Akt phosphorylation which is in line with the previous literature, where Akt played an important role in suppressing degranulation (elastase release) in activated neutrophils^17^^,^^57^. Activating PI3K/Akt has been shown to impose several neutrophilic events such as oxidative burst and degranulation^58^. Nevertheless, MAPKs appear to play predominant role in regulating SO anion production in fMLF-triggered neutrophils.

It can be concluded that **6a** blocked FPR1 agonist-induced oxidative burst and degranulation via suppression of Akt and JNK phosphorylation. Nevertheless, **26a** blocked solely FPR1 agonist-induced oxidative burst by inhibition of MAPKs (JNK, ERK, and p38), where Akt was not affected.

## Conclusions

To the best of our knowledge, the current study demonstrates for the first time 1,3-disubstituted prop-2-en-1-one derivatives as promising neutrophil targeting anti-inflammatory agents. The most potent suppressor of both SO anion and elastase levels in fMLF-stimulated neutrophils was compound **6a** with IC_50_ of 1.23 ± 0.27 and 2.22 ± 0.12 μM, respectively. Compound **6a** possessed a 1-(3-methoxyphenyl) combined with 3-(4-nitrophenyl), yet the enone scaffold could potentially accommodate various 1-ED and 3-EW substituted aryl/heteroaryl moieties without losing its dual anti-inflammatory capability. Compound **26a**, bearing a 1-(3,4-dimethoxyphenyl) and 3-(3,5-difluorophenyl) was the most potent among analogues with sole SO generation inhibitory effect (IC_50_ of 1.56 ± 0.27 μM). The anti-neutrophilic activity of compound **6a** was mainly mediated by the blockade of JNK, and Akt signalling pathways, while **26a** could efficiently suppress JNK, ERK, and p38 MAPK cascades, suggesting a role of Akt pathway in fMLF-triggered elastase release. Moreover, all 14 potent inhibitors of both fMLF-induced SO generation and elastase release showed no impact on human neutrophils survival. Taking into consideration the potency and low cytotoxicity of enone derivatives and the established roles of elevated neutrophil elastase and ROS levels in pathogenesis of various inflammatory diseases related to immune failure or excess of cytokine storm. Enones can thus serve as propitious core for developing potential inhibitors of neutrophil-mediated inflammation.

## Experimental

### Chemistry

Solvents and reagents were obtained from commercial sources and used as received. The structures of the novel enones were determined using ^1^H and ^13^C NMR spectroscopy, while melting points helped verify the identity of previously described enones. The purity of all tested compounds, as listed in Tables 1 and 2, was confirmed by UHPLC linked to mass spectrometry, with a purity level of at least 95%. ^1^H and ^13^C NMR spectra were recorded on a Varian Mercury 400 spectrometer (Palo Alto, CA), using the residual protonated DMSO signals as the reference for chemical shifts. Mass spectrometric analysis was conducted using a Waters ACQUITY Xevo TQD system (Milford, MA), following previously reported methods^59^. Melting points were measured using a Buchi B-540 melting point apparatus (Flawil, Switzerland) and are presented without correction.

#### General procedure for enone synthesis

A solution containing 10 mmol of the designated ketone in 50 mL of methanol was cooled using an ice bath, followed by the addition of 30 mL of 10% aqueous KOH. Subsequently, 10 mmol of the specified aromatic aldehyde was added slowly. The mixture was left to stir at room temperature overnight. If a solid product formed, it was filtered out and washed three times with a 5:3 methanol/water mixture before drying. In cases where the product was oily, the reaction mixture was extracted with four 20 mL portions of CH_2_Cl_2_. The combined organic extracts were washed with water, filtered through anhydrous MgSO_4_, and then evaporated under reduced pressure to obtain the product^60^. All desired products were obtained pure with the exception of compounds **5** and **24** that needed further purification using column chromatography (CC).

##### (E)-1-(4-Chlorophenyl)-4,4-dimethylpent-1-en-3-one (1)

*p*-Chlorobenzaldehyde and pinacolone were used as reactants to give **1** (80% yield) as a white solid with a mp 85–85.5 °C; MS (ESI): *m/z* = 223.3 (M + H)^+^^47^.

##### (E)-3-(4-Chlorophenyl)-1-phenylprop-2-en-1-one (2)

*p*-Chlorobenzalde­hyde and acetophenone were used as reactants to give **2** (88% yield) as a yellow solid with a mp 113–114 °C; MS (ESI): *m/z* = 243.2 (M + H)^+^^50^.

##### (E)-1-(4-Bromophenyl)-4,4-dimethylpent-1-en-3-one (3)

*p*-Bromobenzald­e­hyde and pinacolone were used as reactants to give **3** (78% yield) as a yellowish white solid with a mp 101 °C; MS (ESI): *m/z* = 267.3 (M + H)^+^^47^.

##### (E)-4,4-Dimethyl-1-(4-(trifluoromethyl)phenyl)pent-1-en-3-one (4)

*p*-(Trifluoromethyl)benzaldehyde and pinacolone were used as reactants to give **4** (90.5% yield) as a white solid with a mp 70–71 °C; MS (ESI): *m/z* = 257.3 (M + H)^+^^48^.

##### (E)-1-(2-Ethoxyphenyl)-3-(2-(trifluoromethyl)phenyl)prop-2-en-1-one (5)

*o*-(Trifluoromethyl)benzaldehyde and 2′-ethoxyacetophenone were used as reactants and the product was purified using CC (DCM/hexane 6:4) to give **5** (72% yield) as a white solid with a mp 70.5–71.5 °C; ^1^H NMR (400 MHz, CDCl_3_) *δ* 7.72–7.59 (m, 7H), 7.52–7.44 (m, 1H), 7.07–6.96 (m, 2H), 4.15 (q, *J* = 7.0 Hz, 2H), 1.43 (t, *J* = 7.0 Hz, 3H); ^13^C NMR (101 MHz, CDCl_3_) *δ* 192.18, 158.07, 140.12, 138.93 (d, ^3^*J*_C-F_ = 1.4 Hz), 133.66, 131.59 (d, ^2^*J*_C-F_ = 32.6 Hz), 130.92, 129.53, 128.97, 128.41, 125.99, 125.97 (d, ^3^*J*_C-F_ = 11.4 Hz), 125.95, 124.04 (d, ^1^*J*_C-F_ = 272.2 Hz), 120.98, 112.73, 64.39, 15.02; MS (ESI): *m/z* = 321.3 (M + H)^+^.

##### (E)-4,4-Dimethyl-1-(4-nitrophenyl)pent-1-en-3-one (6)

*p-*Nitrobenzal­dehyde and pinacolone were used as reactants to give **6** (82% yield) as a yellow solid with a mp 125–126 °C; MS (ESI): *m/z* = 234.4 (M + H)^+^^47^.

##### (E)-3-(4-Nitrophenyl)-1-(pyridin-2-yl)prop-2-en-1-one (7)

*p-*Nitrobenzaldehyde and 2-acetylpyridine were used as reactants to give **7** (74% yield) as a yellow solid with a mp 153–155 °C; MS (ESI): *m/z* = 255.3 (M + H)^+^^51^.

##### (E)-4,4-Dimethyl-1-(3-nitrophenyl)pent-1-en-3-one (8)

*m*-Nitrobenzaldehyde and pinacolone were used as reactants to give **8** (60% yield) as a yellow solid with a mp 92–93 °C; MS (ESI): *m/z* = 234.3 (M + H)^+^^47^.

##### (E)-4-(4,4-Dimethyl-3-oxopent-1-en-1-yl)benzonitrile (9)

4-Formylbenzonitrile and pinacolone were used as reactants to give **9** (92% yield) as a white solid with a mp 131–133 °C; MS (ESI): *m/z* = 214.3 (M + H)^+^^46^.

##### (E)-4,4-Dimethyl-1-(p-tolyl)pent-1-en-3-one (10)

*p*-Tolualdehyde and pinacolone were used as reactants to give **10** (79% yield) as a white solid with a mp 79–80 °C; MS (ESI): *m/z* = 203.3 (M + H)^+^^47^.

##### (E)-1-(4-(tert-Butoxy)phenyl)-4,4-dimethylpent-1-en-3-one (11)

*p*-(*Tert*-butoxy)benzaldehyde and pinacolone were used as reactants to give **11** (77% yield) as a yellow solid with a mp 110–111 °C; MS (ESI): *m/z* = 261.4 (M + H)^+^^46^.

##### (E)-3-(4-(tert-Butoxy)phenyl)-1-(furan-2-yl)prop-2-en-1-one (12)

*p*-(*Tert*-butoxy)benzaldehyde and 2-acetylfuran were used as reactants to give **12** (76% yield) as a yellow solid with a mp 132.8–134.8 °C; MS (ESI): *m/z* = 271.4 (M + H)^+^^52^.

##### (E)-3-(4-(tert-Butoxy)phenyl)-1-(1H-pyrrol-2-yl)prop-2-en-1-one (13)

*p-*(*Tert*-butoxy)benzaldehyde and 2-acetylpyrrole were used as reactants to give **13** (84% yield) as a yellowish white solid with a mp 116.5–118.5 °C; MS (ESI): *m/z* = 270.4 (M + H)^+^^46^.

##### (E)-1-(3,5-Difluorophenyl)-4,4-dimethylpent-1-en-3-one (14)

3,5-Difluorobenzaldehyde and pinacolone were used as reactants to give **14** (89.6% yield) as a yellowish orange solid with a mp 71–72 °C; MS (ESI): *m/z* = 225.3 (M + H)^+^^48^.

##### (E)-1-(2,4-Dichlorophenyl)-4,4-dimethylpent-1-en-3-one (15)

2,4-Dichlorobenzaldehyde and pinacolone were used as reactants to give **15** (85% yield) as a yellow solid with a mp 103–104 °C; MS (ESI): *m/z* = 257.3 (M + H)^+^^53^.

##### (E)-1-(3,4-Dichlorophenyl)-4,4-dimethylpent-1-en-3-one (16)

3,4-Dichlorobenzaldehyde and pinacolone were used as reactants to give **16** (81% yield) as a yellow solid with a mp 94–96 °C; MS (ESI): *m/z* = 257.3 (M + H)^+^^49^.

##### (E)-1-(4-Chloro-3-fluorophenyl)-4,4-dimethylpent-1-en-3-one (17)

4-Chloro-3-fluorobenzaldehyde and pinacolone were used as reactants to give **17** (91% yield) as a yellow solid with a mp 98.5–99.2 °C; ^1^H NMR (400 MHz, CDCl_3_) *δ* 7.51 (d, *J* = 15.5 Hz, 1H), 7.39–7.17 (m, 3H), 7.02 (d, *J* = 15.5 Hz, 1H), 1.17 (s, 9H); ^13^C NMR (101 MHz, CDCl_3_) *δ* 203.87, 158.40 (d, ^1^*J*_C-F_ = 249.6 Hz), 140.56 (d, ^4^*J*_C-F_ = 2.0 Hz), 135.75 (d, ^3^*J*_C-F_ = 6.9 Hz), 131.16, 125.04 (d, ^3^*J*_C-F_ = 3.4 Hz), 122.82 (d, ^2^*J*_C-F_ = 17.8 Hz), 122.41, 115.54 (d, ^2^*J*_C-F_ = 21.5 Hz), 43.48, 26.34; MS (ESI): *m/z* = 241.3 (M + H)^+^.

##### (E)-1-(3-Fluoro-4-methylphenyl)-4,4-dimethylpent-1-en-3-one (18)

3-Fluoro-4-methylbenzaldehyde and pinacolone were used as reactants to give **18** (88.9% yield) as a white solid with a mp 70–71 °C; MS (ESI): *m/z* = 221.3 (M + H)^+^^48^.

##### (E)-1-(3-Fluoro-4-methoxyphenyl)-4,4-dimethylpent-1-en-3-one (19)

3-Fluoro-4-methoxybenzaldehyde and pinacolone were used as reactants to give **19** (94% yield) as a yellowish white solid with a mp 84.6 °C; MS (ESI): *m/z* = 237.3 (M + H)^+^^46^.

##### (E)-3-(3-Fluoro-4-methoxyphenyl)-1-(o-tolyl)prop-2-en-1-one (20)

3-Fluoro-4-methoxybenzaldehyde and 2′-methylacetophenone were used as reactants to give **20** (83% yield) as a faint yellow solid with a mp 69–70 °C; MS (ESI): *m/z* = 271.3 (M + H)^+^^48^.

##### (E)-1-(2,4-Dimethoxyphenyl)-3-(3-fluoro-4-methoxyphenyl)prop-2-en-1-one (21)

3-Fluoro-4-methoxybenzaldehyde and 2′,4′-dimethoxya­cetophenone were used as reactants to give **21** (86% yield) as a yellow solid with a mp 85–86 °C; MS (ESI): *m/z* = 317.3 (M + H)^+^^46^.

##### (E)-1-(2,3-Difluoro-4-methoxyphenyl)-4,4-dimethylpent-1-en-3-one (22)

2,3-Difluoro-4-methoxybenzaldehyde and pinacolone were used as reactants to give **22** (92% yield) as a white solid with a mp 128–129 °C; MS (ESI): *m/z* = 255.3 (M + H)^+^^48^.

##### (E)-1-(2,5-Difluoro-4-methoxyphenyl)-4,4-dimethylpent-1-en-3-one (23)

2,5-Difluoro-4-methoxybenzaldehyde and pinacolone were used as reactants to give **23** (92.8% yield) as a faint yellow solid with a mp 131–132 °C; MS (ESI): *m/z* = 255.4 (M + H)^+^^48^.

##### (E)-1-(5-Bromothiophen-2-yl)-4,4-dimethylpent-1-en-3-one (24)

5-Bromothiophene-2-carbaldehyde and pinacolone were used as reactants and the product was purified using CC (hexane/ethyl acetate 100:2) to give **24** (81% yield) as a yellow oil; ^1^H NMR (400 MHz, CDCl_3_) *δ* 7.65 (d, *J* = 15.3 Hz, 1H), 7.05–6.99 (m, 2H), 6.78 (d, *J* = 15.3 Hz, 1H), 1.19 (s, 9H); ^13^C NMR (101 MHz, CDCl_3_) *δ* 203.75, 142.11, 134.67, 131.89, 131.27, 120.10, 115.66, 43.26, 26.41; MS (ESI): *m/z* = 273.2 (M + H)^+^.

##### (E)-3-(5-Bromothiophen-2-yl)-1-(2-ethoxyphenyl)prop-2-en-1-one (25)

5-Bromothiophene-2-carbaldehyde and 2′-ethoxyacetophenone were used as reactants to give **25** (89% yield) as a yellow oil; MS (ESI): *m/z* = 337.2 (M + H)^+^^49^.

##### (E)-4,4-Dimethyl-1-(naphthalen-2-yl)pent-1-en-3-one (26)

2-Naphthaldehyde and pinacolone were used as reactants to give **26** (94% yield) as a yellow solid with a mp 117–119 °C; MS (ESI): *m/z* = 239.4 (M + H)^+^^49^.

##### (E)-4,4-Dimethyl-1-(quinolin-2-yl)pent-1-en-3-one (27)

Quinoline-2-carbaldehyde and pinacolone were used as reactants to give **27** (81% yield) as a pale yellow oil; MS (ESI): *m/z* = 240.3 (M + H)^+^^54^.

##### (E)-1-Phenyl-3-(quinolin-2-yl)prop-2-en-1-one (28)

Quinoline-2-carbaldehyde and acetophenone were used as reactants to give **28** (82% yield) as a pale yellow solid with a mp 118–120 °C; MS (ESI): *m/z* = 260.4 (M + H)^+^^54^.

##### (E)-1-([1,1′-Biphenyl]-4-yl)-4,4-dimethylpent-1-en-3-one (29)

[1,1′-Biphenyl]-4-carbaldehyde and pinacolone were used as reactants to give **29** (87% yield) as a yellow solid with a mp 128–130 °C; MS (ESI): *m/z* = 265.4 (M + H)^+^^49^.

##### (E)-3-([1,1′-Biphenyl]-4-yl)-1-(2-ethoxyphenyl)prop-2-en-1-one (30)

[1,1′-Biphenyl]-4-carbaldehyde and 2′-ethoxyacetophenone were used as reactants to give **30** (94% yield) as a yellow solid with a mp 98–99 °C; MS (ESI): *m/z* = 329.4 (M + H)^+^^49^.

##### (E)-1-Cyclopropyl-3-(4-nitrophenyl)prop-2-en-1-one (1a)

*p-*Nitrobenzaldehyde and acetylcyclopropane were used as reactants to give **1a** (80% yield) as a yellowish white solid with a mp 118.9–120 °C; MS (ESI): *m/z* = 218.3 (M + H)^+^^46^.

##### (E)-1-(1-Methylcyclopropyl)-3-(4-nitrophenyl)prop-2-en-1-one (2a)

*p*-Nitrobenzaldehyde and 1-(1-methylcyclopropyl)ethanone were used as reactants to give **2a** (92% yield) as a yellowish white solid with a mp 133.1 °C; MS (ESI): *m/z* = 232.3 (M + H)^+^^46^.

##### (E)-1-Cyclohexyl-3-(4-nitrophenyl)prop-2-en-1-one (3a)

*p-*Nitrobenzaldehyde and acetylcyclohexane were used as reactants to give **3a** (91% yield) as a beige solid with a mp 132–134 °C; MS (ESI): *m/z* = 260.4 (M + H)^+^^46^.

##### (E)-3-(4-Nitrophenyl)-1-(o-tolyl)prop-2-en-1-one (4a)

*p*-Nitrobenzaldehyde and 2′-methylacetophenone were used as reactants to give **4a** (87% yield) as a yellowish white solid with a mp 128–129 °C; MS (ESI): *m/z* = 268.3 (M + H)^+^^46^.

##### (E)-1-(2-Methoxyphenyl)-3-(4-nitrophenyl)prop-2-en-1-one (5a)

*p*-Nitrobenzaldehyde and 2′-methoxyacetophenone were used as reactants to give **5a** (79% yield) as a yellowish white solid with a mp 116.5–118 °C; MS (ESI): *m/z* = 284.4 (M + H)^+^^46^.

##### (E)-1-(3-Methoxyphenyl)-3-(4-nitrophenyl)prop-2-en-1-one (6a)

*p*-Nitrobenzaldehyde and 3′-methoxyacetophenone were used as reactants to give **6a** (91% yield) as a yellowish white solid with a mp 132.3–133.3 °C; MS (ESI): *m/z* = 284.3 (M + H)^+^^61^.

##### (E)-1-(4-Methoxyphenyl)-3-(4-nitrophenyl)prop-2-en-1-one (7a)

*p*-Nitrobenzaldehyde and 4′-methoxyacetophenone were used as reactants to give **7a** (81% yield) as a yellow solid with a mp 170–172 °C; MS (ESI): *m/z* = 284.3 (M + H)^+^^62^.

##### (E)-1-(3,4-Dimethoxyphenyl)-3-(4-nitrophenyl)prop-2-en-1-one (8a)

*p*-Nitrobenzaldehyde and 3′,4′-dimethoxyacetophenone were used as reactants to give **8a** (76% yield) as a yellow solid with a mp 189–191 °C; MS (ESI): *m/z* = 314.3 (M + H)^+^^63^.

##### (E)-1-(Benzo[d][1,3]dioxol-5-yl)-3-(4-nitrophenyl)prop-2-en-1-one (9a)

*p-*Nitrobenzaldehyde and 1-(benzo[*d*][1,3]dioxol-5-yl)ethanone were used as reactants to give **9a** (84% yield) as a dark yellow solid with a mp 182.4 °C; MS (ESI): *m/z* = 298.3 (M + H)^+^^64^.

##### (E)-3-(4-Chlorophenyl)-1-(naphthalen-2-yl)prop-2-en-1-one (10a)

*p*-Chlorobenzaldehyde and 2-acetylnaphthalene were used as reactants to give **10a** (81% yield) as a light yellow solid with a mp 162–164 °C; MS (ESI): *m/z* = 293.4 (M + H)^+^^65^.

##### (E)-1-([1,1′-Biphenyl]-4-yl)-3-(4-chlorophenyl)prop-2-en-1-one (11a)

*p*-Chlorobenzaldehyde and 1-([1,1′-biphenyl]-4-yl)ethan-1-one were used as reactants to give **11a** (92% yield) as a yellow solid with a mp 183–184 °C; MS (ESI): *m/z* = 319.3 (M + H)^+^^66^.

##### (E)-3-(4-Chlorophenyl)-1-(2-methoxyphenyl)prop-2-en-1-one (12a)

*p*-Chlorobenzaldehyde and 2′-methoxyacetophenone were used as reactants to give **12a** (86% yield) as a yellow solid with a mp 76–78 °C; MS (ESI): *m/z* = 273.3 (M + H)^+^^67^.

##### (E)-3-(4-Chlorophenyl)-1-(3-methoxyphenyl)prop-2-en-1-one (13a)

*p*-Chlorobenzaldehyde and 3′-methoxyacetophenone were used as reactants to give **13a** (90% yield) as a yellow solid with a mp 83 °C; MS (ESI): *m/z* = 273.3 (M + H)^+^^68^.

##### (E)-3-(4-Chlorophenyl)-1-(4-methoxyphenyl)prop-2-en-1-one (14a)

*p*-Chlorobenzaldehyde and 4′-methoxyacetophenone were used as reactants to give **14a** (89% yield) as a yellow solid with a mp 125–126 °C; MS (ESI): *m/z* = 273.3 (M + H)^+^^60^.

##### (E)-3-(4-Chlorophenyl)-1-(2,4-dimethoxyphenyl)prop-2-en-1-one (15a)

*p*-Chlorobenzaldehyde and 2′,4′-dimethoxyacetophenone were used as reactants to give **15a** (90% yield) as a yellow solid with a mp 123–125 °C; MS (ESI): *m/z* = 303.3 (M + H)^+^^60^.

##### (E)-3-(4-Chlorophenyl)-1-(3,4-dimethoxyphenyl)prop-2-en-1-one (16a)

*p*-Chlorobenzaldehyde and 3′,4′-dimethoxyacetophenone were used as reactants to give **16a** (92% yield) as a yellow solid with a mp 101–103 °C; MS (ESI): *m/z* = 303.3 (M + H)^+^^60^.

##### (E)-3-(2-Chlorophenyl)-1-(2-methoxyphenyl)prop-2-en-1-one (17a)

*o*-Chlorobenzaldehyde and 2′-methoxyacetophenone were used as reactants to give **17a** (89% yield) as a yellow solid with a mp 60–62 °C; MS (ESI): *m/z* = 273.3 (M + H)^+^^49^.

##### (E)-3-(2-Chlorophenyl)-1-(3-methoxyphenyl)prop-2-en-1-one (18a)

*o*-Chlorobenzaldehyde and 3′-methoxyacetophenone were used as reactants to give **18a** (88% yield) as a yellow solid with a mp 117 °C; MS (ESI): *m/z* = 273.3 (M + H)^+^^49^.

##### (E)-3-(2-Chlorophenyl)-1-(4-methoxyphenyl)prop-2-en-1-one (19a)

*o*-Chlorobenzaldehyde and 4′-methoxyacetophenone were used as reactants to give **19a** (95% yield) as a yellow solid with a mp 79–80 °C; MS (ESI): *m/z* = 273.3 (M + H)^+^^49^.

##### (E)-3-(2-Chlorophenyl)-1-(3,4-dimethoxyphenyl)prop-2-en-1-one (20a)

*o*-Chlorobenzaldehyde and 3′,4′-dimethoxyacetophenone were used as reactants to give **20a** (62% yield) as a yellow solid with a mp 115.4–117.1 °C; MS (ESI): *m/z* = 303.3 (M + H)^+^^69^.

##### (E)-3-(3-Chlorophenyl)-1-(2-methoxyphenyl)prop-2-en-1-one (21a)

*m*-Chlorobenzaldehyde and 2′-methoxyacetophenone were used as reactants to give **21a** (81% yield) as a yellow solid with a mp 96–98 °C; MS (ESI): *m/z* = 273.3 (M + H)^+^^49^.

##### (E)-3-(3-Chlorophenyl)-1-(4-methoxyphenyl)prop-2-en-1-one (22a)

*m*-Chlorobenzaldehyde and 4′-methoxyacetophenone were used as reactants to give **22a** (89% yield) as a yellow solid with a mp 97–99 °C; MS (ESI): *m/z* = 273.3 (M + H)^+^^49^.

##### (E)-1-(4-Chlorophenyl)-3-(3,5-difluorophenyl)prop-2-en-1-one (23a)

3,5-Difluorobenzaldehyde and 4′-chloroacetophenone were used as reactants to give **23a** (86% yield) as a white solid with a mp 123.6–124 °C; MS (ESI): *m/z* = 279.3 (M + H)^+^^70^.

##### (E)-3-(3,5-Difluorophenyl)-1-(3-methoxyphenyl)prop-2-en-1-one (24a)

3,5-Difluorobenzaldehyde and 3′-methoxyacetophenone were used as reactants to give **24a** (90% yield) as a yellowish white solid with a mp 95.2–97.2 °C; MS (ESI): *m/z* = 275.3 (M + H)^+^^71^.

##### (E)-3-(3,5-Difluorophenyl)-1-(4-methoxyphenyl)prop-2-en-1-one (25a)

3,5-Difluorobenzaldehyde and 4′-methoxyacetophenone were used as reactants to give **25a** (92% yield) as a yellow solid with a mp 154.3–155 °C; MS (ESI): *m/z* = 275.3 (M + H)^+^^70^.

##### (E)-3-(3,5-Difluorophenyl)-1-(3,4-dimethoxyphenyl)prop-2-en-1-one (26a)

3,5-Difluorobenzaldehyde and 3′,4′-dimethoxyacetophenone were used as reactants to give **26a** (87% yield) as a yellow solid with a mp 132.8–134.8 °C; MS (ESI): *m/z* = 305.3 (M + H)^+^^71^.

### Biological evaluation

#### Human neutrophil isolation

The study involving neutrophils was approved by the Institutional Review Board of Chang Gung Memorial Hospital under IRB number: 201902217A3 and was conducted in accordance with the principles of the Declaration of Helsinki. Healthy volunteers aged 20–35 years provided signed informed consents for blood collection. Isolation of neutrophils was done using dextran sedimentation, Ficoll-Hypaque density gradient centrifugation, and hypotonic haemolysis^72^. For the anti-inflammatory studies, neutrophils were suspended in Hank’s balanced salt solution (HBSS) containing 1 mM Ca^2+^ at a pH of 7.4^72^.

#### Measurement of superoxide anion release

The reduction of ferricytochrome *c* was utilised to measure the production of SO anions in activated human neutrophils. Initially, ferricytochrome *c* (0.6 mg/mL) was incubated, followed by treating neutrophils (6 × 10^5^ cells/mL) with DMSO (0.1%, as control) or experimental compounds at 37 °C for 5 min. The neutrophils were then stimulated with fMLF (0.1 μM) and CB (1 μg/mL) for 13 min. Absorbance alterations at 550 nm were recorded using a Hitachi U-3010 spectrophotometer (Tokyo, Japan). LY294002 was used as a positive control^73^.

#### Determination of elastase release

The assay began with the incubation of the elastase substrate, methoxysuccinyl-Ala-Ala-Pro-Val-p-nitroanilide (100 μM), followed by treating neutrophils (6 × 10^5^/mL) with DMSO (0.1%, as control) or experimental compounds at 37 °C for 5 min. Neutrophils were subsequently stimulated with fMLF (0.1 μM) and CB (0.5 μg/mL) for 13 min. Absorbance alterations at 405 nm were recorded using a Hitachi U-3010 spectrophotometer (Tokyo, Japan). LY294002 was used as a positive control^73^.

#### Cytotoxicity assay

Cell viability was assessed using a commercial LDH assay kit (Promega, Madison, WI). Human neutrophils (6 × 10^5^ cells/mL) were subjected to DMSO (0.1%) or experimental compounds for 15 min at 37 °C. The cells were then lysed with 0.1% Triton X-100 at the same temperature, and the total release of LDH was measured. After centrifuging the supernatant, it was treated with LDH assay reagent for 30 min. Alterations in absorbance at 490 nm were recorded using a Hitachi U-3010 spectrophotometer (Tokyo, Japan).

#### Immunoblotting assay

Human neutrophils (2.5 × 10^7^ cells/mL) were first subjected to DMSO (0.1%, serving as control) or experimental compounds at 37 °C for 5 min. Following this, cells were stimulated with fMLF (0.1 μM) for 30 s. Next, cells were lysed in a boiled sample buffer (pH 6.8, consisting of 62.5 mM Tris–HCl, 4% SDS, 5% 2-mercaptoethanol, 0.0125% bromophenol blue, 8.75% glycerol, 1 mM phenylmethylsulfonyl fluoride, 1% protease inhibitor cocktail (P8340, Sigma, St. Louis, MO), and 1% phosphatase inhibitor cocktail 3 (P0044, Sigma, St. Louis, MO) at 100 °C for 15 min. The lysates were then run on a 12% SDS-polyacrylamide gel and transferred to nitrocellulose membranes (Whatman; Perkin-Elmer Life Science, Waltham, MA). The proteins were probed using specific primary antibodies and peroxidase-conjugated secondary anti-rabbit antibodies (Cell Signaling Technology, Danvers, MA), with chemiluminescence detection and quantification performed using the ChemiDoc MP Imaging System and ImageLab software (BioRad, Hercules, CA). Primary antibodies used included those for p38 (1:8000), phospho-p38 (1:3000), JNK (1:3000), phospho-JNK (1:3000), Akt (1:5000), phospho-Akt (Ser-473) (1:2000), ERK (1:8000), phospho-ERK (1:8000), and HRP-linked anti-rabbit IgG, to measure total and phosphorylated forms of MAPK and Akt proteins^56^. The quantitative measurements for all samples were adjusted to correspond with the levels of the total protein present.

#### Statistics

Statistical analysis was conducted using SigmaPlot (version 8.0, Systat Software, San Jose, CA). Results are expressed as the mean ± standard error of the mean (SEM). Student’s *t*-test was used to determine statistical significance, with a *p* value of less than 0.05 considered significant.

## Supplementary Material

Original image for Figure 6 Part A_CX12 is 26a.jpg

Supplementary Information.pdf

Original image for Figure 5 Part B_CX2 is 6a.jpg

Original image for Figure 6 Part C_CX12 is 26a.jpg

Original image for Figure 5 Part A_CX2 is 6a.jpg

Original image for Figure 5 Part D_CX2 is 6a.jpg

Original image for Figure 5 Part C_CX2 is 6a.jpg

Original image for Figure 6 Part B_CX12 is 26a.jpg

Original image for Figure 6 Part D_CX12 is 26a.jpg

## Data Availability

The data that support the findings of this study are available from the corresponding authors (MA-H and T-L H), upon reasonable request.
