# Supplementary figures and images for: Discovery of 1,3-disubstituted prop-2-en-1-one derivatives as inhibitors of neutrophilic inflammation via modulation of MAPK and Akt pathways
Source: J Enzyme Inhib Med Chem. 2024 Sep 19;39(1):2402988. doi: 10.1080/14756366.2024.2402988 (PMC11413964; doi:10.1080/14756366.2024.2402988)

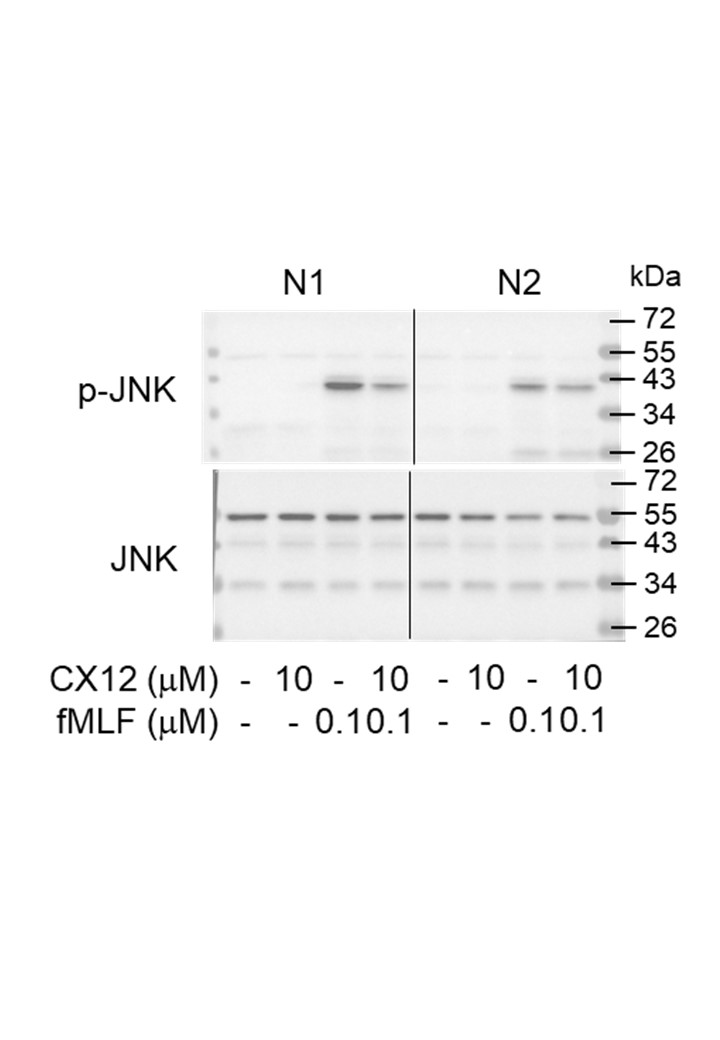

Supplement: Original image for Figure 6 Part A_CX12 is 26a.jpg [file IENZ_A_2402988_SM2733.jpg]

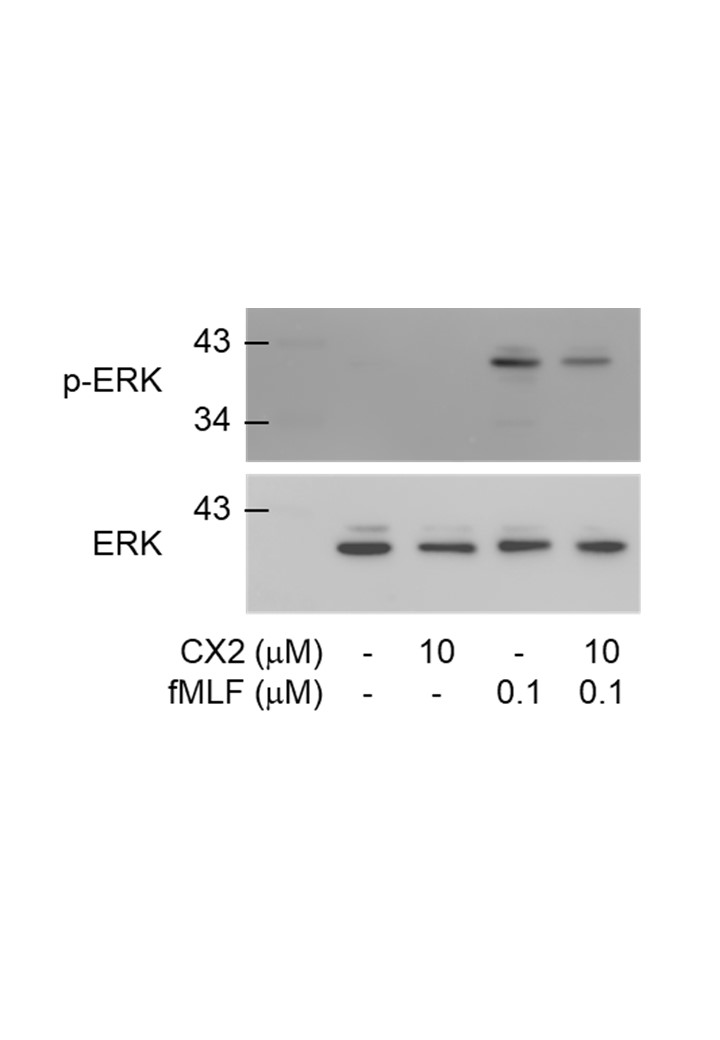

Supplement: Original image for Figure 5 Part B_CX2 is 6a.jpg [file IENZ_A_2402988_SM2731.jpg]

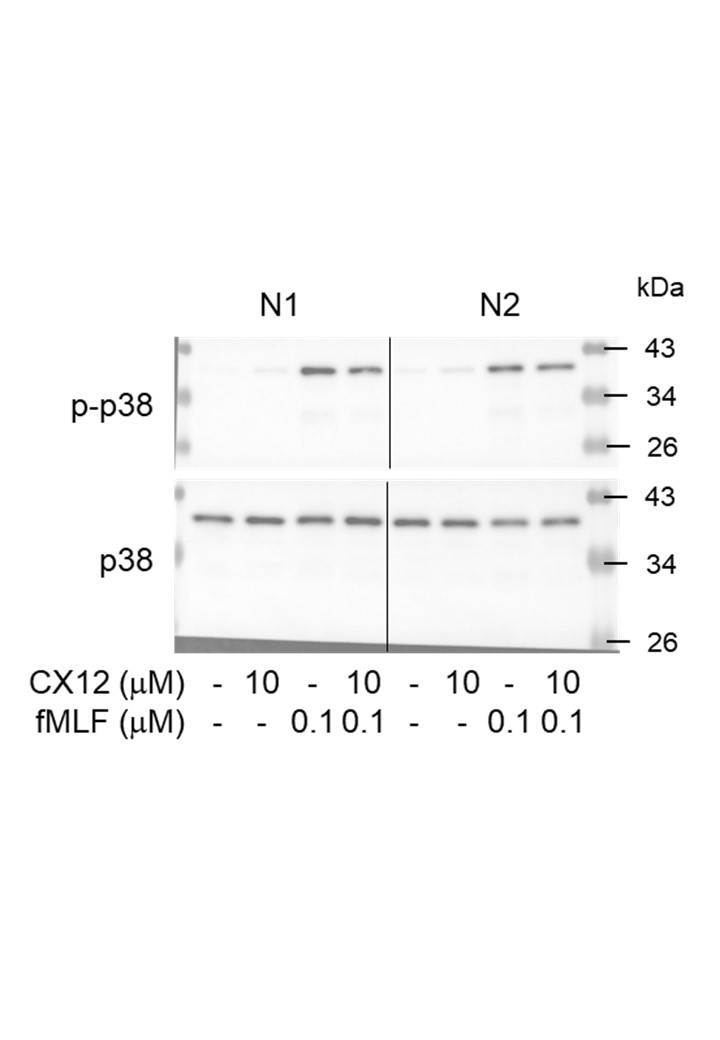

Supplement: Original image for Figure 6 Part C_CX12 is 26a.jpg [file IENZ_A_2402988_SM2730.jpg]

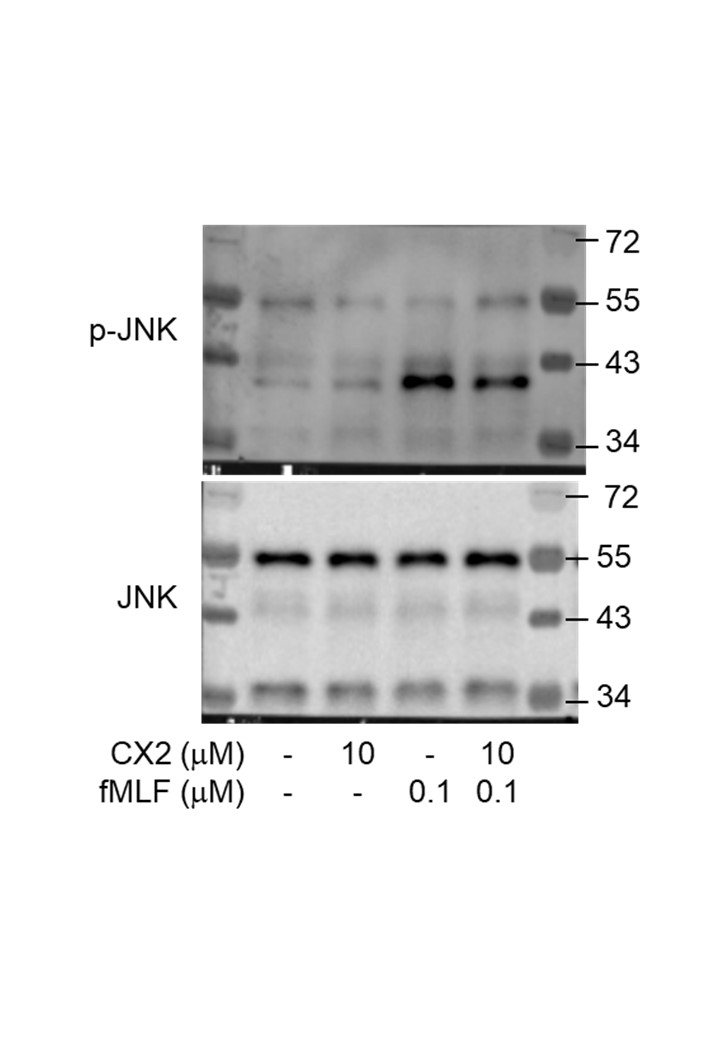

Supplement: Original image for Figure 5 Part A_CX2 is 6a.jpg [file IENZ_A_2402988_SM2729.jpg]

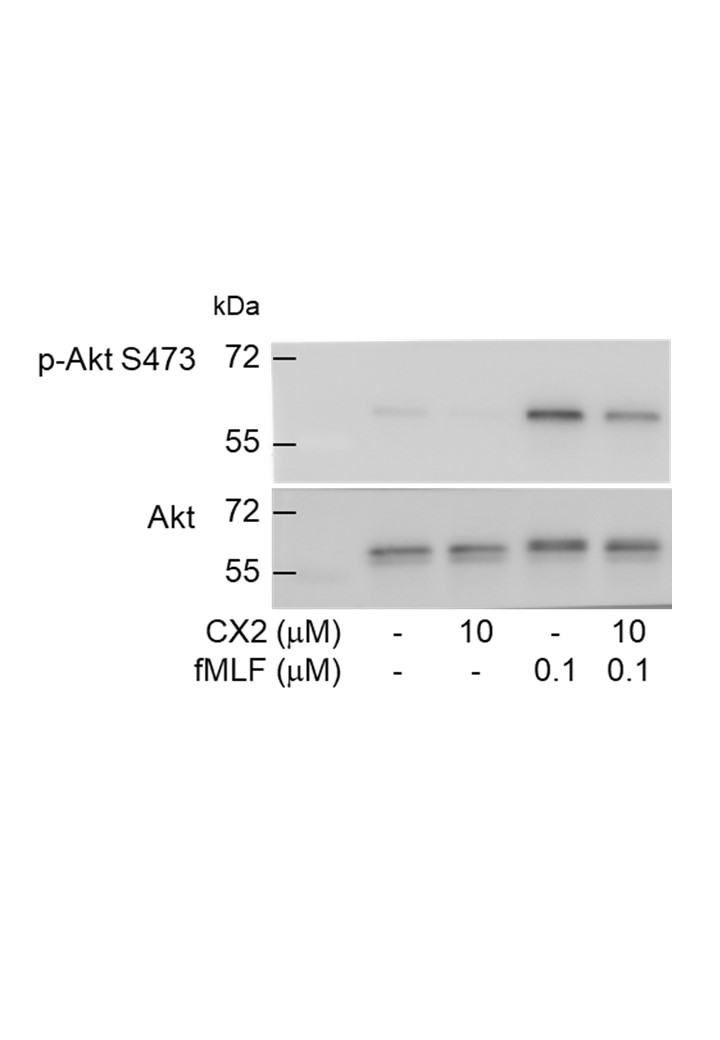

Supplement: Original image for Figure 5 Part D_CX2 is 6a.jpg [file IENZ_A_2402988_SM2728.jpg]

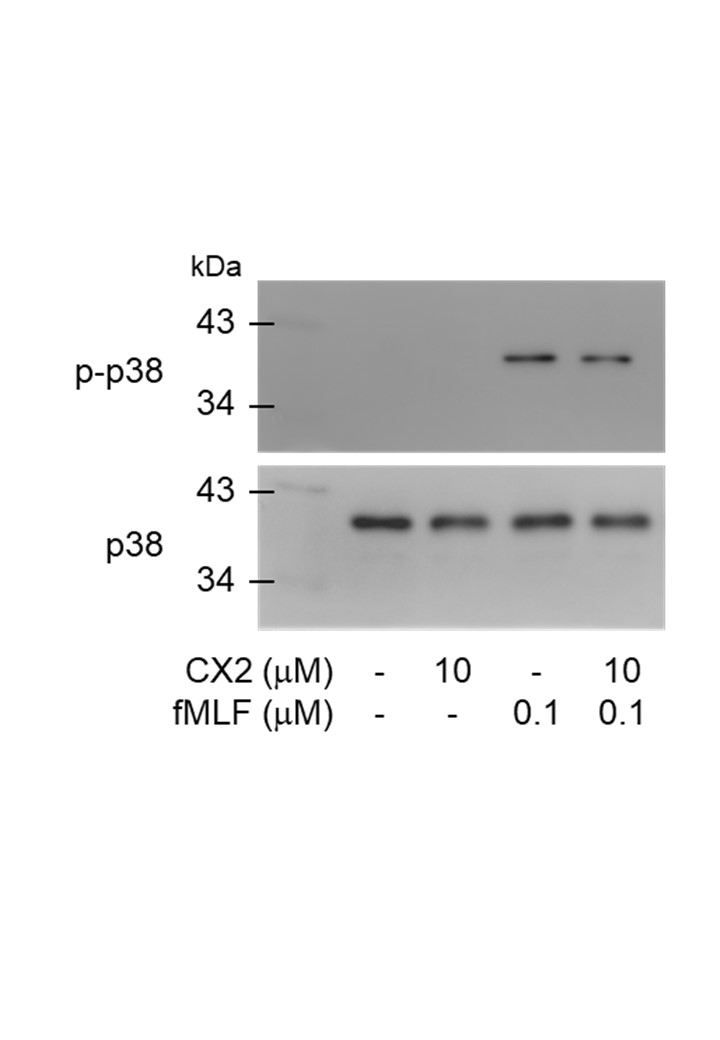

Supplement: Original image for Figure 5 Part C_CX2 is 6a.jpg [file IENZ_A_2402988_SM2727.jpg]

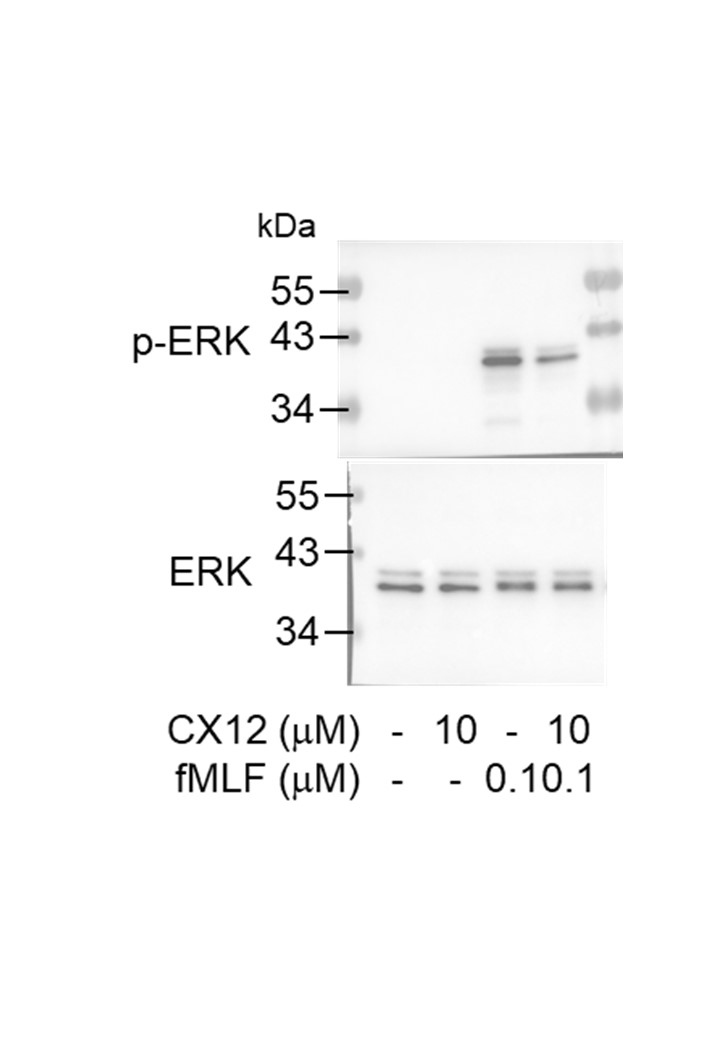

Supplement: Original image for Figure 6 Part B_CX12 is 26a.jpg [file IENZ_A_2402988_SM2726.jpg]

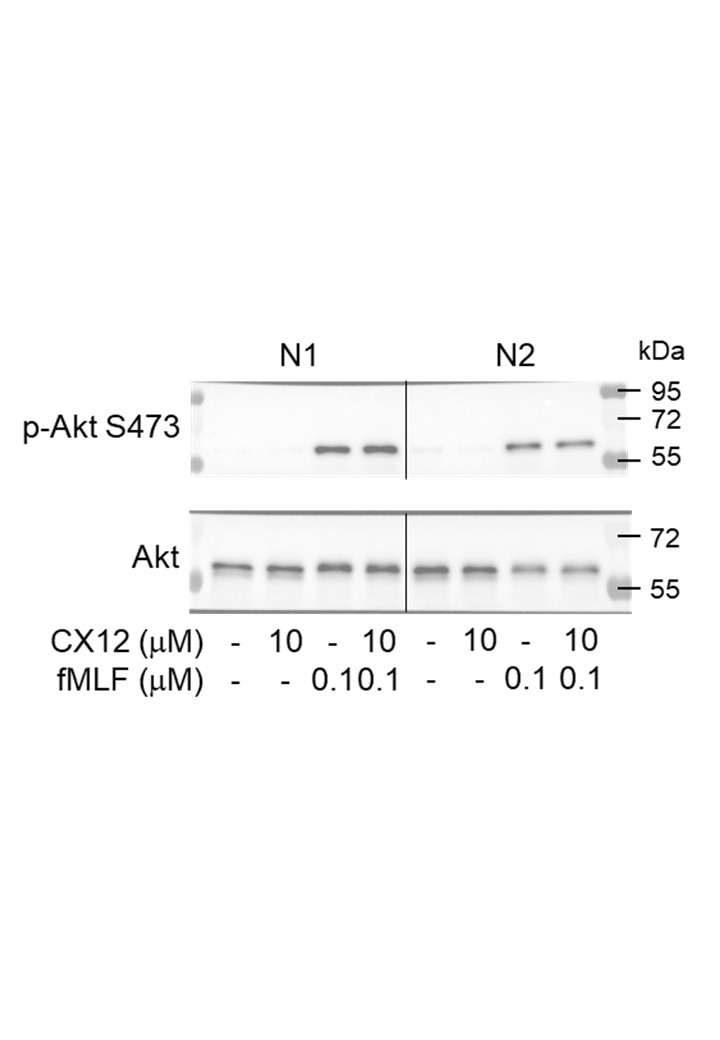

Supplement: Original image for Figure 6 Part D_CX12 is 26a.jpg [file IENZ_A_2402988_SM2725.jpg]
