# Supplementary material for: Discovery of 1,3-disubstituted prop-2-en-1-one derivatives as inhibitors of neutrophilic inflammation via modulation of MAPK and Akt pathways
Source: J Enzyme Inhib Med Chem. 2024 Sep 19;39(1):2402988. doi: 10.1080/14756366.2024.2402988 (PMC11413964; doi:10.1080/14756366.2024.2402988)
Supplement: Supplementary Information.pdf [file IENZ_A_2402988_SM2732.pdf]

Supplementary Information

Discovery of 1,3-Disubstituted Prop-2-en-1-one Derivatives as Inhibitors of Neutrophilic Inflammation via Modulation of MAPK and Akt Pathways

Mohammad Abdel-Halim [1,*], Dalia S. El-Gamil [2], Mennatallah A. Hammam [3], Mohamed El-Shazly [4], Yi-Hsuan Wang [5], Po-Hsiung Kung [5,6], Yu-Cheng Chen [6], Michal Korinek [7], Ashraf H. Abadi [1], Matthias Engel [8], Tsong-Long Hwang [5,6,9, 10,*]

[1] Department of Pharmaceutical Chemistry, Faculty of Pharmacy and Biotechnology, German University in Cairo, Cairo 11835, Egypt

[2] Department of Chemistry, Faculty of Pharmacy, Ahram Canadian University, Cairo, 12451, Egypt

[3] School of Life and Medical Sciences, University of Hertfordshire Hosted by Global Academic Foundation, New Administrative Capital, Cairo 11865, Egypt

[4] Department of Pharmacognosy, Faculty of Pharmacy, Ain-Shams University, Cairo, 11566, Egypt

[5] Graduate Institute of Natural Products, College of Medicine, Chang Gung University, Taoyuan 333, Taiwan

[6] Research Center for Chinese Herbal Medicine, Graduate Institute of Healthy Industry Technology, College of Human Ecology, Chang Gung University of Science and Technology, Taoyuan 333, Taiwan

[7] Graduate Institute of Natural Products, College of Pharmacy, Kaohsiung Medical University, Kaohsiung 807, Taiwan

[8] Pharmaceutical and Medicinal Chemistry, Saarland University, Campus C2.3, D-66123 Saarbrücken, Germany

[9] Department of Anesthesiology, Chang Gung Memorial Hospital, Taoyuan 333, Taiwan

[10] Department of Chemical Engineering, Ming Chi University of Technology, New Taipei City 243, Taiwan

*Corresponding authors

Mohammad Abdel-Halim (mohammad.abdel-halim@guc.edu.eg) and Tsong-Long Hwang (htl@mail.cgu.edu.tw)

Table of Contents

Figure S1. Full gels for Western blot images used for quantification in Figure 5………………………. 2

Figure S2. Full gels for Western blot images used for quantification in Figure 6……………..………3

Mass spectra for compounds (1-30) and (1a-26a)……………………..…………………………..4

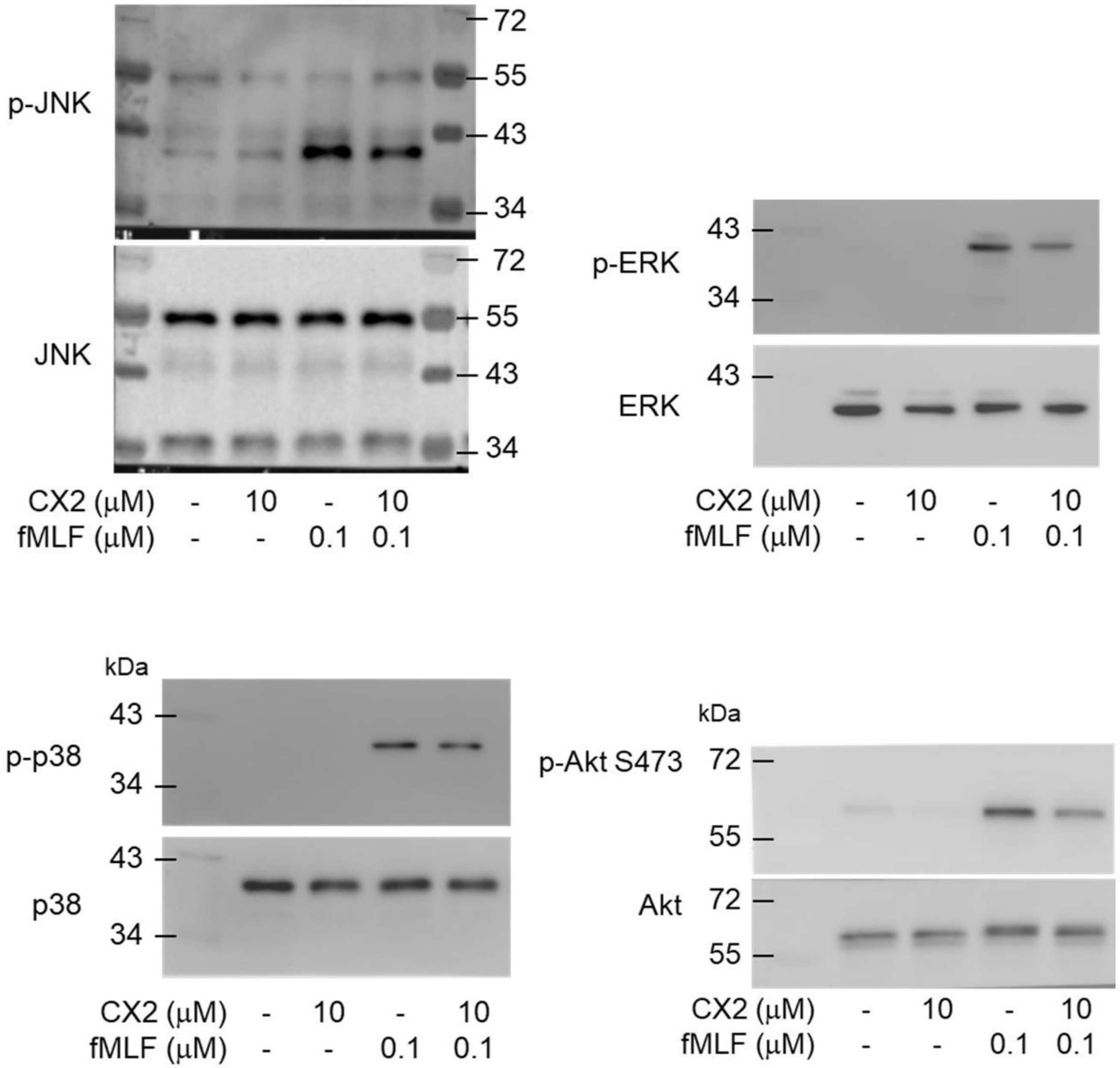

Figure S1. Full gels for Western blot images used for quantification in Figure 5. CX2 is compound **6a**.

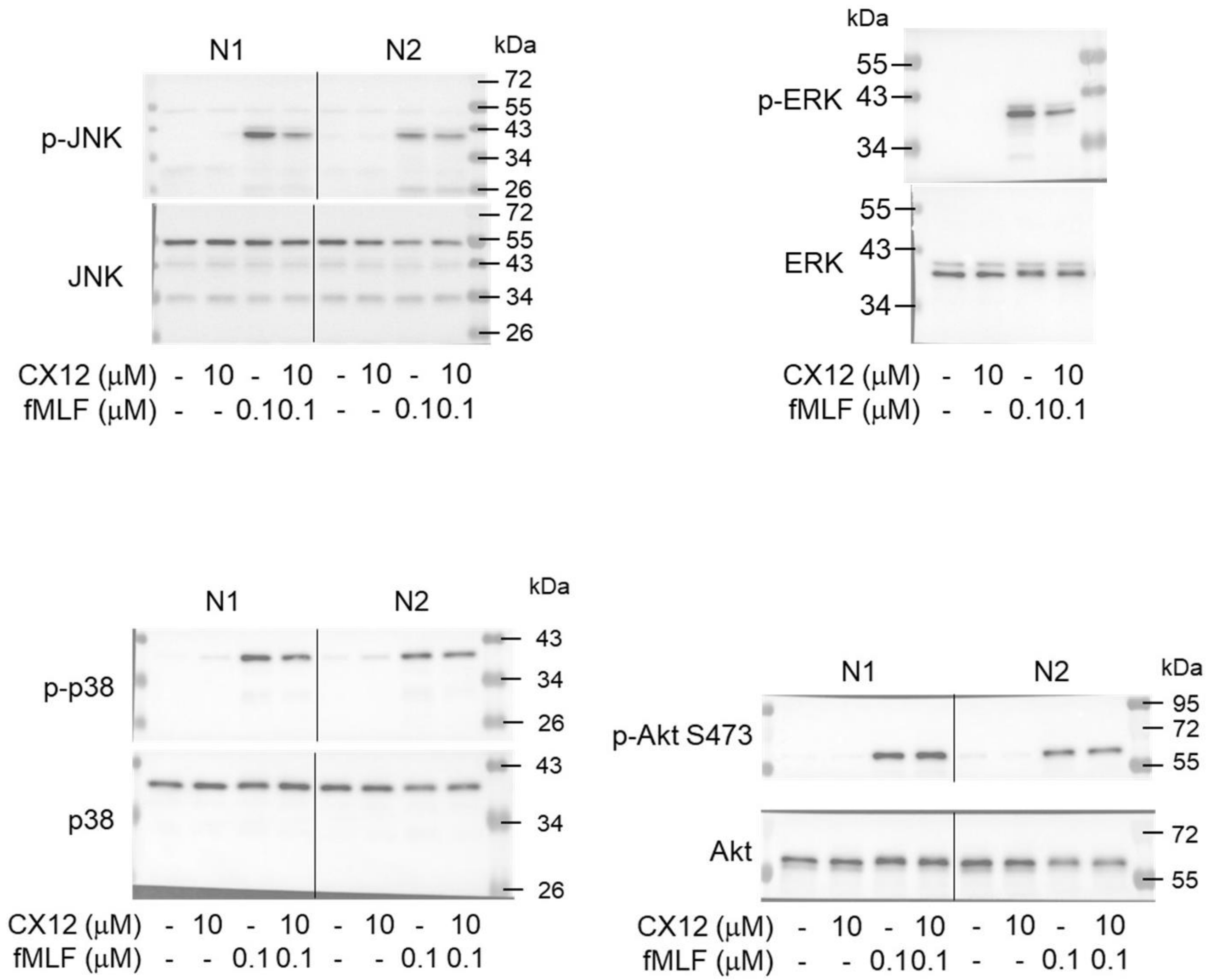

Figure S2. Full gels for Western blot images used for quantification in Figure 6. CX12 is compound **26a**.

Mass spectra for compounds (1-30) and (1a-26a)

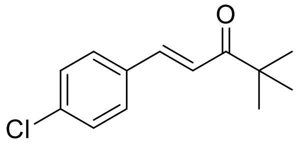

1

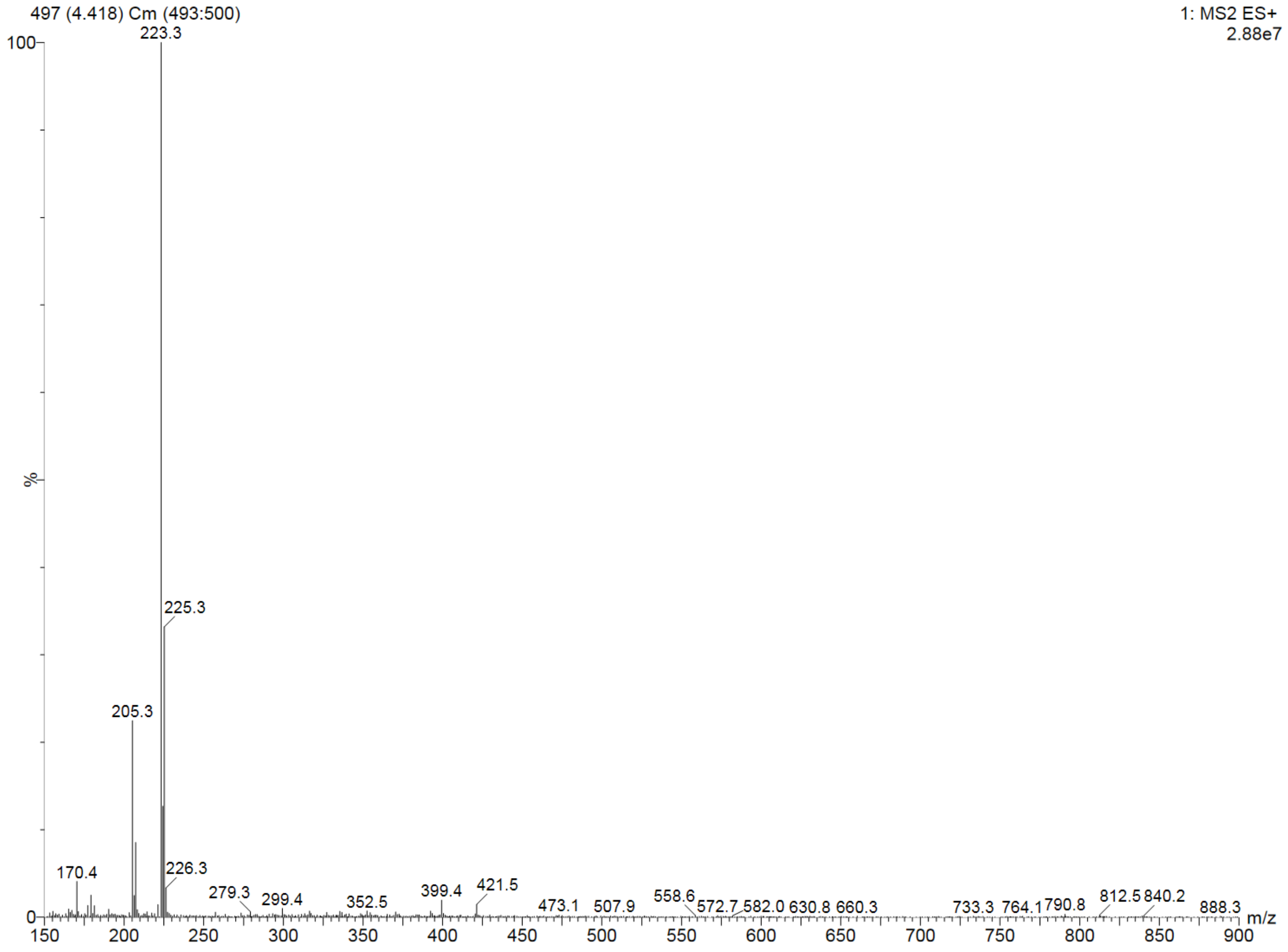

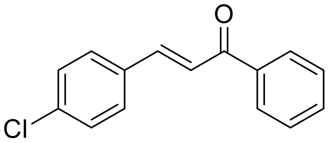

2

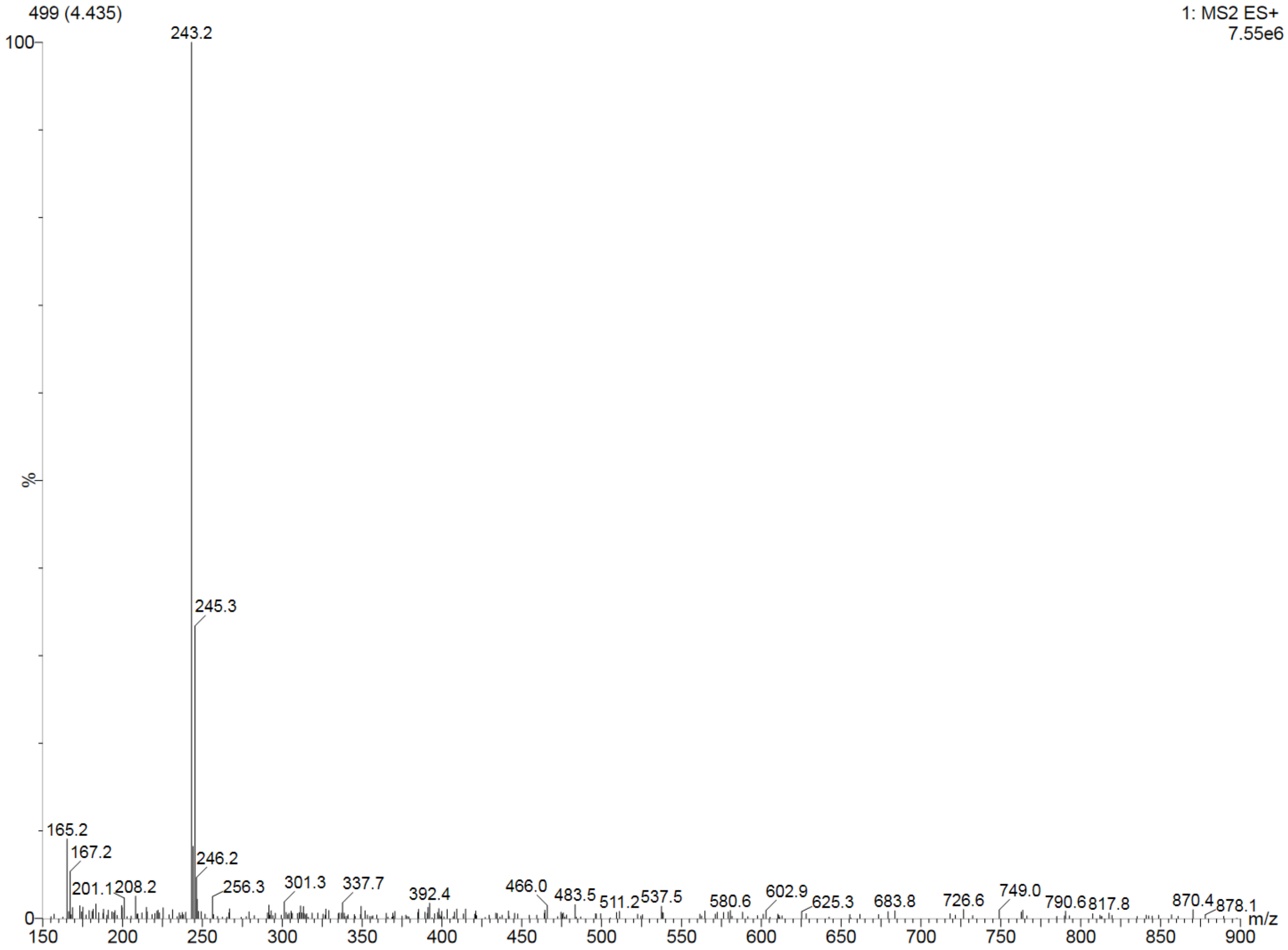

499 (4.435)
1: MS2 ES+
7.55e6
100
%
0
243.2
245.3
246.2
165.2
167.2
201.1
208.2
256.3
301.3
337.7
392.4
466.0
483.5
511.2
537.5
580.6
602.9
625.3
683.8
726.6
749.0
790.6
817.8
870.4
878.1
m/z
150
200
250
300
350
400
450
500
550
600
650
700
750
800
850
900

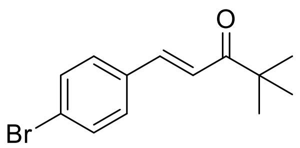
O
Br

3

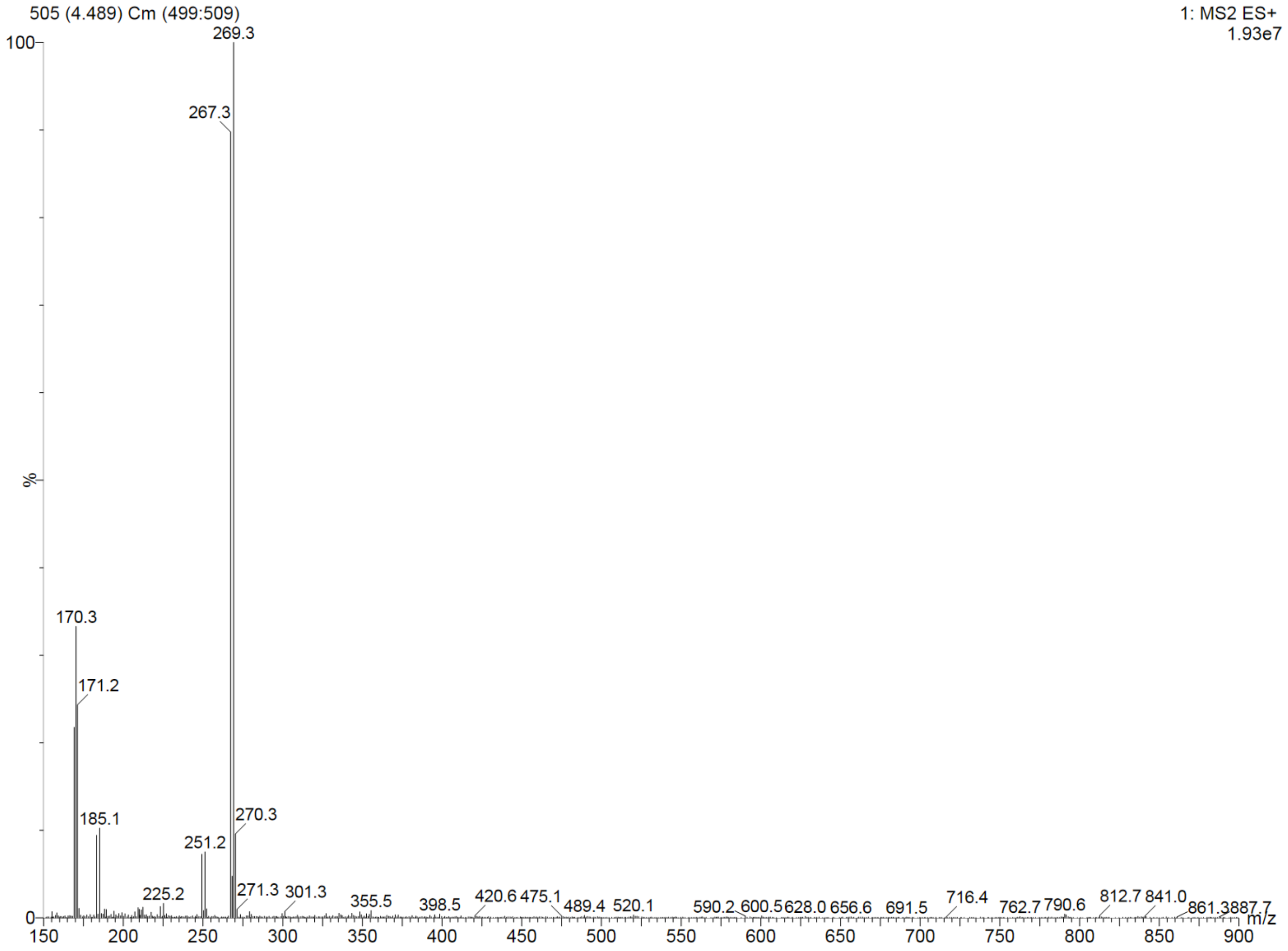
505 (4.489) Cm (499:509)
1: MS2 ES+
1.93e7
269.3
267.3
170.3
171.2
185.1
251.2
270.3
225.2
271.3
301.3
355.5
398.5
420.6
475.1
489.4
520.1
590.2
600.5
628.0
656.6
691.5
716.4
762.7
790.6
812.7
841.0
861.3
887.7
%
100
0
m/z
150
200
250
300
350
400
450
500
550
600
650
700
750
800
850
900

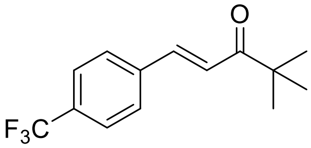
O
F3C

4

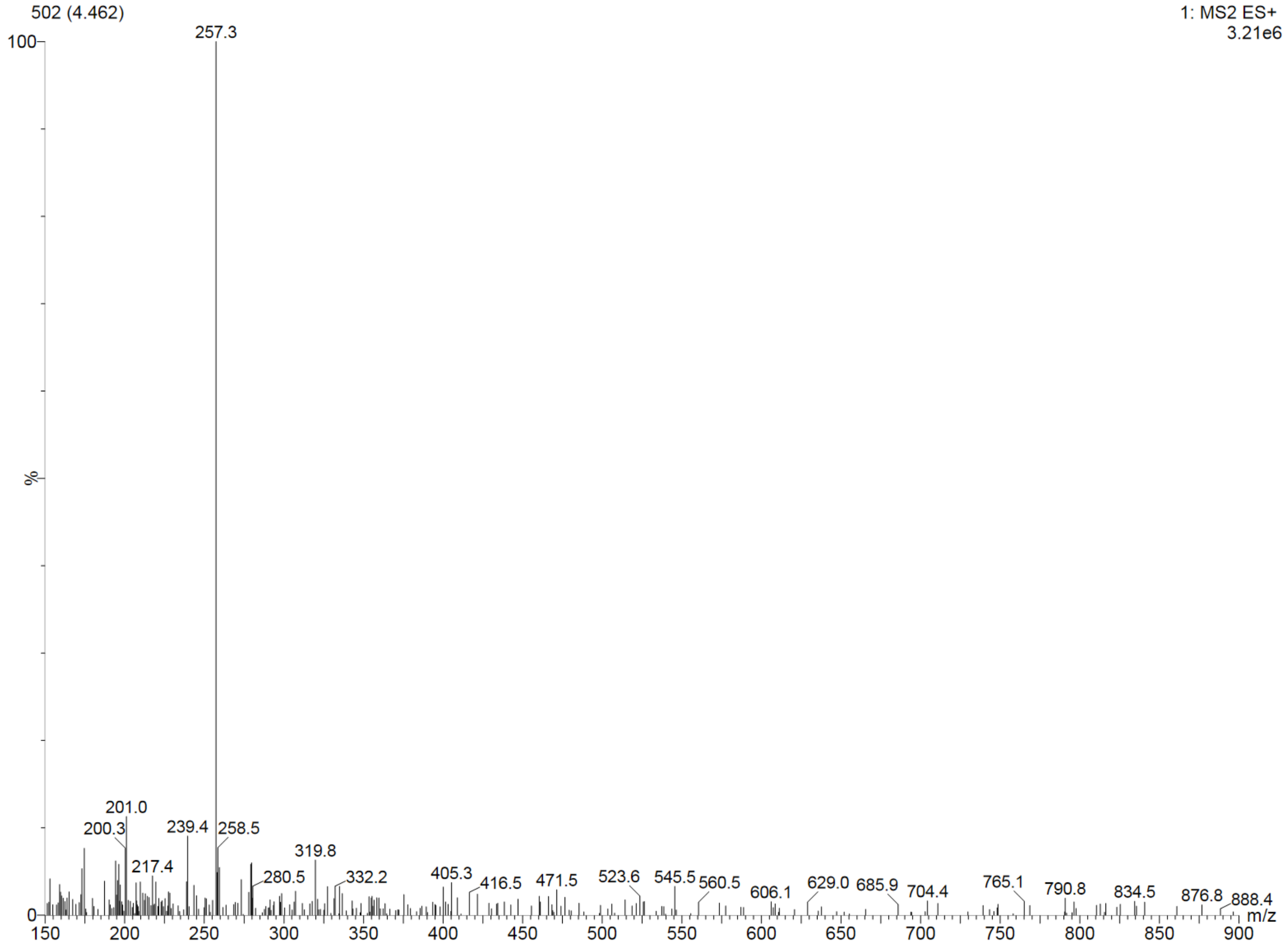
502 (4.462)
1: MS2 ES+
3.21e6
100
%
0
257.3
201.0
200.3
239.4
258.5
217.4
319.8
280.5
332.2
405.3
416.5
471.5
523.6
545.5
560.5
606.1
629.0
685.9
704.4
765.1
790.8
834.5
876.8
888.4
m/z
150
200
250
300
350
400
450
500
550
600
650
700
750
800
850
900

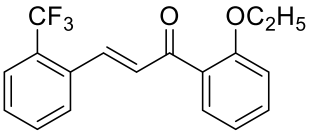
CF3
O
OC2H5

5

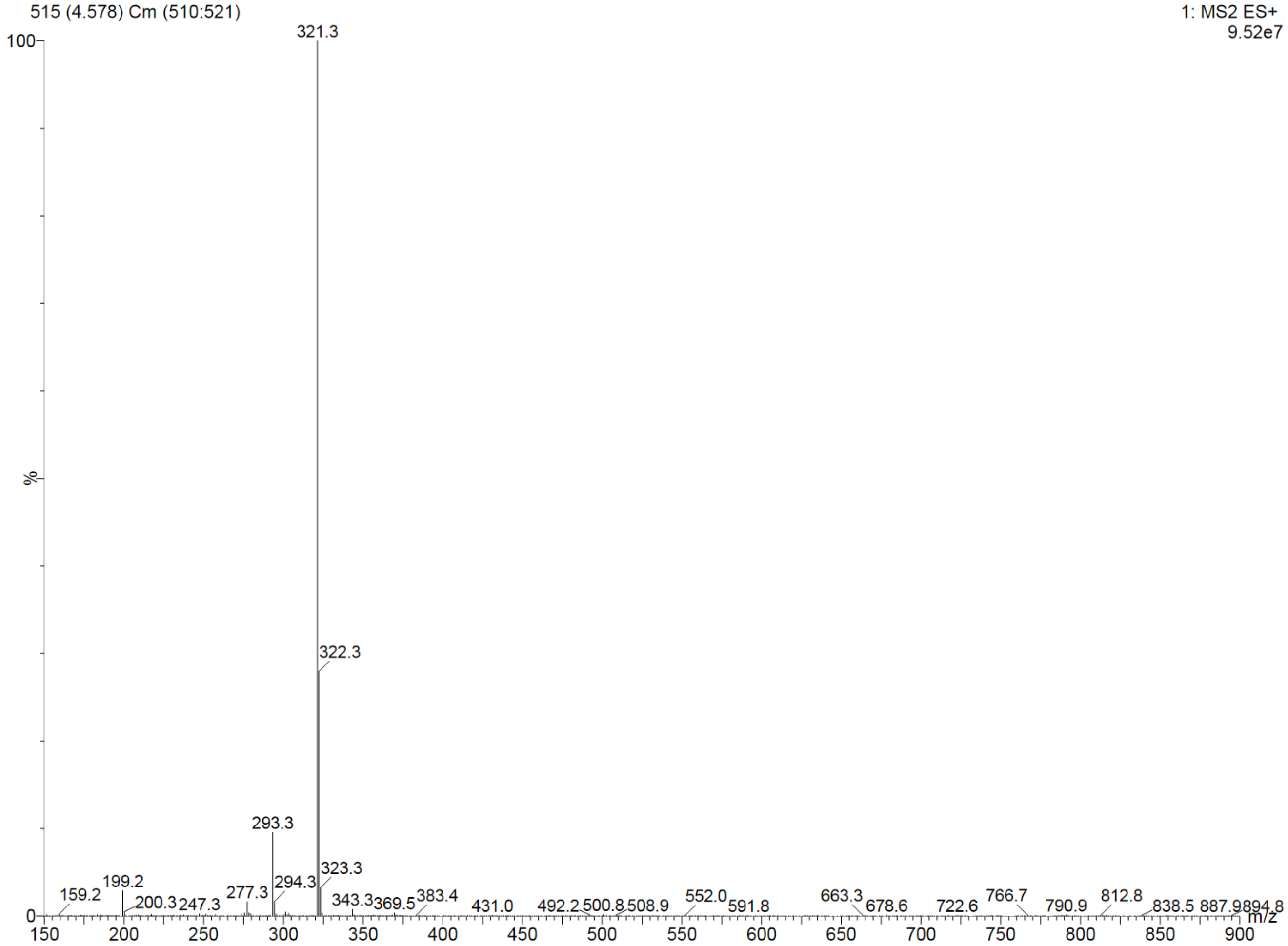
515 (4.578) Cm (510:521)
1: MS2 ES+
9.52e7
100
%
0
321.3
322.3
293.3
323.3
199.2
159.2
200.3
247.3
277.3
294.3
343.3
369.5
383.4
431.0
492.2
500.8
508.9
552.0
591.8
663.3
678.6
722.6
766.7
790.9
812.8
838.5
887.9
894.8
m/z
150
200
250
300
350
400
450
500
550
600
650
700
750
800
850
900

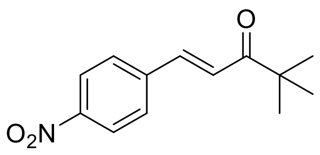
O
O_2N

6

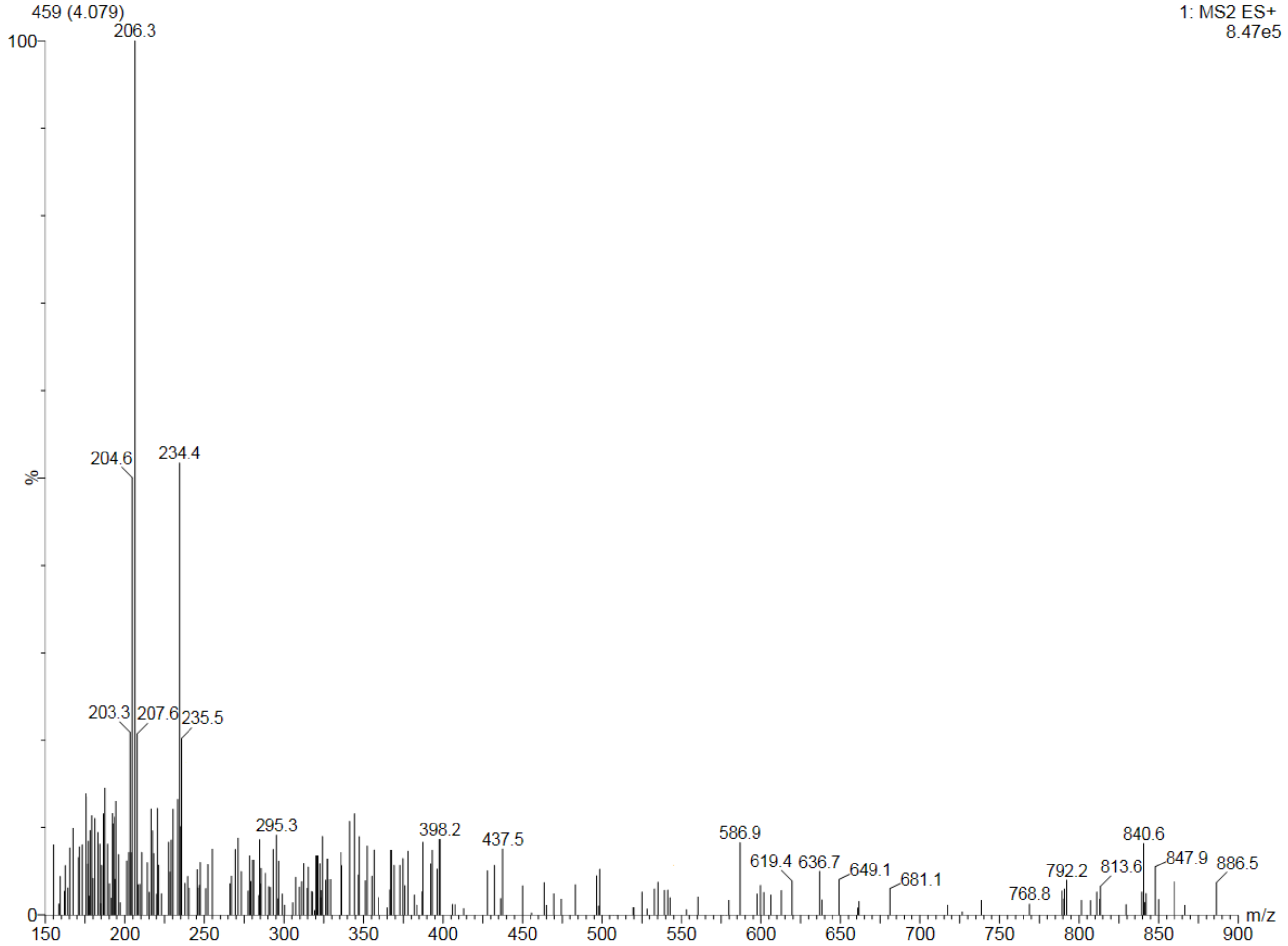
459 (4.079)
1: MS2 ES+
8.47e5
100
%
0
206.3
204.6
234.4
203.3
207.6
235.5
295.3
398.2
437.5
586.9
619.4
636.7
649.1
681.1
768.8
792.2
813.6
840.6
847.9
886.5
m/z
150
200
250
300
350
400
450
500
550
600
650
700
750
800
850
900

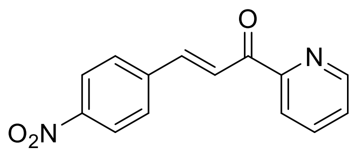
O
N
O_2N

7

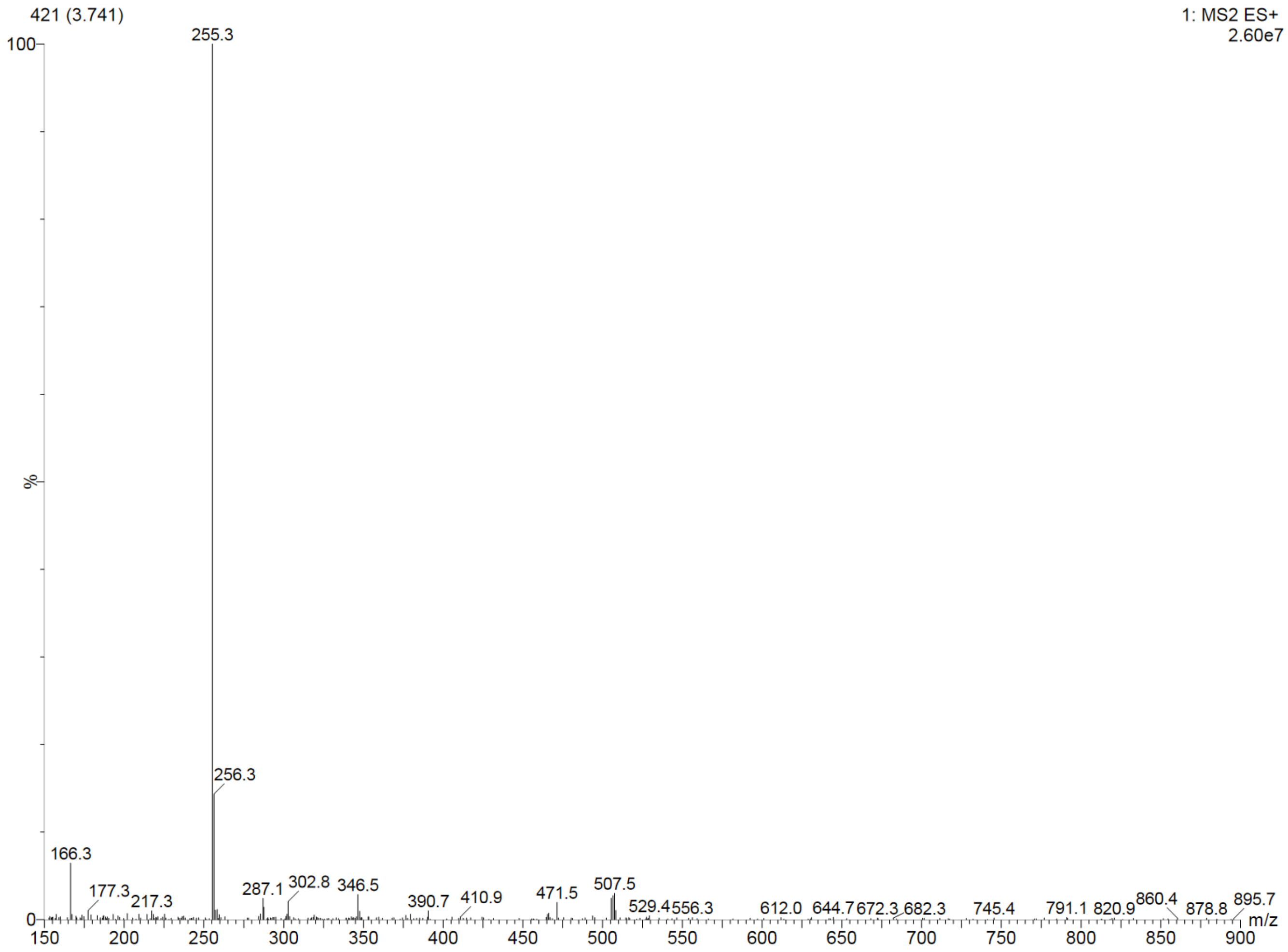
421 (3.741)
1: MS2 ES+
2.60e7
100
%
0
255.3
256.3
166.3
177.3
217.3
287.1
302.8
346.5
390.7
410.9
471.5
507.5
529.4
556.3
612.0
644.7
672.3
682.3
745.4
791.1
820.9
860.4
878.8
895.7
m/z
150
200
250
300
350
400
450
500
550
600
650
700
750
800
850
900

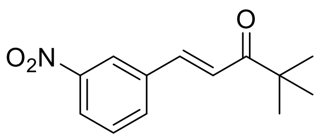
O2N
O

8

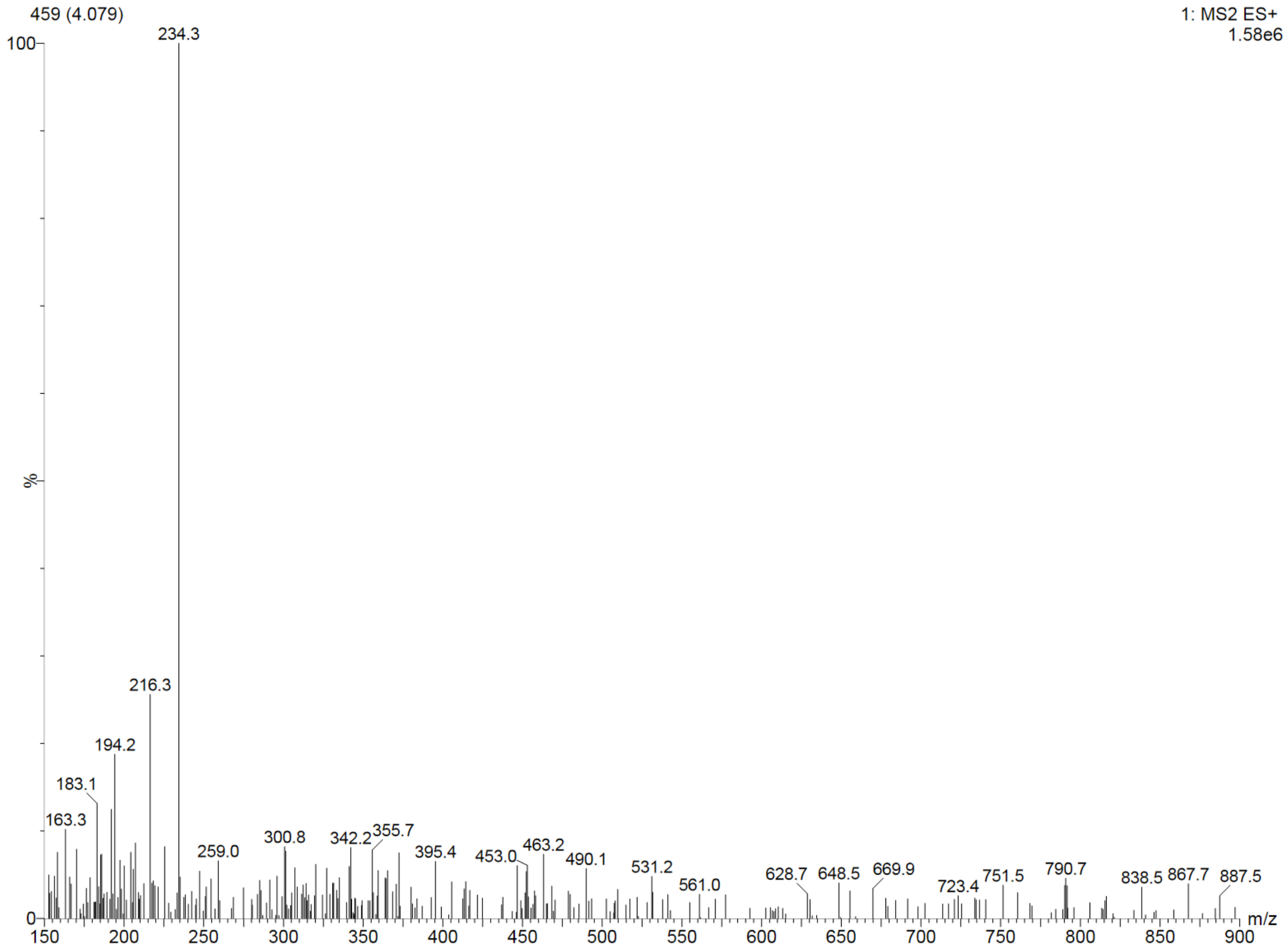
459 (4.079)
1: MS2 ES+
1.58e6
100
%
0
234.3
216.3
194.2
183.1
163.3
259.0
300.8
342.2
355.7
395.4
453.0
463.2
490.1
531.2
561.0
628.7
648.5
669.9
723.4
751.5
790.7
838.5
867.7
887.5
m/z
150
200
250
300
350
400
450
500
550
600
650
700
750
800
850
900

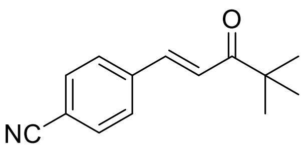
O
NC

9

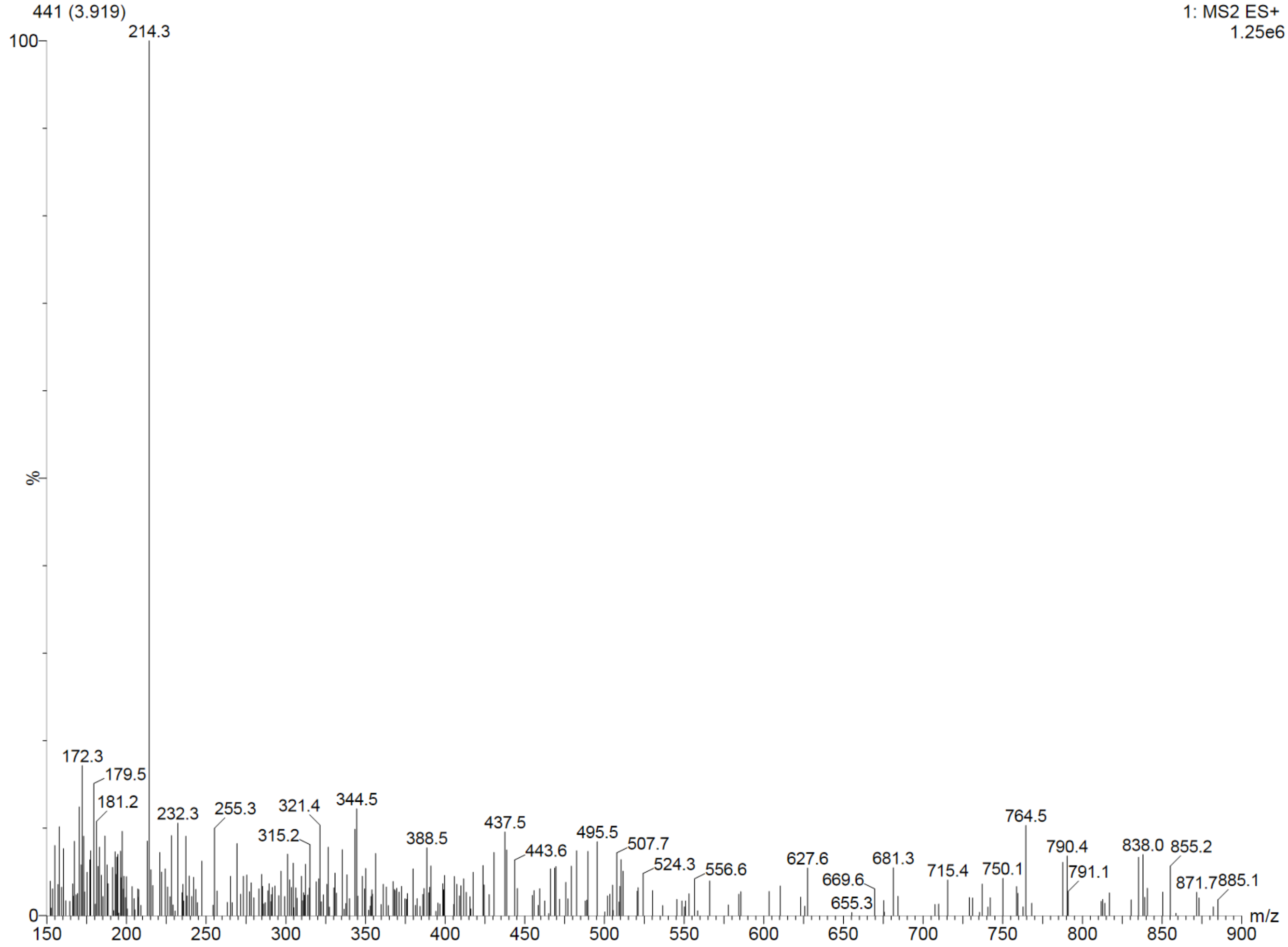
441 (3.919)
1: MS2 ES+
1.25e6
214.3
172.3
179.5
181.2
232.3
255.3
321.4
344.5
315.2
388.5
437.5
443.6
495.5
507.7
524.3
556.6
627.6
669.6
655.3
681.3
715.4
750.1
764.5
790.4
791.1
838.0
855.2
871.7
885.1
%
m/z

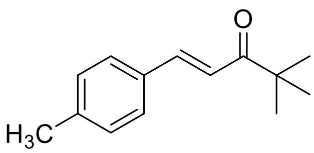
O
H3C

10

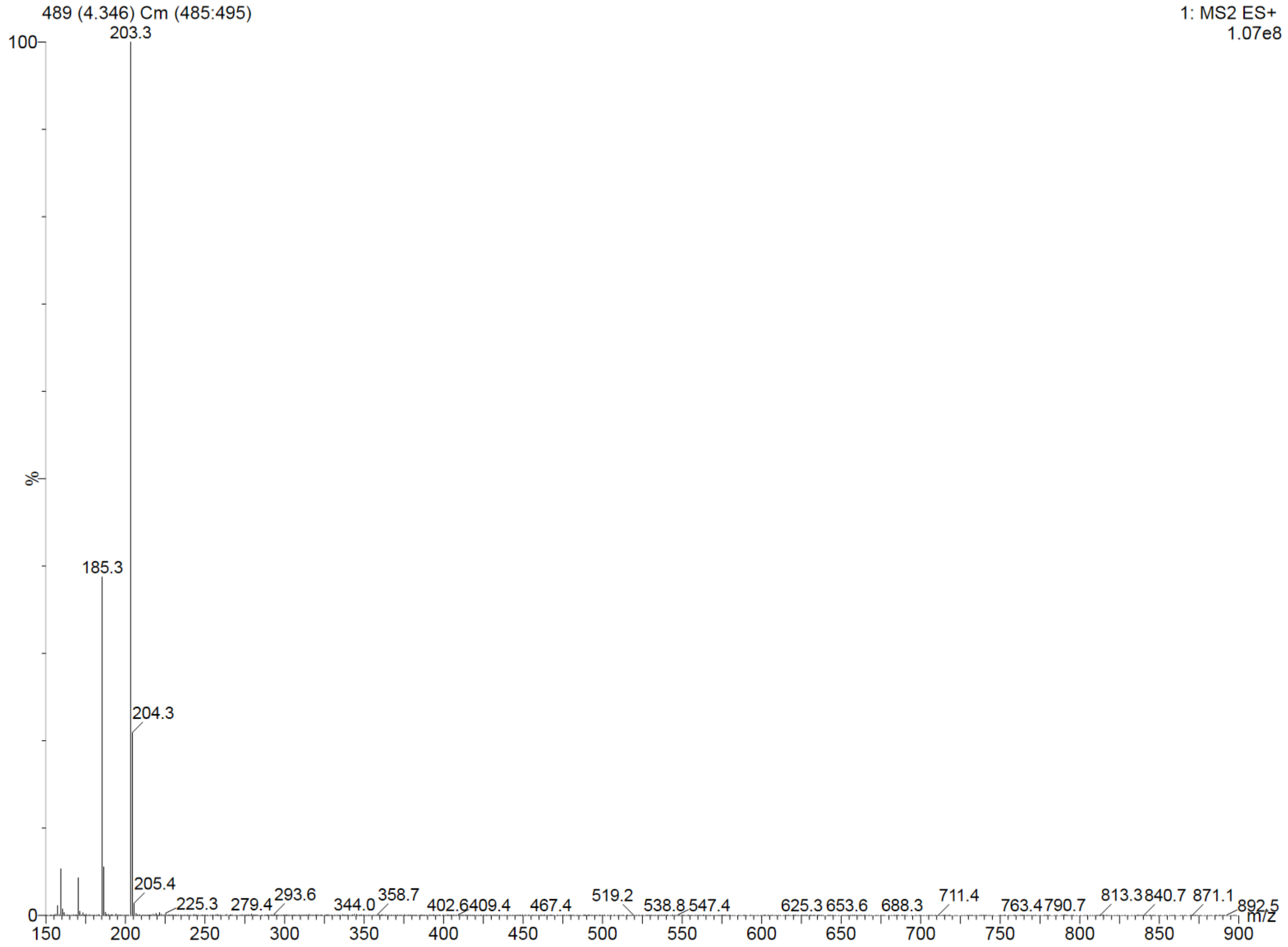
489 (4.346) Cm (485:495)
1: MS2 ES+
1.07e8
100
%
0
203.3
185.3
204.3
205.4
225.3
279.4
293.6
344.0
358.7
402.6
409.4
467.4
519.2
538.8
547.4
625.3
653.6
688.3
711.4
763.4
790.7
813.3
840.7
871.1
892.5
m/z
150
200
250
300
350
400
450
500
550
600
650
700
750
800
850
900

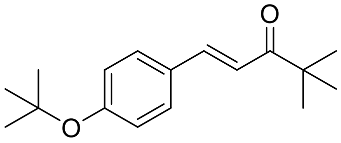

11

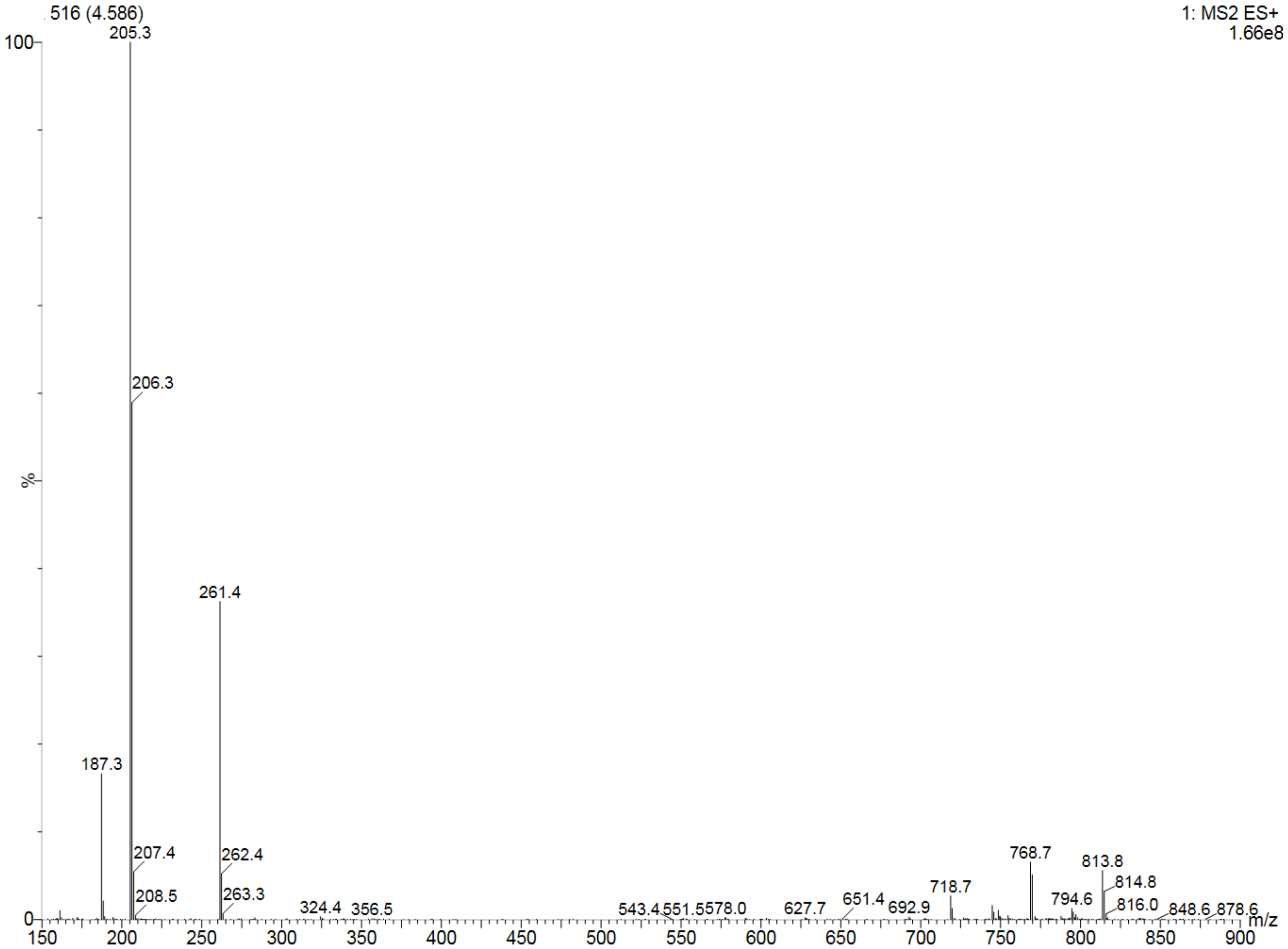
516 (4.586)
1: MS2 ES+
1.66e8
100
%
0
205.3
206.3
261.4
187.3
207.4
262.4
208.5
263.3
324.4
356.5
543.4
551.5
578.0
627.7
651.4
692.9
718.7
768.7
794.6
813.8
814.8
816.0
848.6
878.6
m/z
150
200
250
300
350
400
450
500
550
600
650
700
750
800
850
900

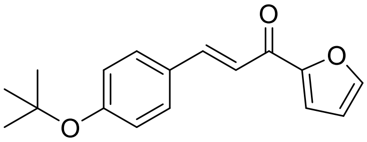

12

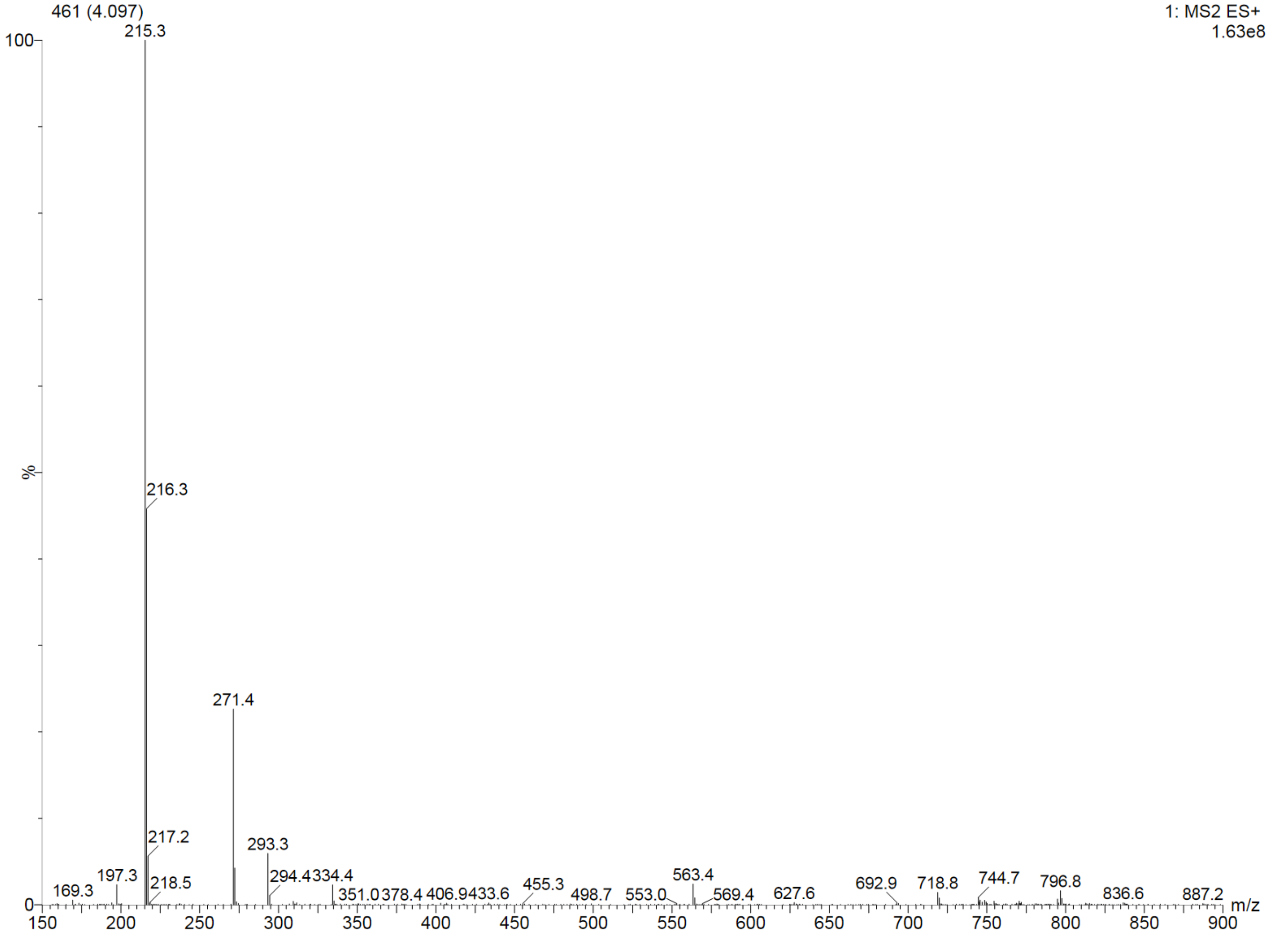
461 (4.097)
1: MS2 ES+
1.63e8
100
%
0
215.3
216.3
271.4
217.2
293.3
197.3
169.3
218.5
294.4
334.4
351.0
378.4
406.9
433.6
455.3
498.7
553.0
563.4
569.4
627.6
692.9
718.8
744.7
796.8
836.6
887.2
m/z
150
200
250
300
350
400
450
500
550
600
650
700
750
800
850
900

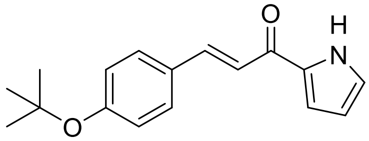
O
H
N
O

13

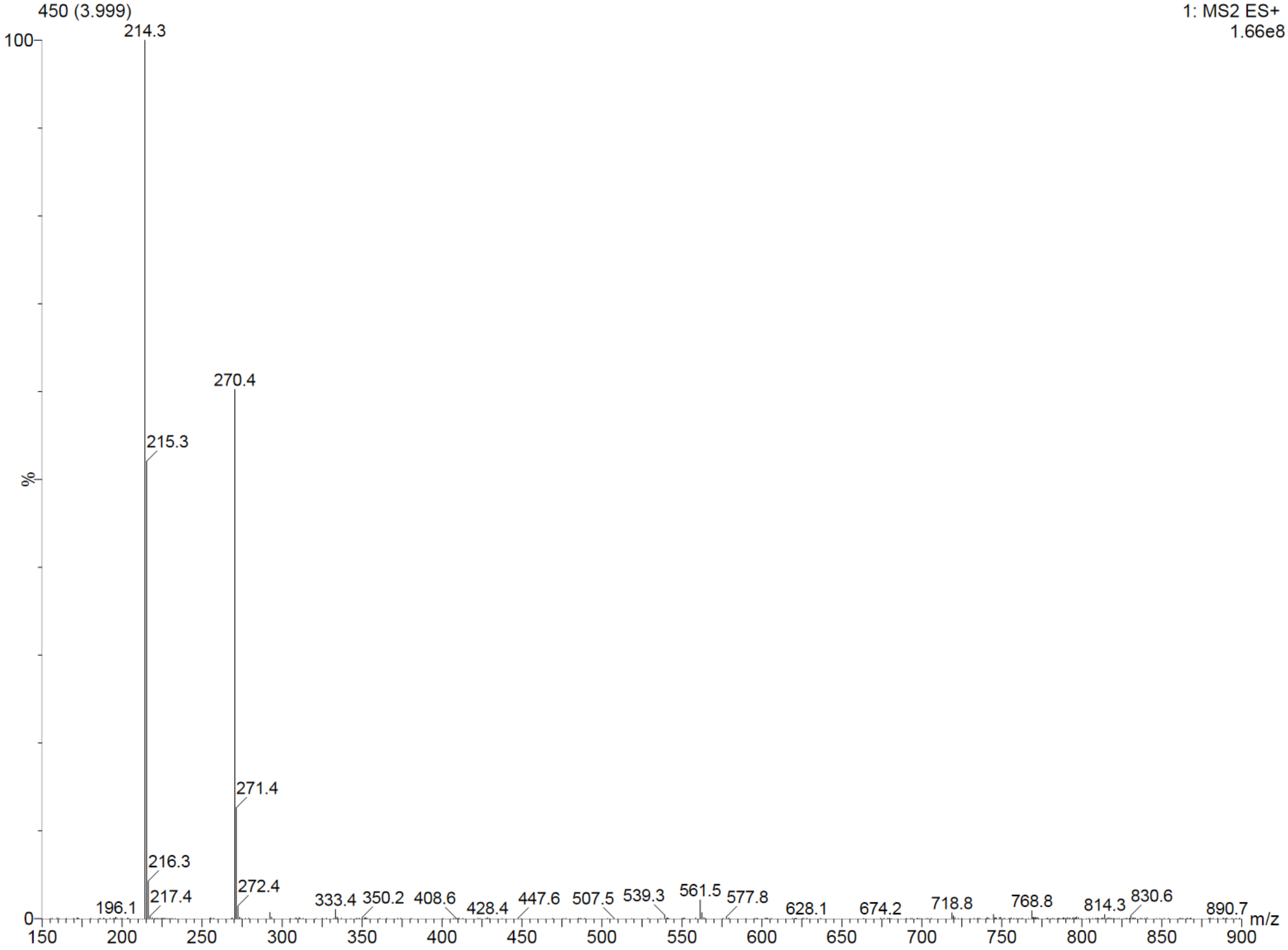
450 (3.999)
1: MS2 ES+
1.66e8
100
%
0
214.3
215.3
216.3
217.4
196.1
270.4
271.4
272.4
333.4
350.2
408.6
428.4
447.6
507.5
539.3
561.5
577.8
628.1
674.2
718.8
768.8
814.3
830.6
890.7
m/z
150
200
250
300
350
400
450
500
550
600
650
700
750
800
850
900

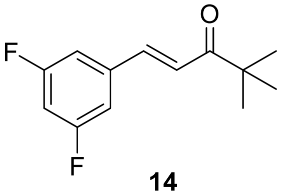

F
O
F

14

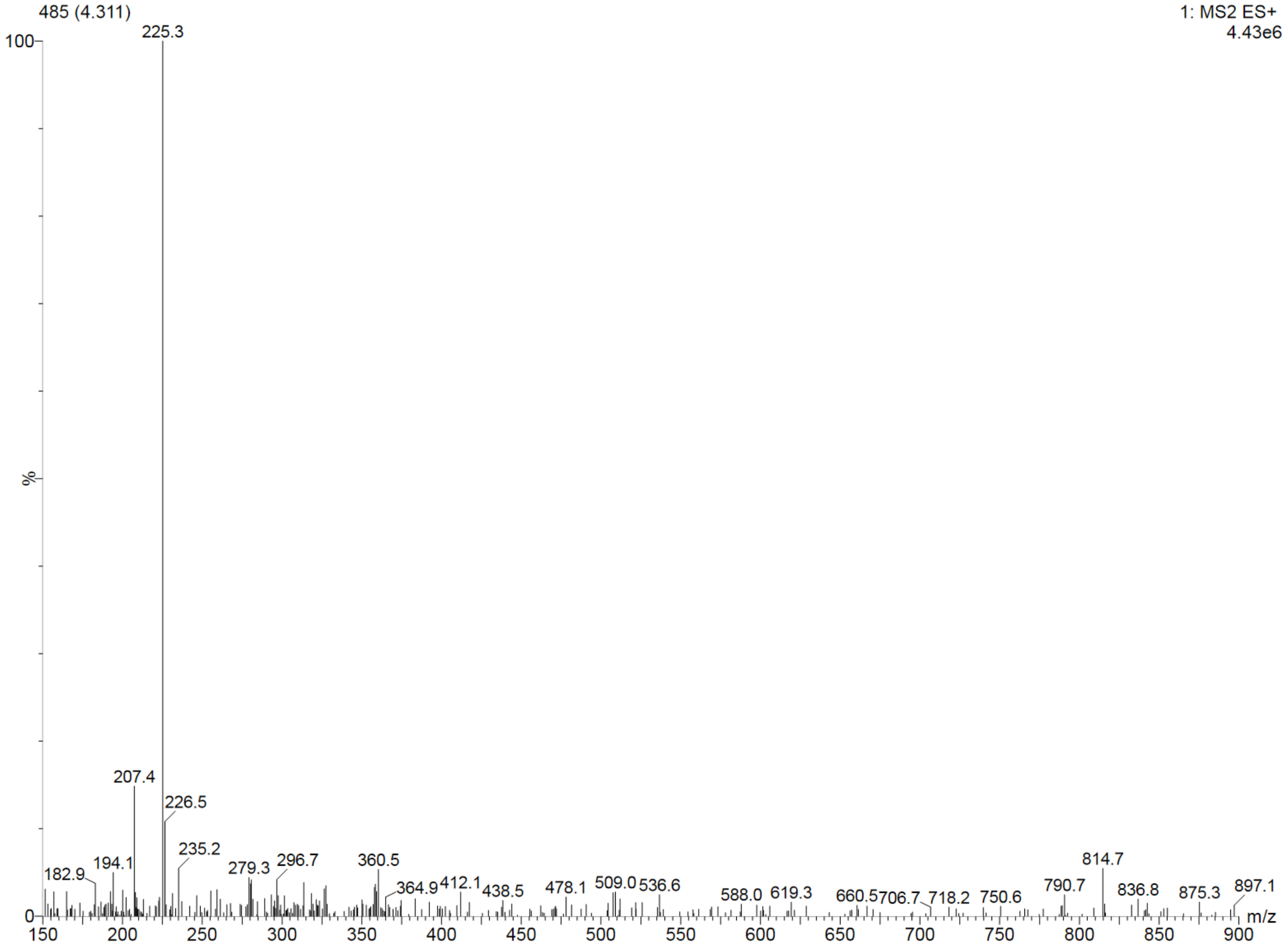

485 (4.311)
1: MS2 ES+
4.43e6
100
%
0
225.3
207.4
226.5
235.2
182.9
194.1
279.3
296.7
360.5
364.9
412.1
438.5
478.1
509.0
536.6
588.0
619.3
660.5
706.7
718.2
750.6
790.7
814.7
836.8
875.3
897.1
m/z
150
200
250
300
350
400
450
500
550
600
650
700
750
800
850
900

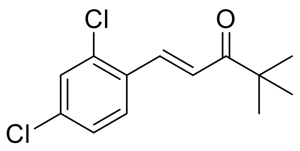
Cl
O
Cl

15

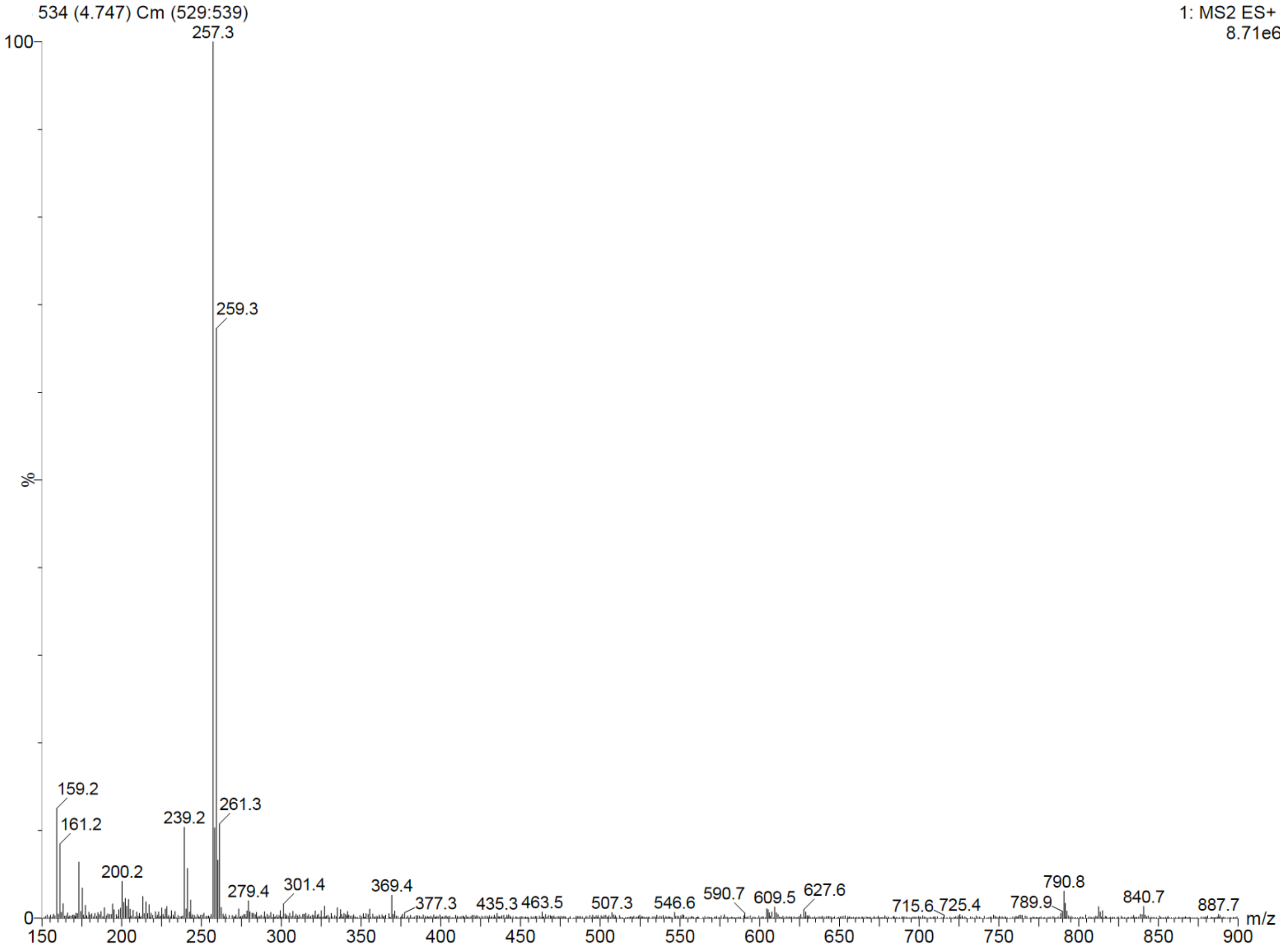
534 (4.747) Cm (529:539)
1: MS2 ES+
8.71e6
100
%
0
257.3
259.3
159.2
161.2
239.2
261.3
200.2
279.4
301.4
369.4
377.3
435.3
463.5
507.3
546.6
590.7
609.5
627.6
715.6
725.4
789.9
790.8
840.7
887.7
m/z
150
200
250
300
350
400
450
500
550
600
650
700
750
800
850
900

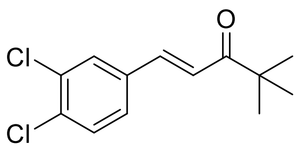
O
Cl
Cl

16

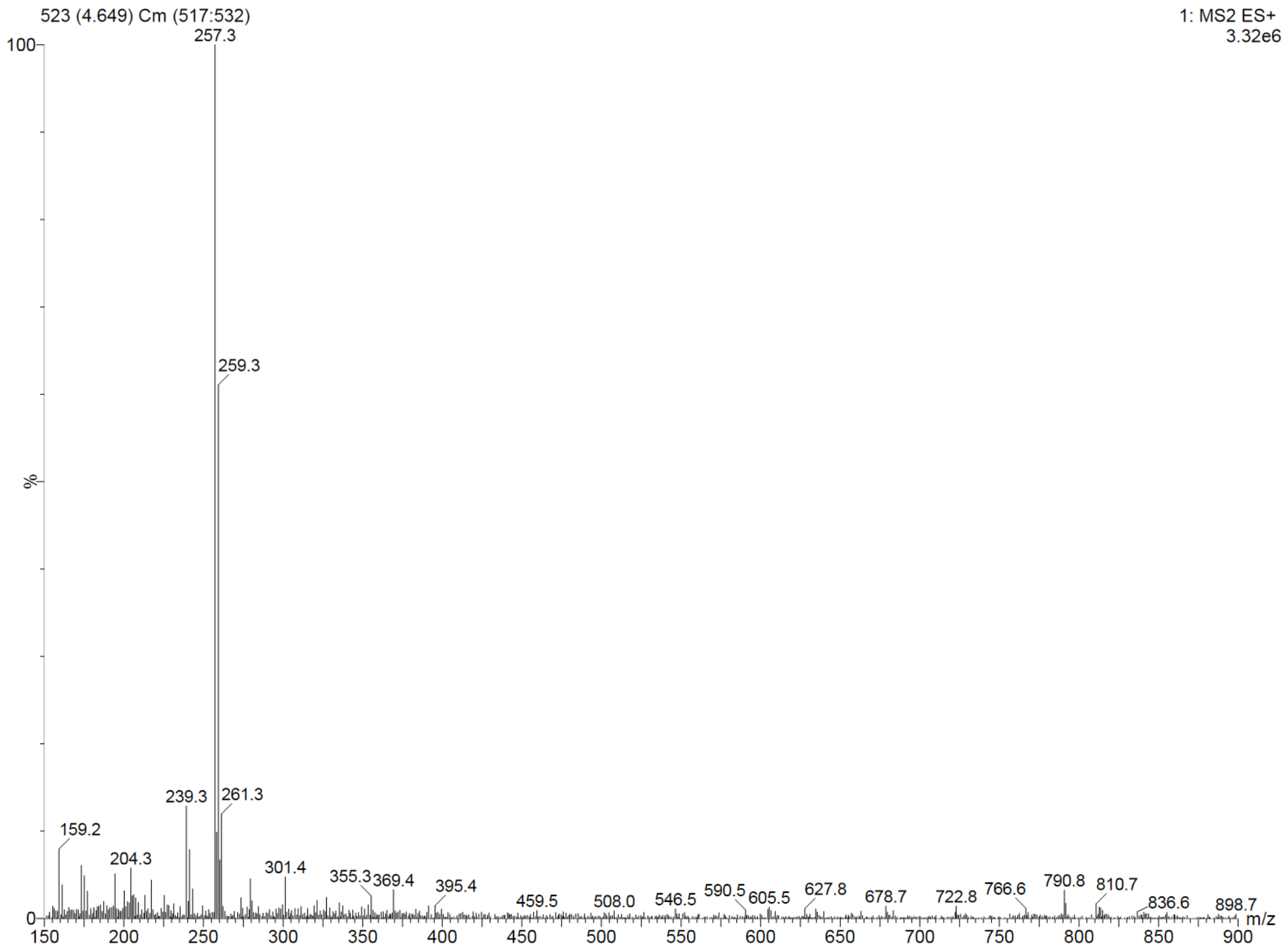
523 (4.649) Cm (517:532)
1: MS2 ES+
3.32e6
257.3
259.3
239.3
261.3
159.2
204.3
301.4
355.3
369.4
395.4
459.5
508.0
546.5
590.5
605.5
627.8
678.7
722.8
766.6
790.8
810.7
836.6
898.7
100
%
0
m/z
150
200
250
300
350
400
450
500
550
600
650
700
750
800
850
900

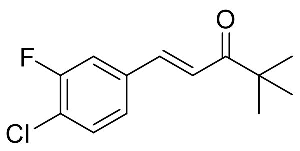
F
O
Cl

17

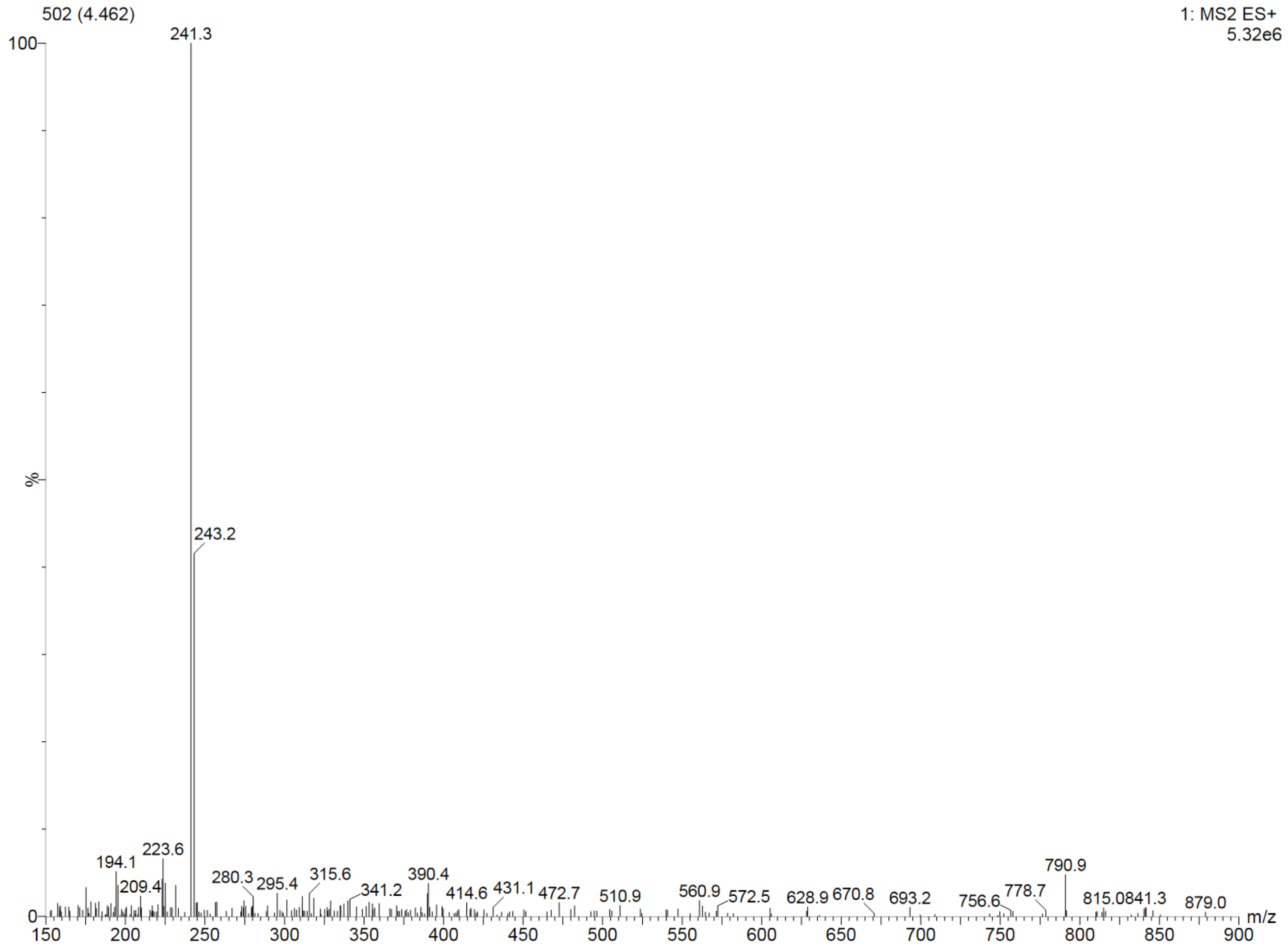
502 (4.462)
1: MS2 ES+
5.32e6
100
%
0
241.3
243.2
194.1
223.6
209.4
280.3
295.4
315.6
341.2
390.4
414.6
431.1
472.7
510.9
560.9
572.5
628.9
670.8
693.2
756.6
778.7
790.9
815.0841.3
879.0
150
200
250
300
350
400
450
500
550
600
650
700
750
800
850
900
m/z

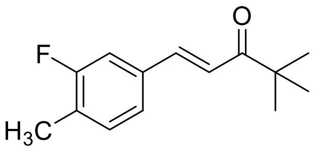
O
F
H_3C

18

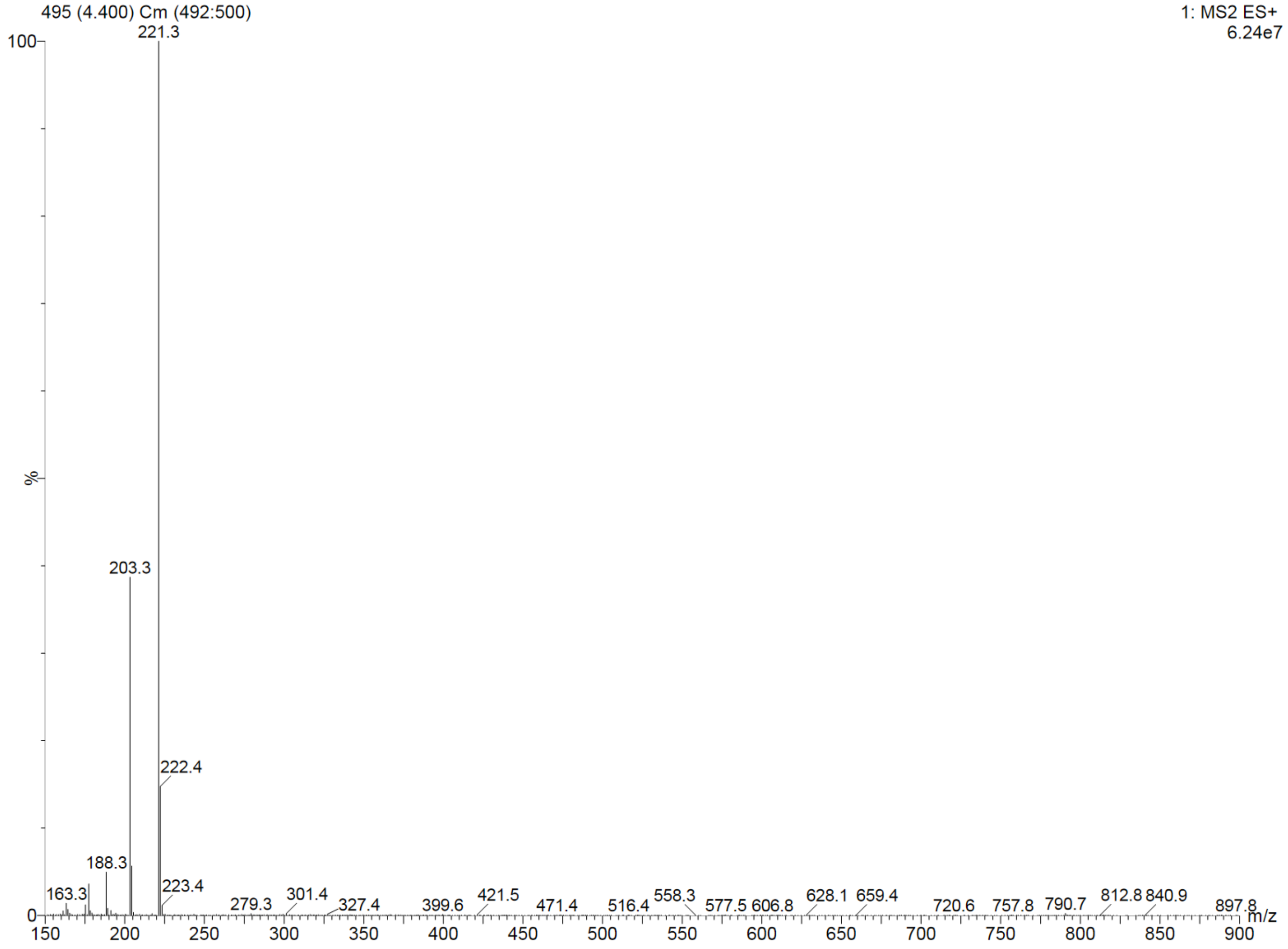
495 (4.400) Cm (492:500)
1: MS2 ES+
6.24e7
100
%
0
221.3
203.3
222.4
188.3
163.3
223.4
279.3
301.4
327.4
399.6
421.5
471.4
516.4
558.3
577.5
606.8
628.1
659.4
720.6
757.8
790.7
812.8
840.9
897.8
m/z
150
200
250
300
350
400
450
500
550
600
650
700
750
800
850
900

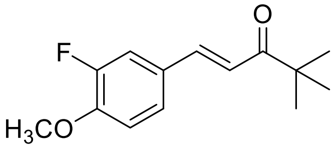
F
O
H3CO

19

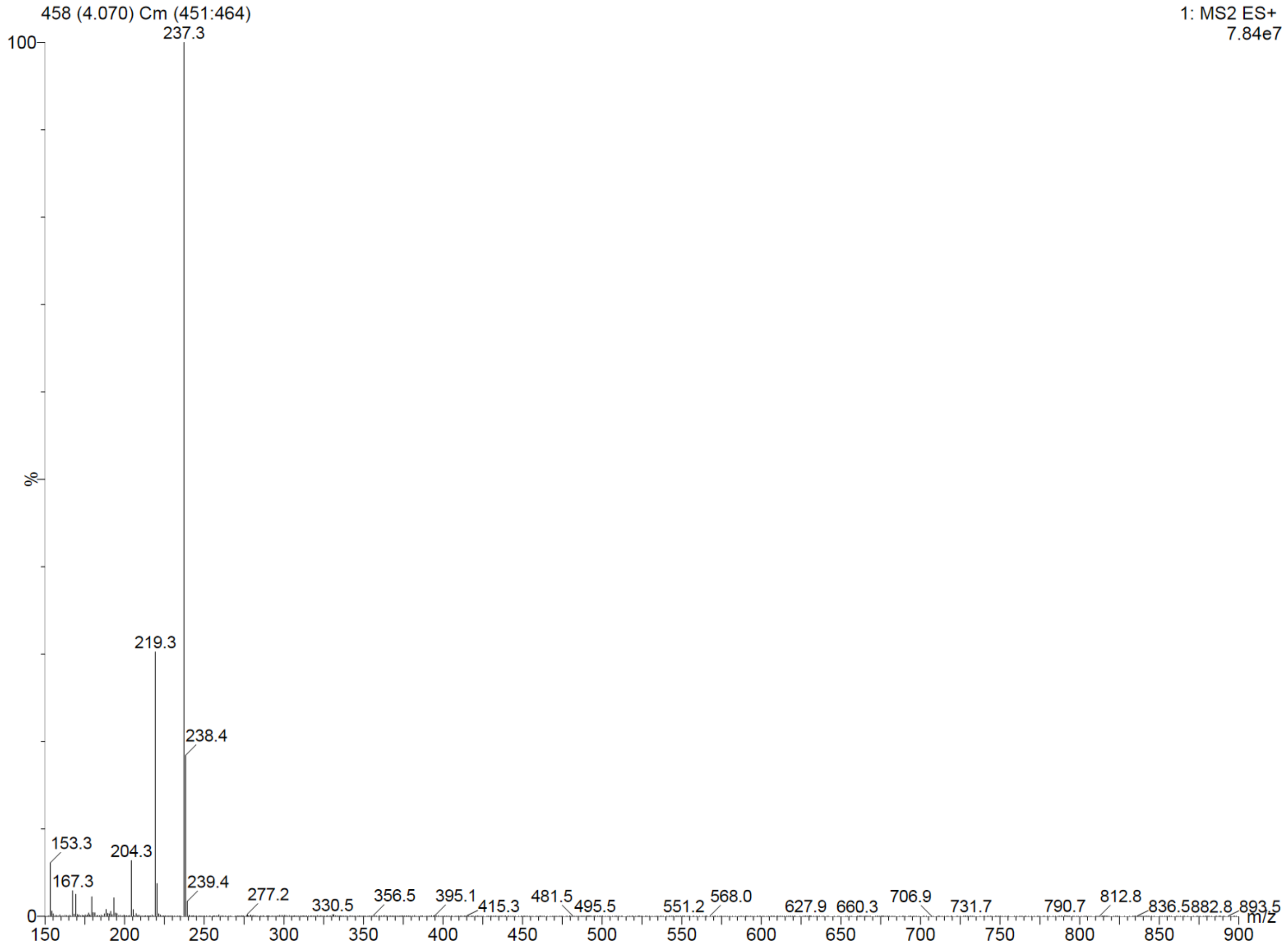
458 (4.070) Cm (451:464)
1: MS2 ES+
7.84e7
237.3
219.3
238.4
153.3
204.3
167.3
239.4
277.2
330.5
356.5
395.1
415.3
481.5
495.5
551.2
568.0
627.9
660.3
706.9
731.7
790.7
812.8
836.5
882.8
893.5
%
m/z
100
0
150
200
250
300
350
400
450
500
550
600
650
700
750
800
850
900

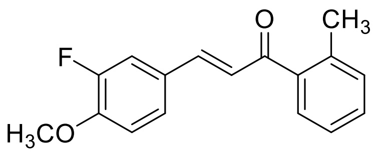
F
H3CO
O
CH3

20

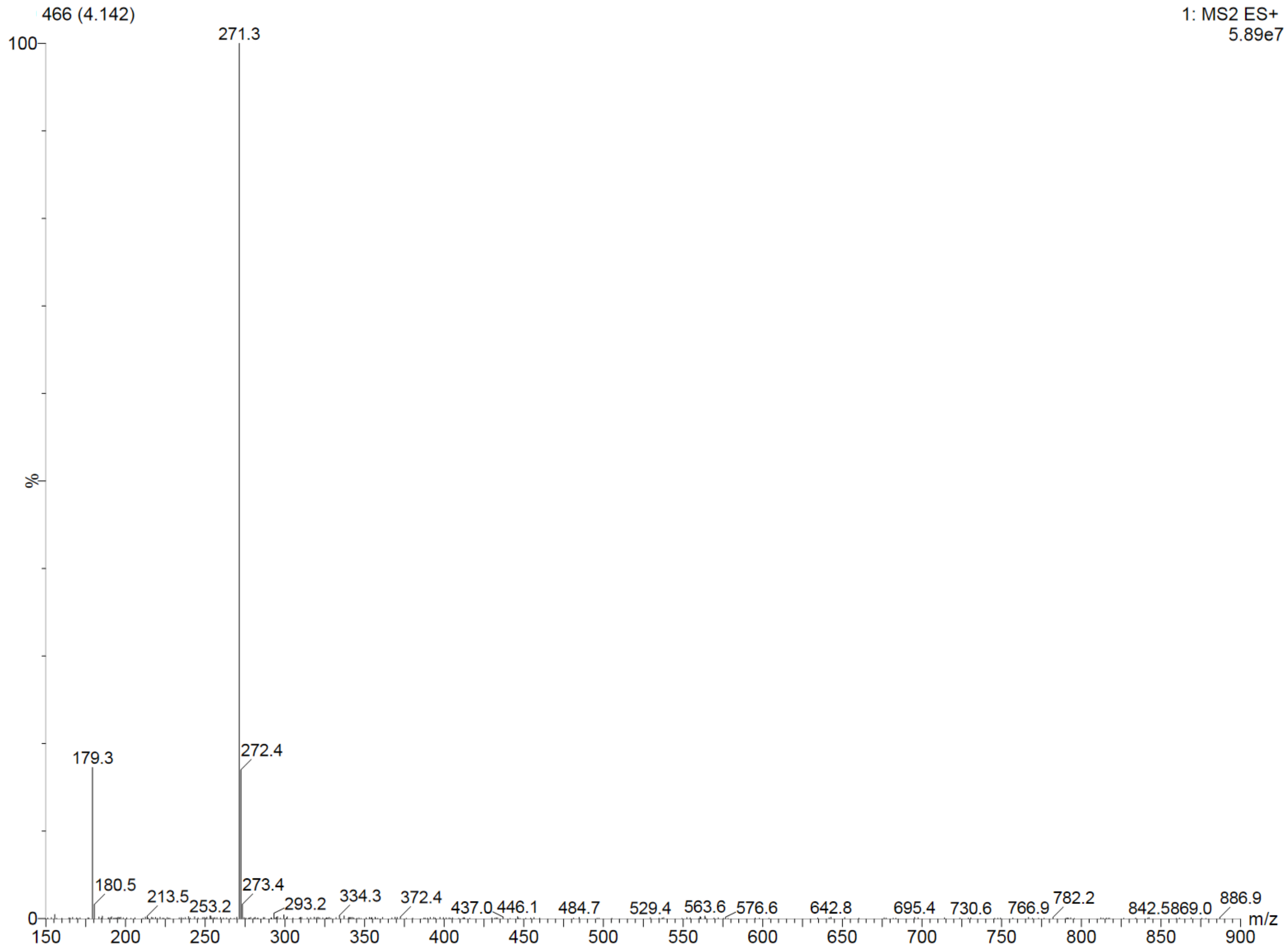
466 (4.142)
1: MS2 ES+
5.89e7
271.3
179.3
272.4
180.5
213.5
253.2
273.4
293.2
334.3
372.4
437.0
446.1
484.7
529.4
563.6
576.6
642.8
695.4
730.6
766.9
782.2
842.5
869.0
886.9
%
m/z
0
100
150
200
250
300
350
400
450
500
550
600
650
700
750
800
850
900

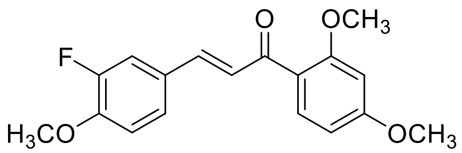

O
OCH3
F
H3CO
OCH3

21

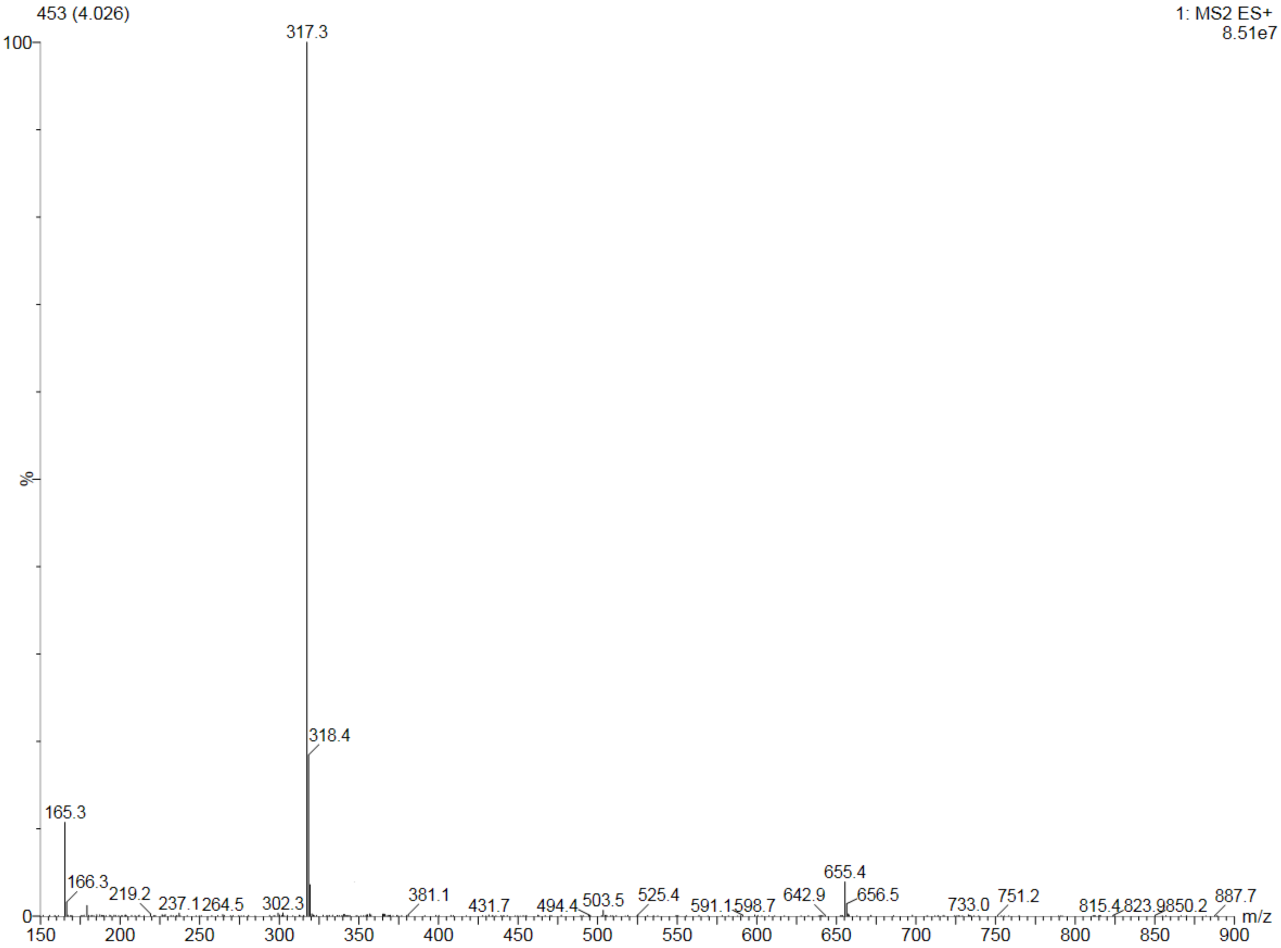

453 (4.026)
1: MS2 ES+
8.51e7
100
%
0
317.3
318.4
165.3
166.3
219.2
237.1
264.5
302.3
381.1
431.7
494.4
503.5
525.4
591.1
598.7
642.9
655.4
656.5
733.0
751.2
815.4
823.9
850.2
887.7
m/z
150
200
250
300
350
400
450
500
550
600
650
700
750
800
850
900

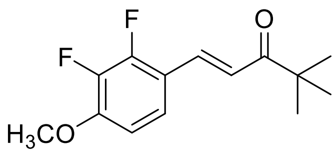
F
F
O
H3CO

22

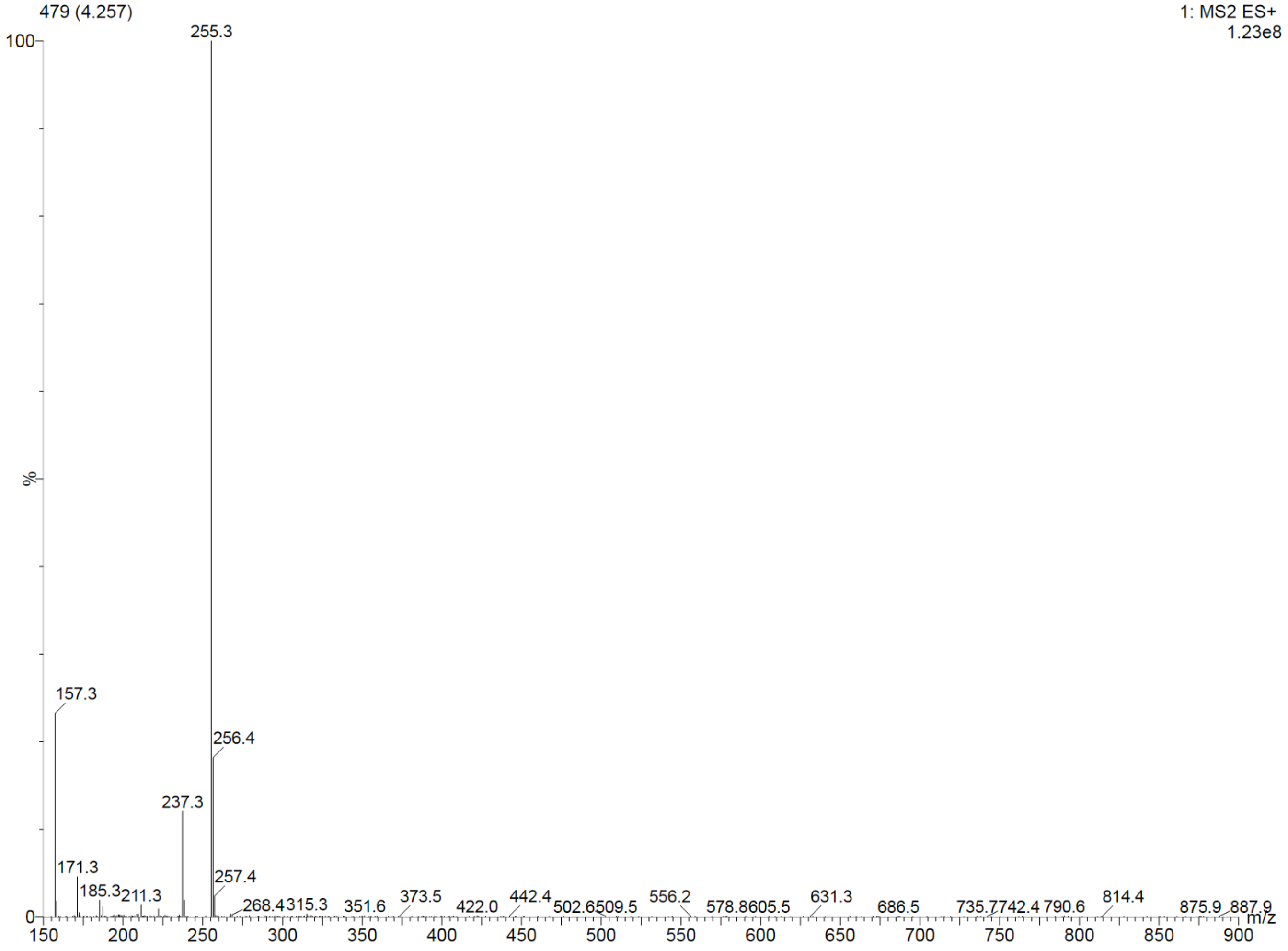
479 (4.257)
1: MS2 ES+
1.23e8
100
%
0
255.3
157.3
256.4
237.3
171.3
257.4
185.3
211.3
268.4
315.3
351.6
373.5
422.0
442.4
502.6
509.5
556.2
578.8
605.5
631.3
686.5
735.7
742.4
790.6
814.4
875.9
887.9
m/z
150
200
250
300
350
400
450
500
550
600
650
700
750
800
850
900

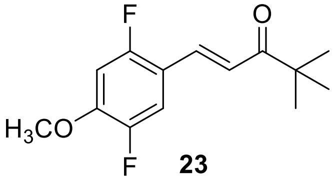
F
O
H_3CO
F

23

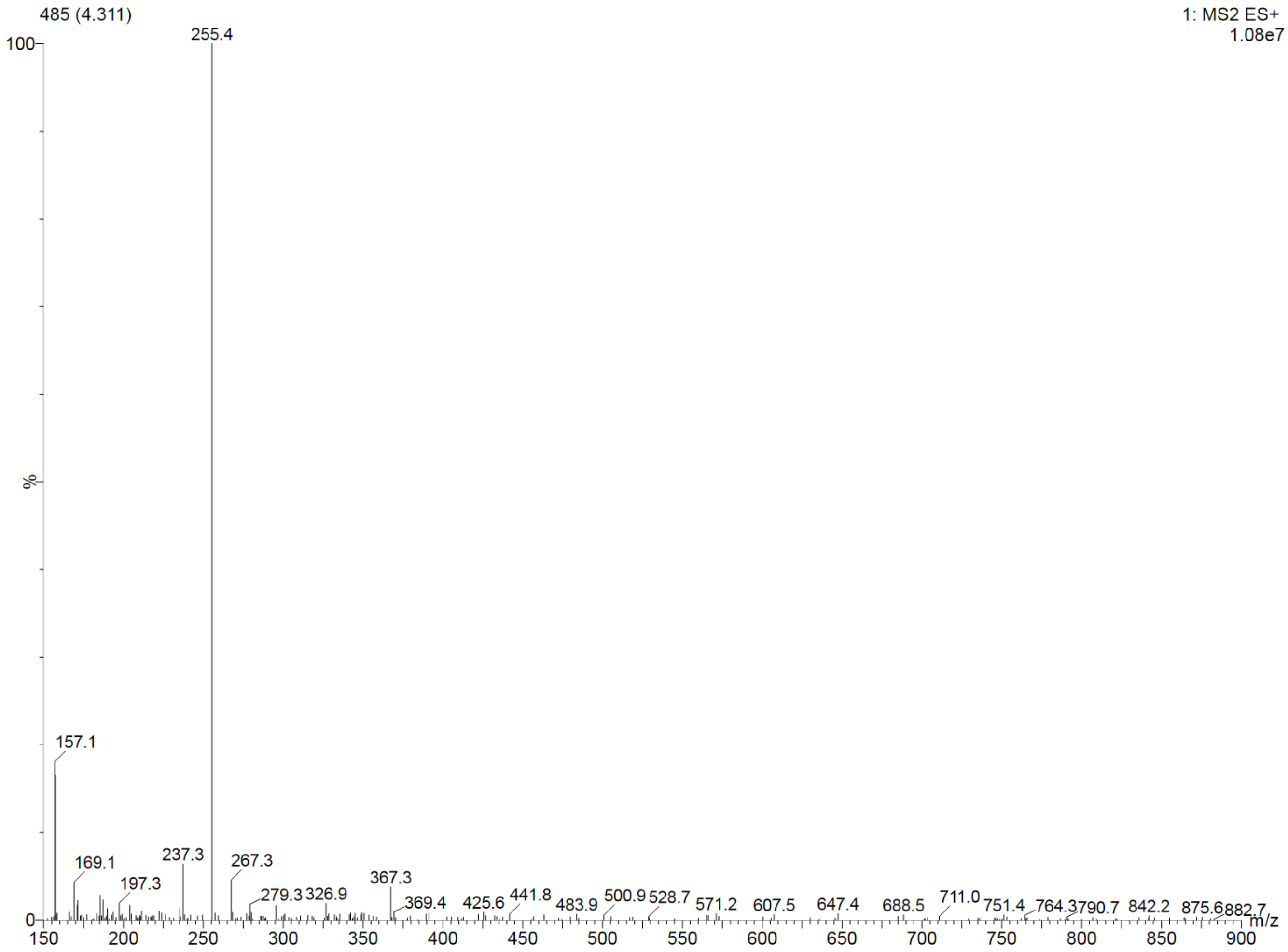
485 (4.311)
1: MS2 ES+
1.08e7
255.4
100
%
0
157.1
169.1
197.3
237.3
267.3
279.3
326.9
367.3
369.4
425.6
441.8
483.9
500.9
528.7
571.2
607.5
647.4
688.5
711.0
751.4
764.3
790.7
842.2
875.6
882.7
m/z
150
200
250
300
350
400
450
500
550
600
650
700
750
800
850
900

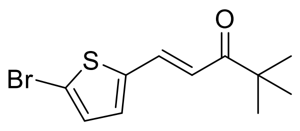

Br
S
O

24

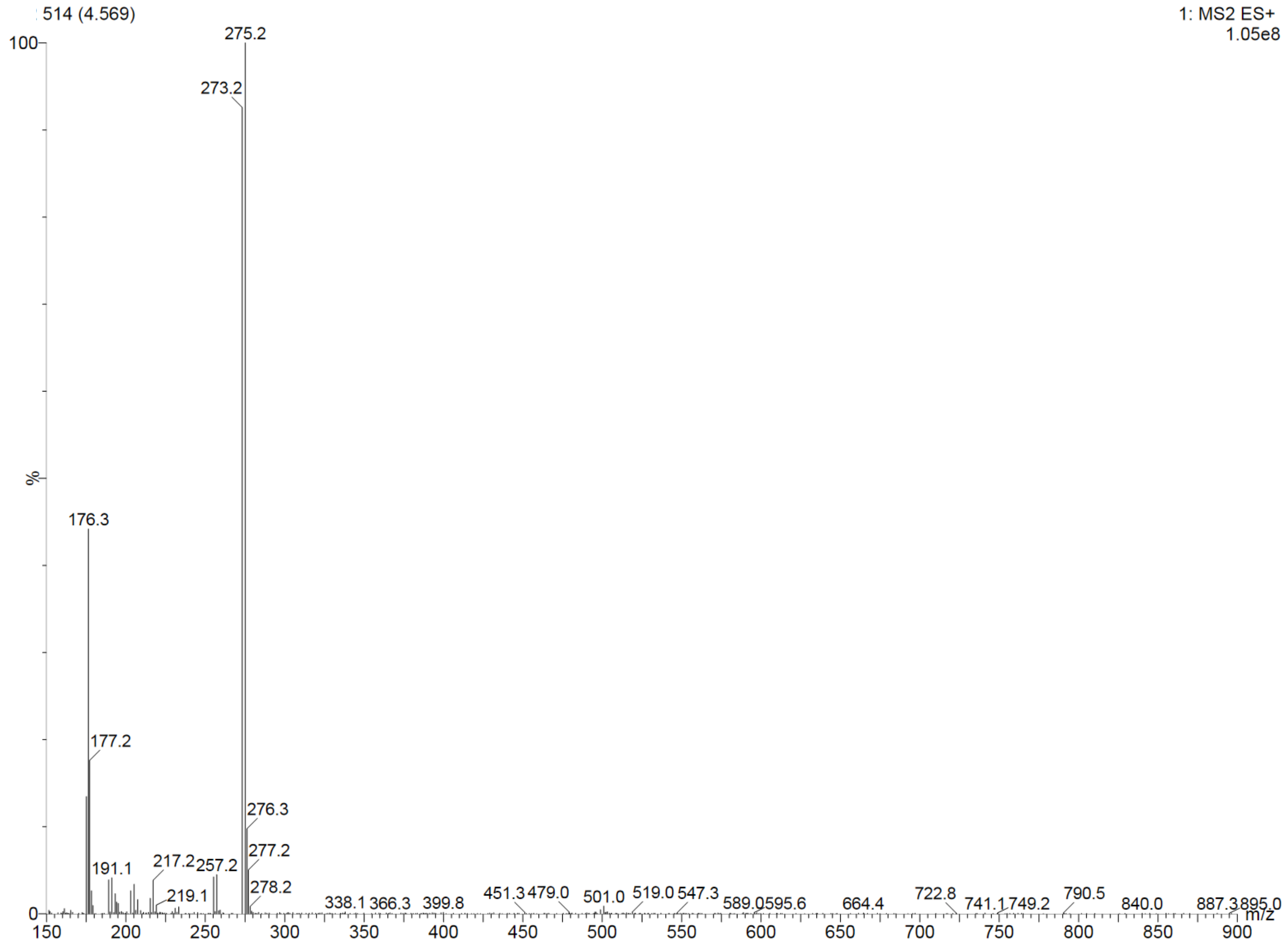

514 (4.569)
1: MS2 ES+
1.05e8
100
%
0
275.2
273.2
176.3
177.2
276.3
277.2
191.1
217.2
257.2
219.1
278.2
338.1
366.3
399.8
451.3
479.0
501.0
519.0
547.3
589.0
595.6
664.4
722.8
741.1
749.2
790.5
840.0
887.3
895.0
m/z
150
200
250
300
350
400
450
500
550
600
650
700
750
800
850
900

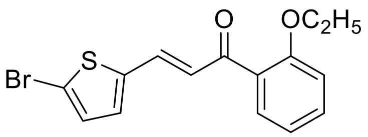

Br
S
O
OC_2H_5

25

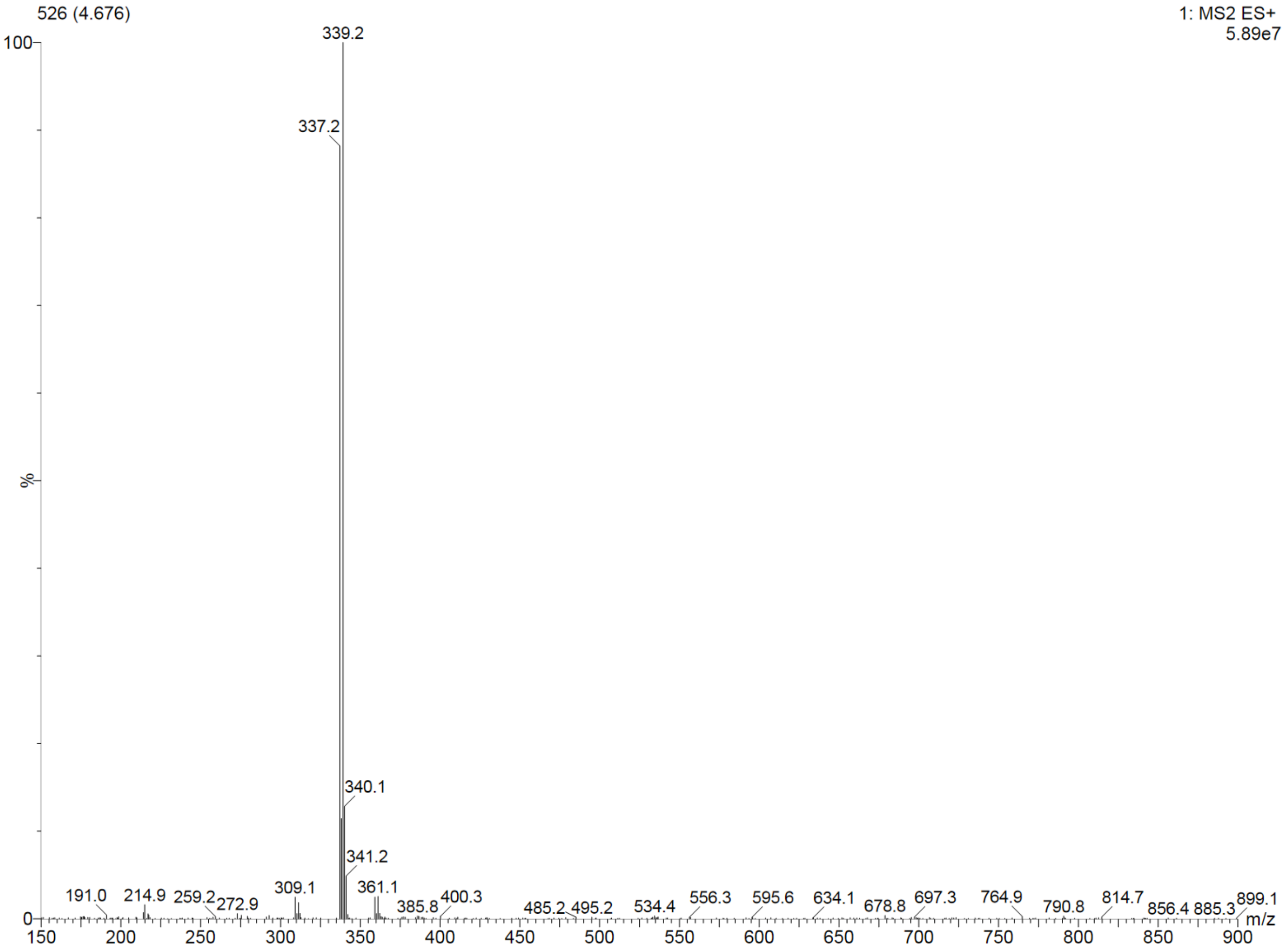

526 (4.676)
1: MS2 ES+
5.89e7
100
%
0
339.2
337.2
340.1
341.2
361.1
191.0
214.9
259.2
272.9
309.1
385.8
400.3
485.2
495.2
534.4
556.3
595.6
634.1
678.8
697.3
764.9
790.8
814.7
856.4
885.3
899.1
m/z
150
200
250
300
350
400
450
500
550
600
650
700
750
800
850
900

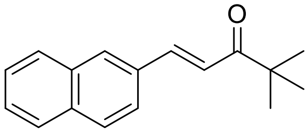
O

26

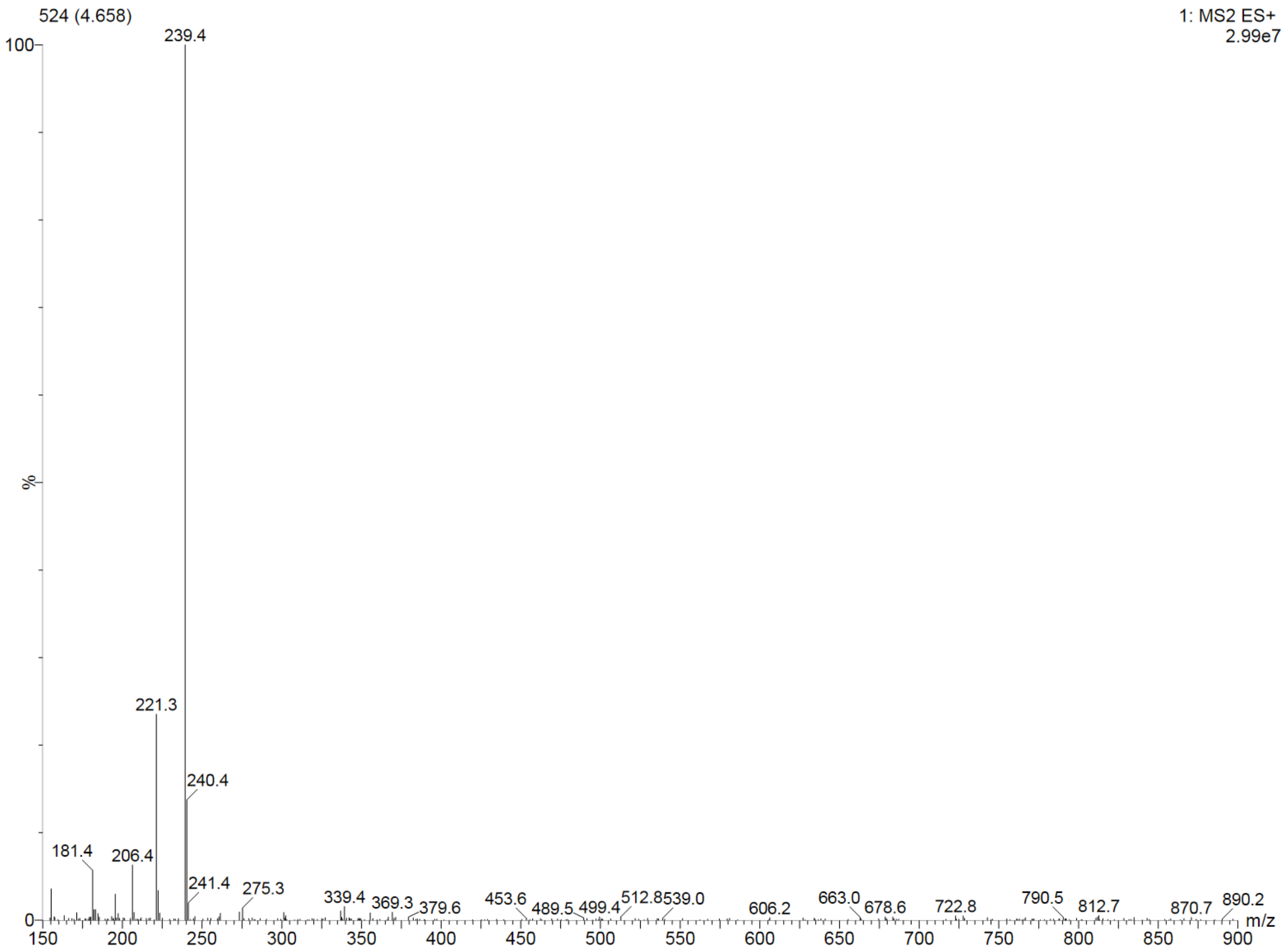
524 (4.658)
1: MS2 ES+
2.99e7
100
%
0
239.4
221.3
240.4
181.4
206.4
241.4
275.3
339.4
369.3
379.6
453.6
489.5
499.4
512.8
539.0
606.2
663.0
678.6
722.8
790.5
812.7
870.7
890.2
m/z
150
200
250
300
350
400
450
500
550
600
650
700
750
800
850
900

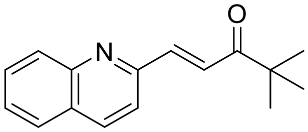

27

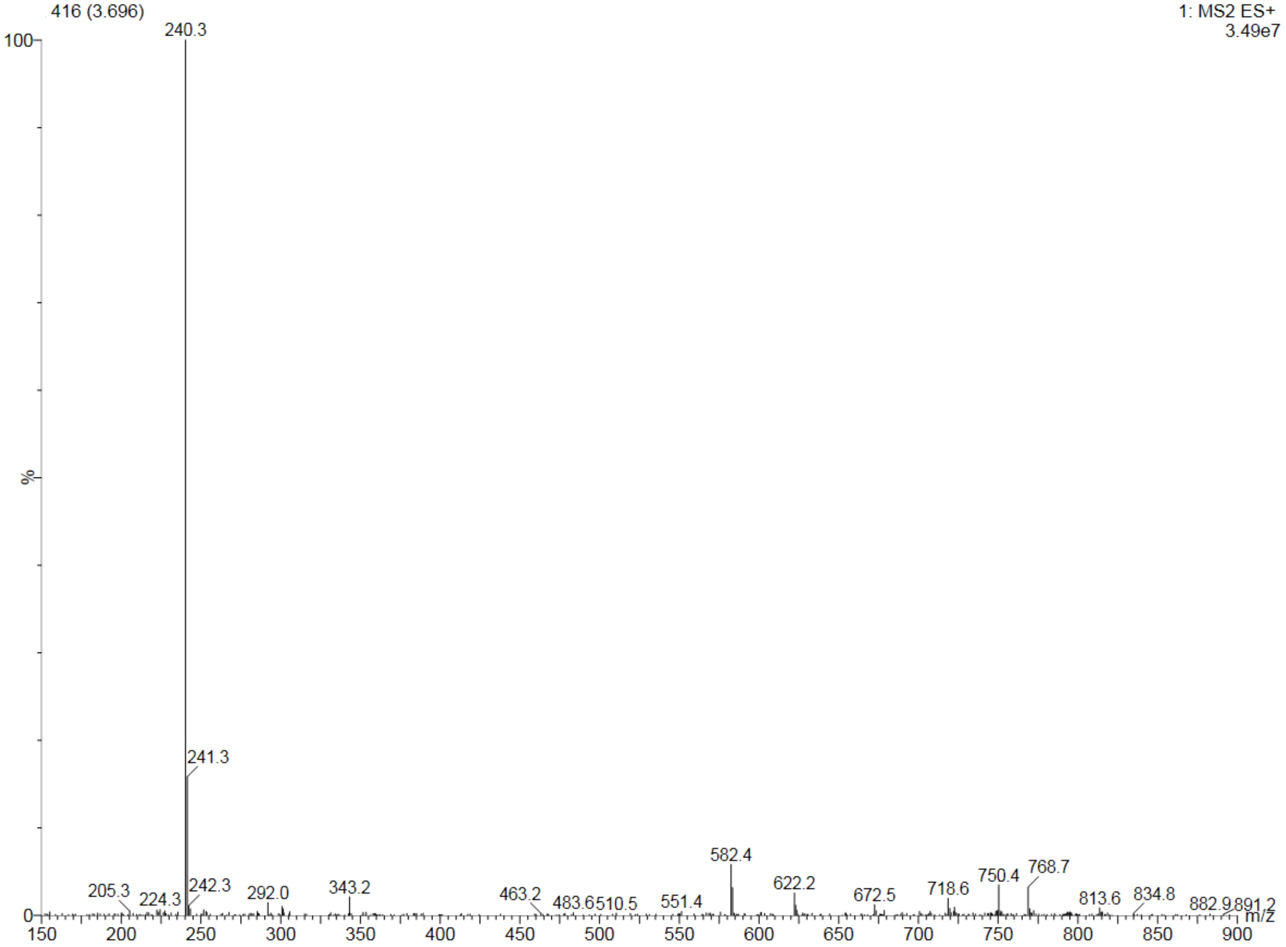
416 (3.696)
1: MS2 ES+
3.49e7
240.3
241.3
205.3
224.3
242.3
292.0
343.2
463.2
483.6
510.5
551.4
582.4
622.2
672.5
718.6
750.4
768.7
813.6
834.8
882.9
891.2
m/z
%

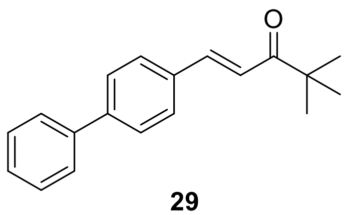
O

29

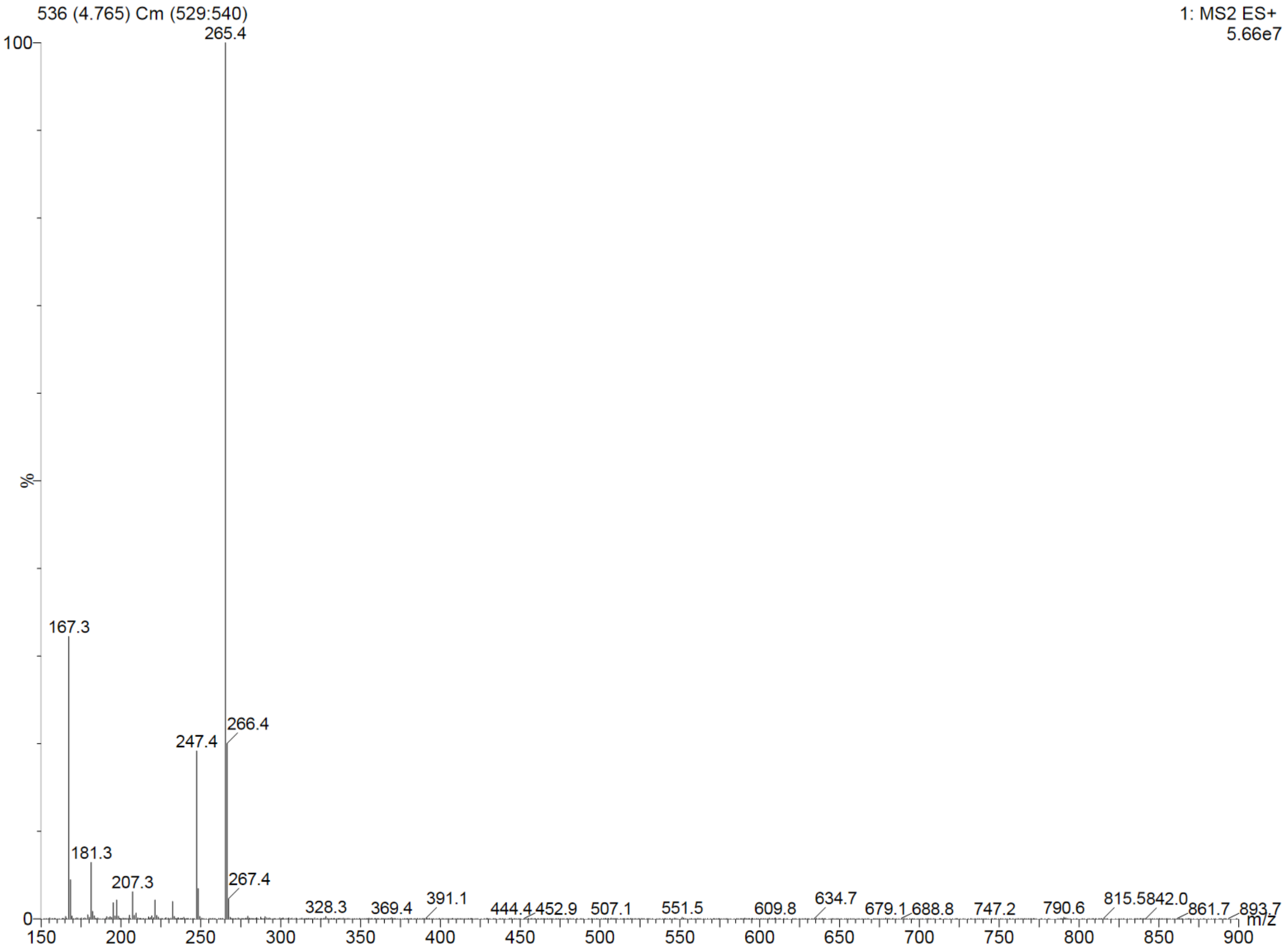
536 (4.765) Cm (529:540)
1: MS2 ES+
5.66e7
100
%
0
265.4
167.3
266.4
247.4
181.3
207.3
267.4
328.3
369.4
391.1
444.4
452.9
507.1
551.5
609.8
634.7
679.1
688.8
747.2
790.6
815.5
842.0
861.7
893.7
m/z
150
200
250
300
350
400
450
500
550
600
650
700
750
800
850
900

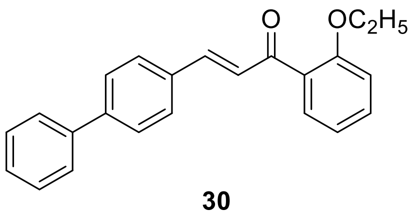
O
OC2H5

30

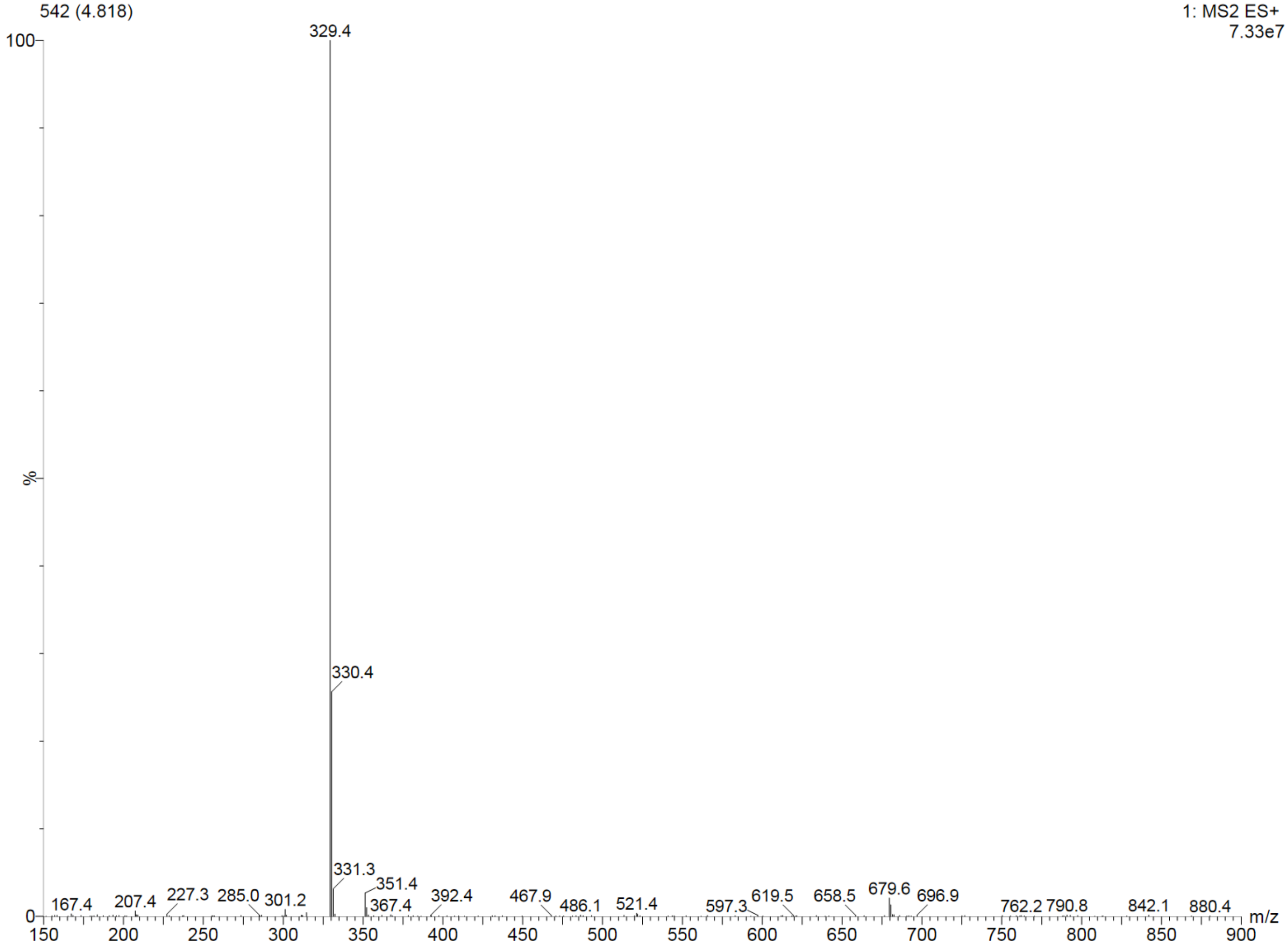
542 (4.818)
1: MS2 ES+
7.33e7
100
%
0
329.4
330.4
331.3
351.4
367.4
167.4
207.4
227.3
285.0
301.2
392.4
467.9
486.1
521.4
597.3
619.5
658.5
679.6
696.9
762.2
790.8
842.1
880.4
m/z
150
200
250
300
350
400
450
500
550
600
650
700
750
800
850
900

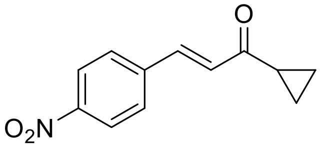
O
O_2N

1a

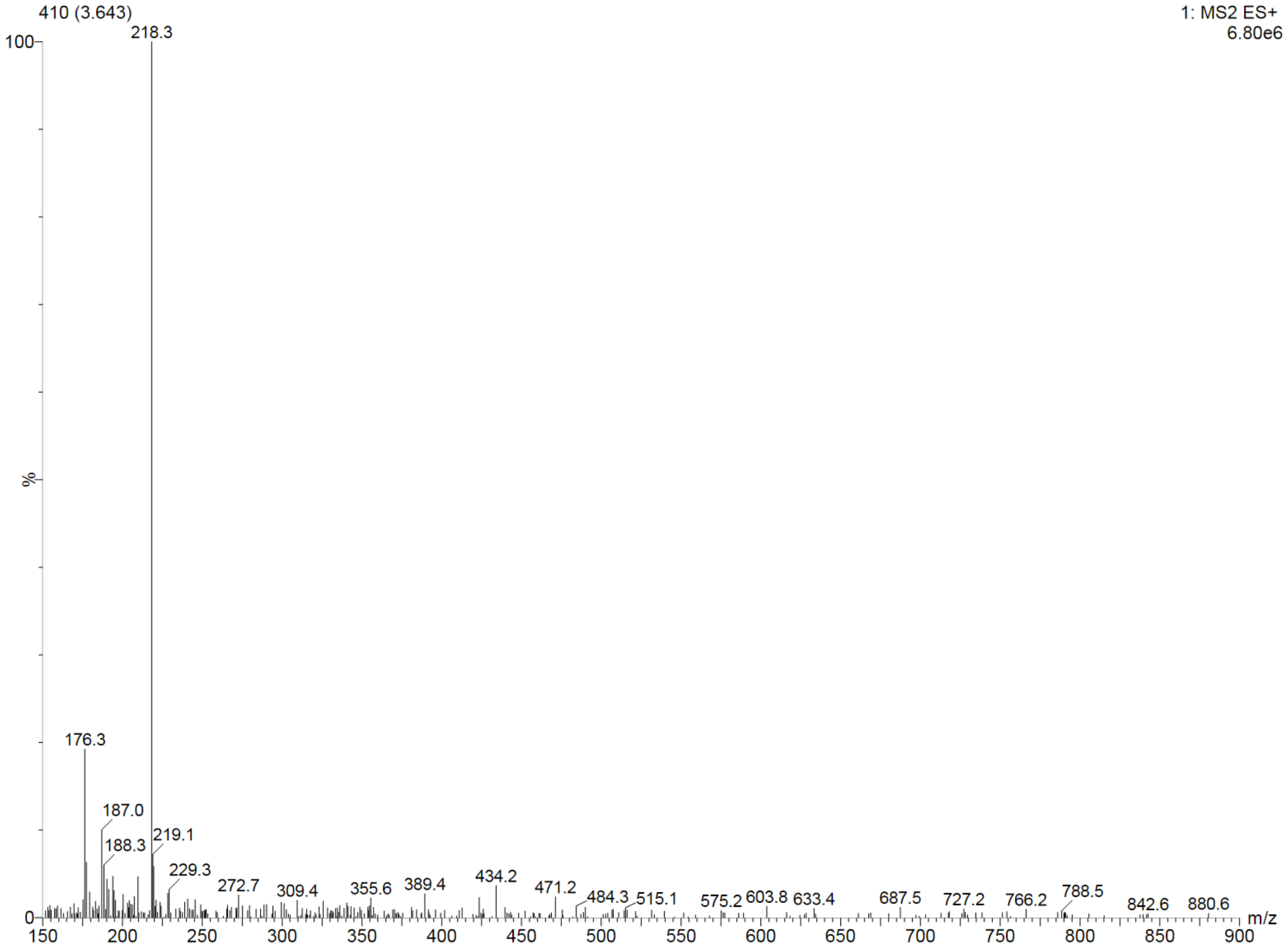
410 (3.643)
1: MS2 ES+
6.80e6
218.3
176.3
187.0
188.3
219.1
229.3
272.7
309.4
355.6
389.4
434.2
471.2
484.3
515.1
575.2
603.8
633.4
687.5
727.2
766.2
788.5
842.6
880.6
100
%
0
150
200
250
300
350
400
450
500
550
600
650
700
750
800
850
900
m/z

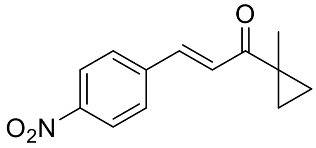
O
O_2N

2a

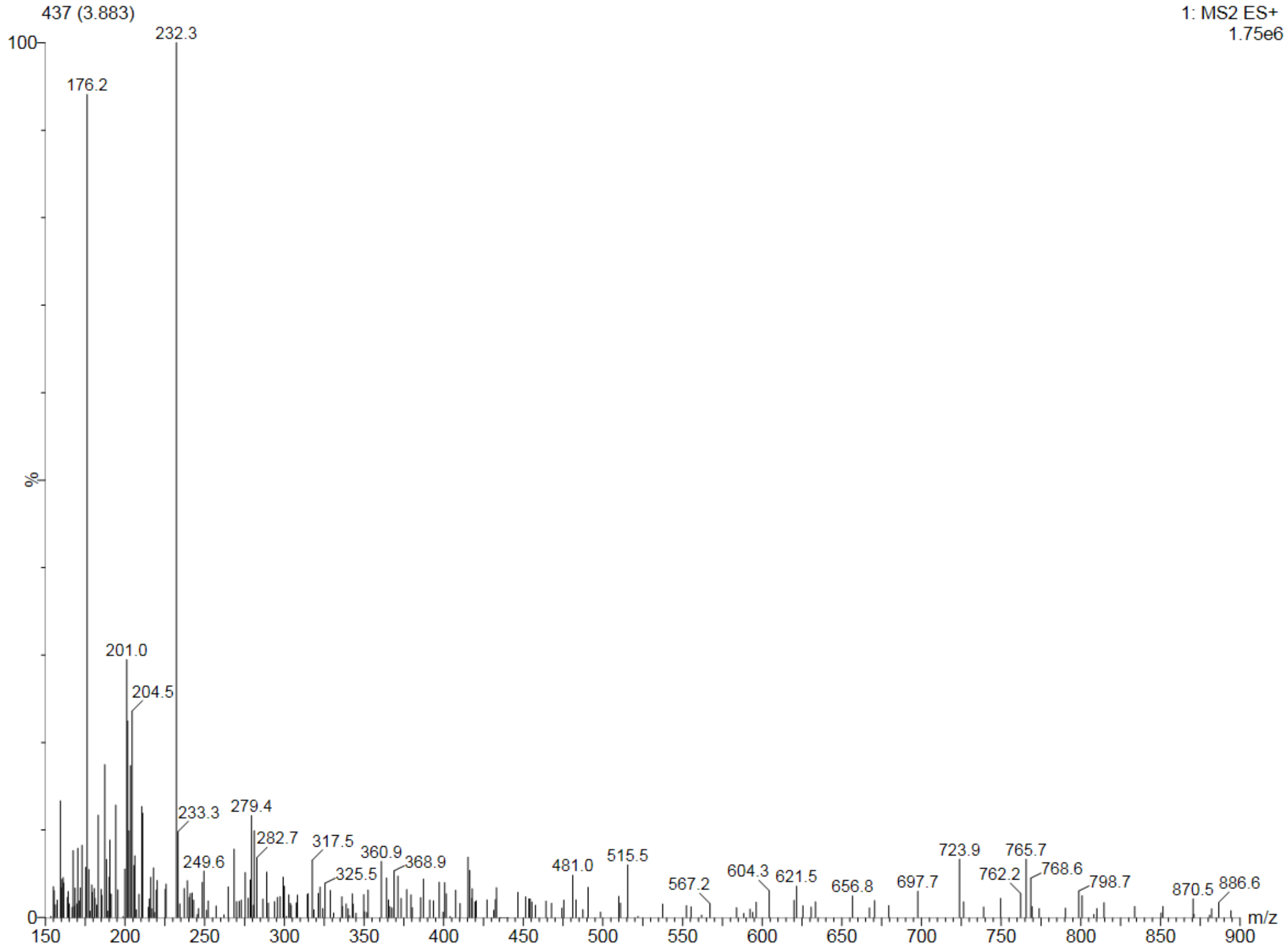
437 (3.883)
1: MS2 ES+
1.75e6
100
%
0
232.3
176.2
201.0
204.5
233.3
279.4
282.7
249.6
317.5
325.5
360.9
368.9
481.0
515.5
567.2
604.3
621.5
656.8
697.7
723.9
762.2
765.7
768.6
798.7
870.5
886.6
m/z
150
200
250
300
350
400
450
500
550
600
650
700
750
800
850
900

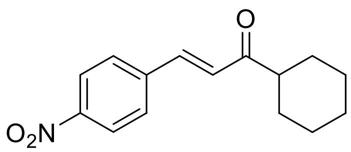
O
O_2N

3a

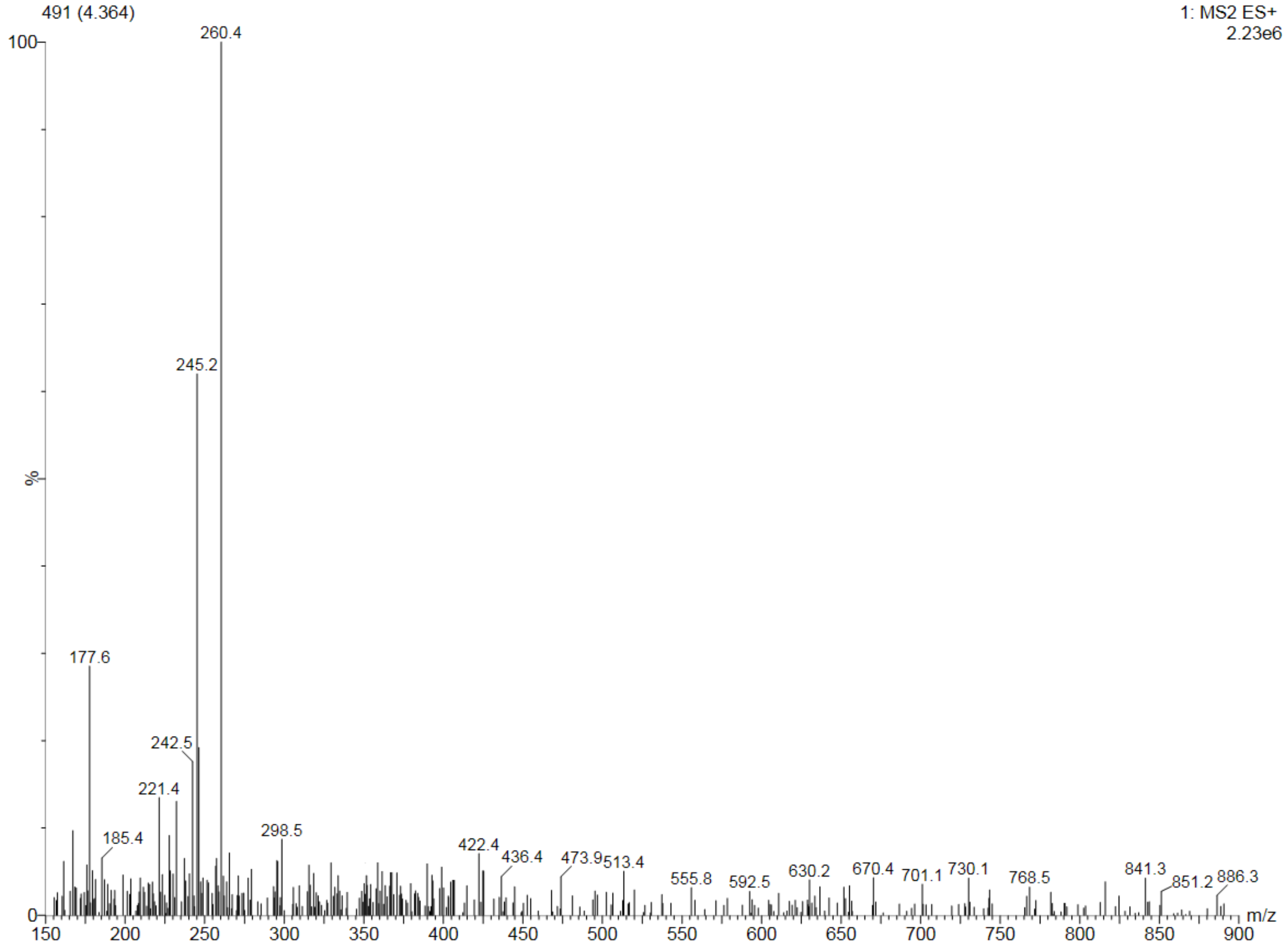
491 (4.364)
1: MS2 ES+
2.23e6
100
%
0
260.4
245.2
177.6
242.5
221.4
185.4
298.5
422.4
436.4
473.9
513.4
555.8
592.5
630.2
670.4
701.1
730.1
768.5
841.3
851.2
886.3
m/z
150
200
250
300
350
400
450
500
550
600
650
700
750
800
850
900

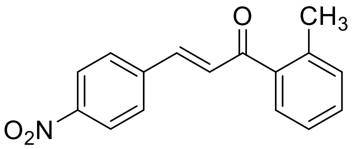
O
CH3
O2N

4a

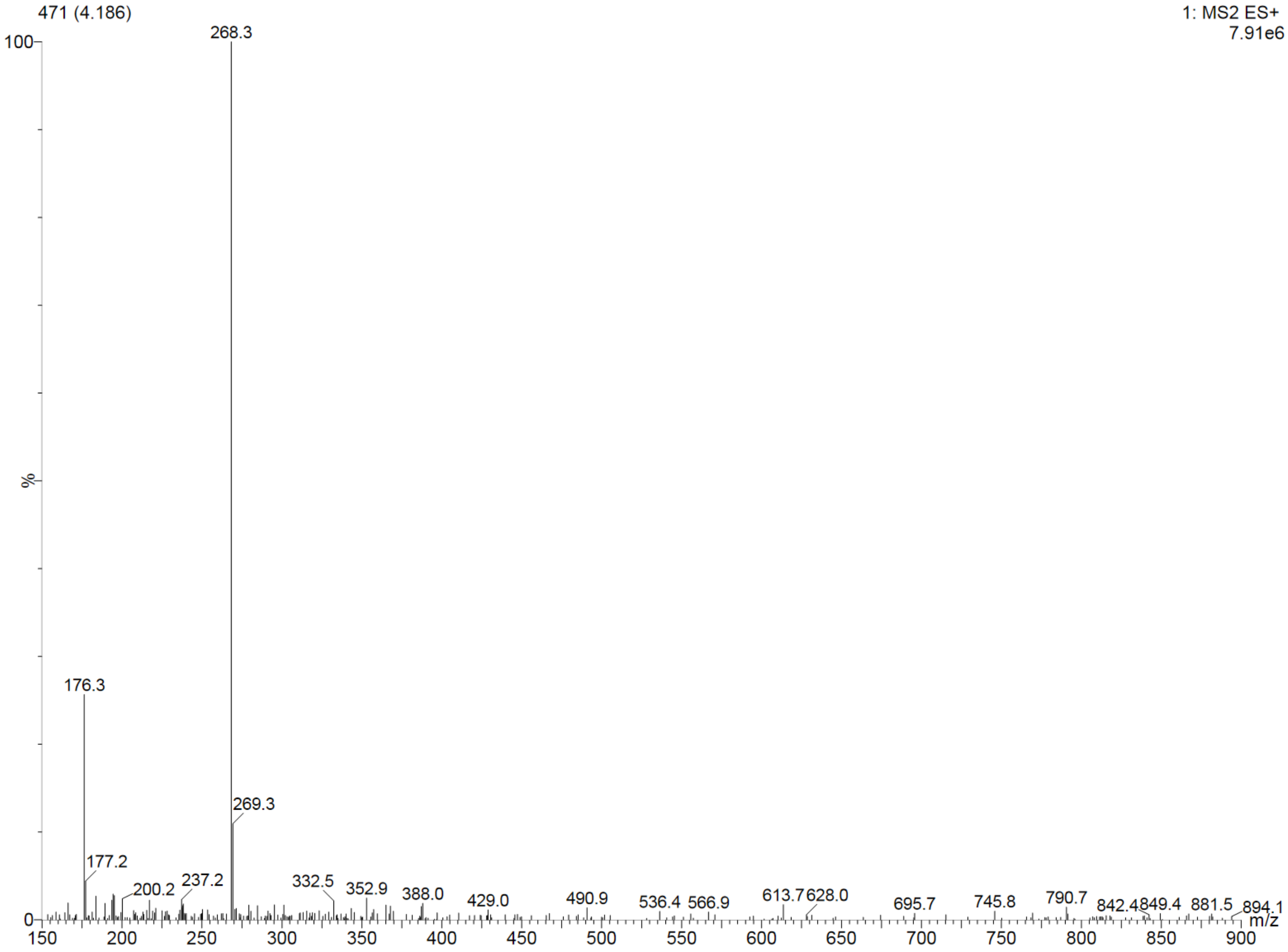
471 (4.186)
1: MS2 ES+
7.91e6
100
%
0
268.3
176.3
269.3
177.2
200.2
237.2
332.5
352.9
388.0
429.0
490.9
536.4
566.9
613.7
628.0
695.7
745.8
790.7
842.4
849.4
881.5
894.1
m/z
150
200
250
300
350
400
450
500
550
600
650
700
750
800
850
900

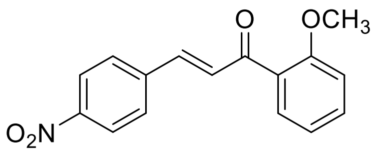
O
OCH3
O2N

5a

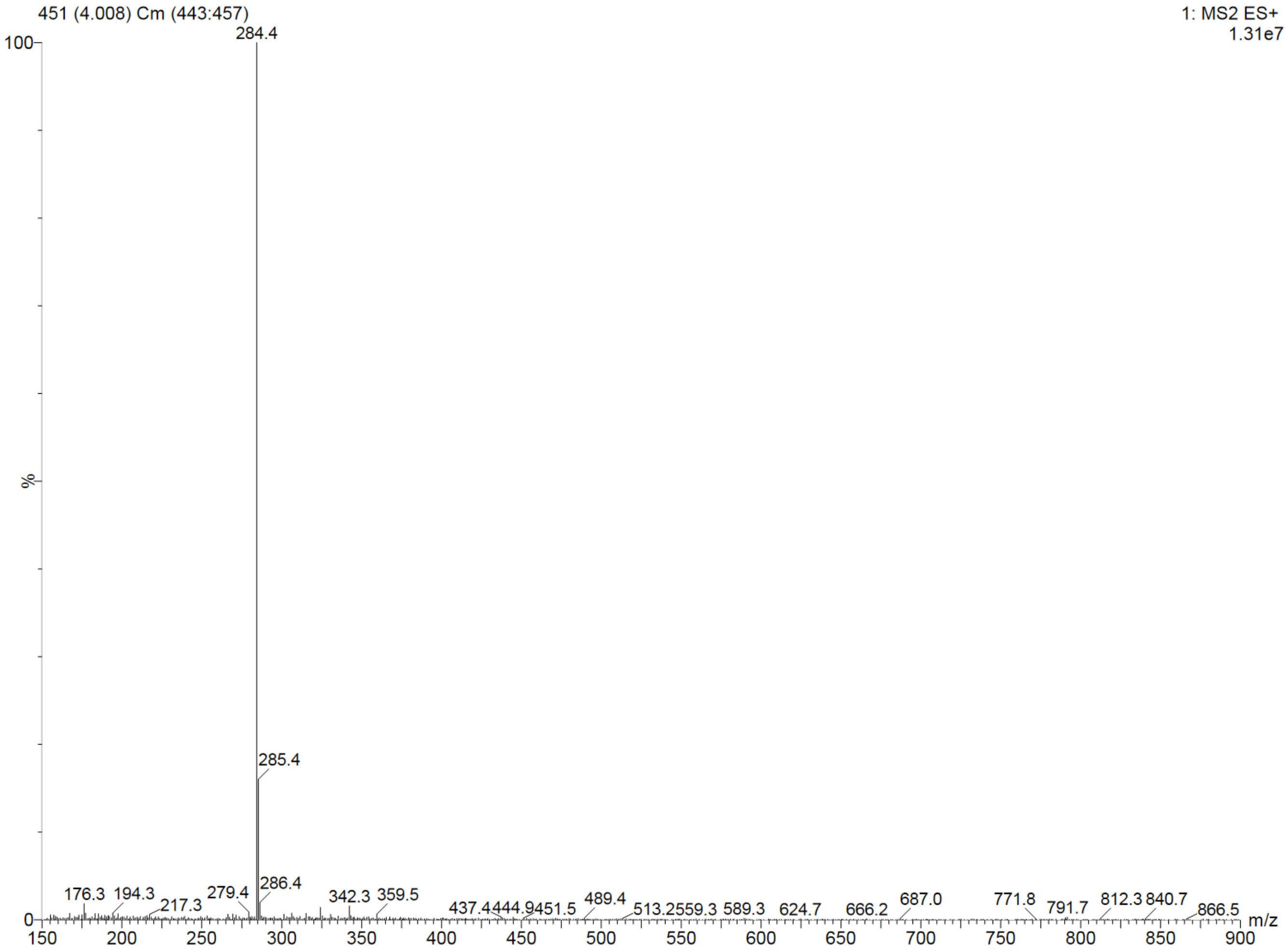
451 (4.008) Cm (443:457)
1: MS2 ES+
1.31e7
100
%
0
284.4
285.4
286.4
176.3
194.3
217.3
279.4
342.3
359.5
437.4
444.9
451.5
489.4
513.2
559.3
589.3
624.7
666.2
687.0
771.8
791.7
812.3
840.7
866.5
m/z
150
200
250
300
350
400
450
500
550
600
650
700
750
800
850
900

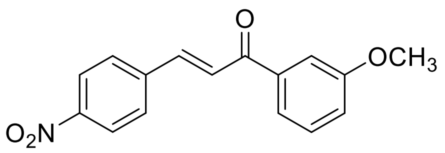
O
OCH3
O2N

6a

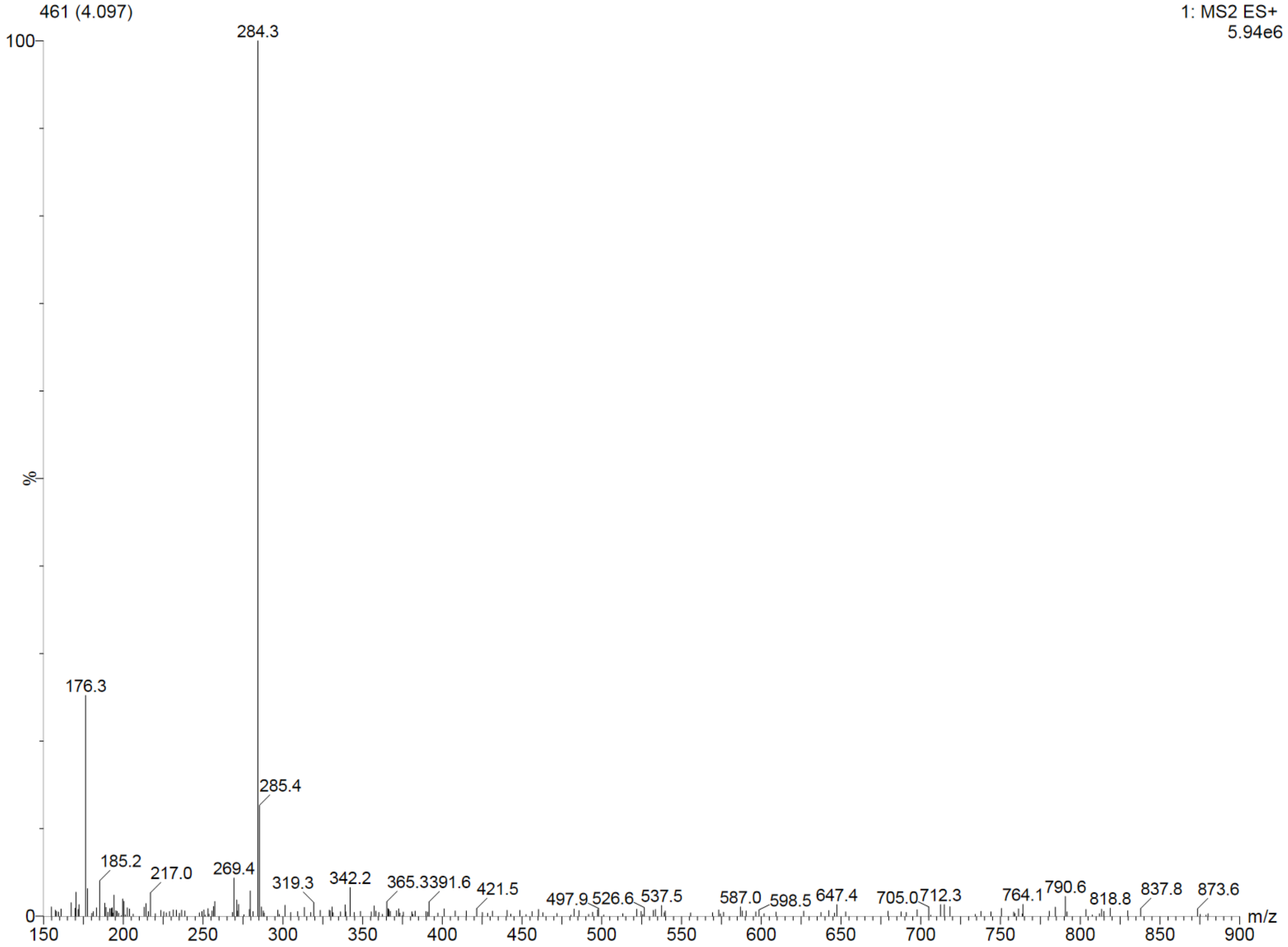
461 (4.097)
1: MS2 ES+
5.94e6
100
%
0
284.3
176.3
285.4
185.2
217.0
269.4
319.3
342.2
365.3391.6
421.5
497.9
526.6
537.5
587.0
598.5
647.4
705.0712.3
764.1
790.6
818.8
837.8
873.6
m/z
150
200
250
300
350
400
450
500
550
600
650
700
750
800
850
900

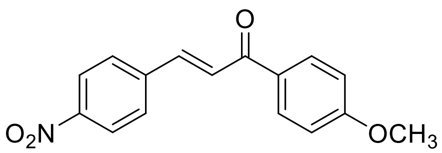
O
O2N
OCH3

7a

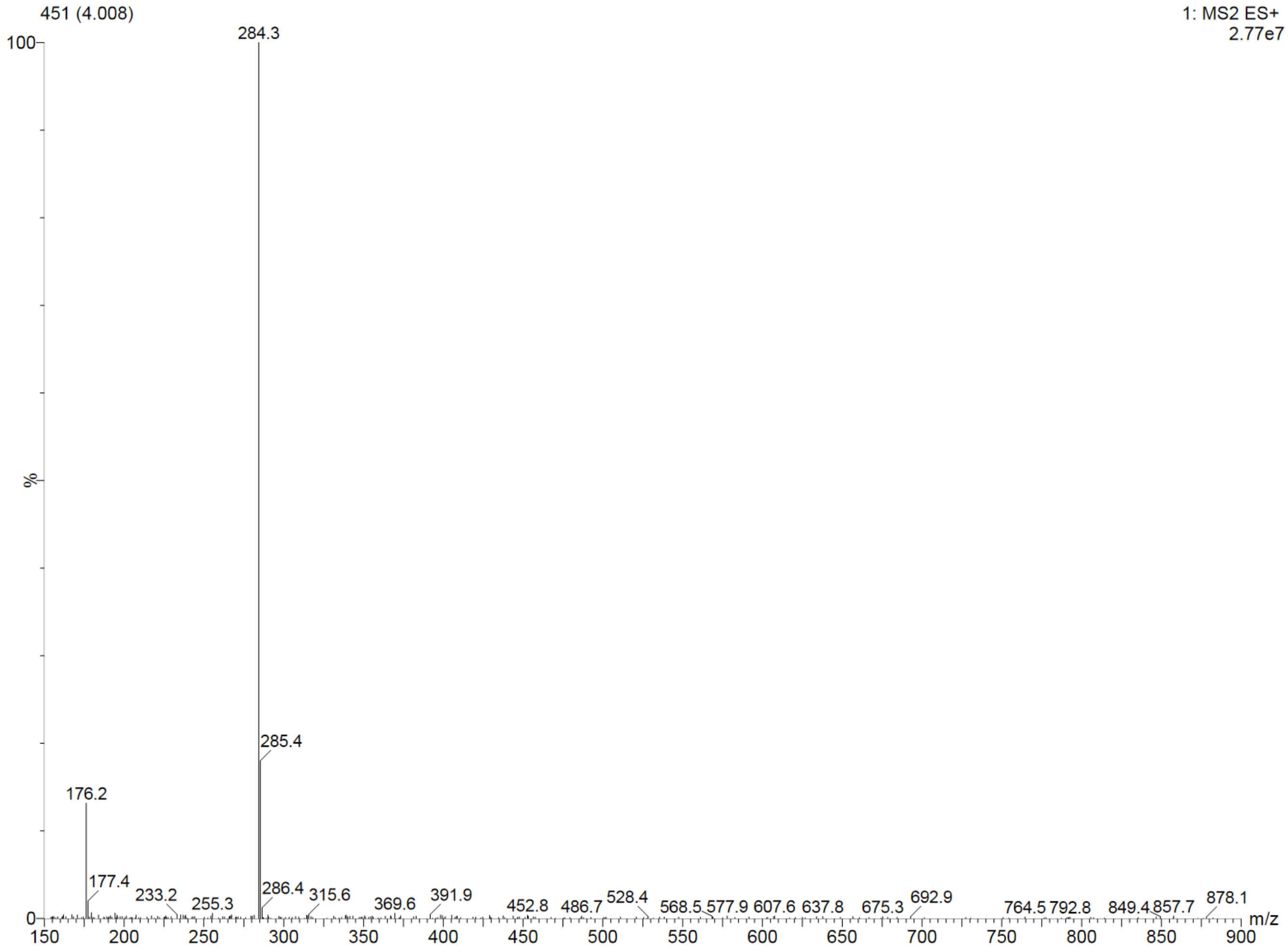
451 (4.008)
1: MS2 ES+
2.77e7
284.3
285.4
176.2
177.4
233.2
255.3
286.4
315.6
369.6
391.9
452.8
486.7
528.4
568.5
577.9
607.6
637.8
675.3
692.9
764.5
792.8
849.4
857.7
878.1
100
%
0
m/z
150
200
250
300
350
400
450
500
550
600
650
700
750
800
850
900

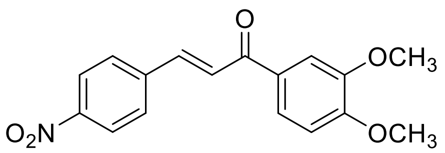
O
OCH3
OCH3
O2N

8a

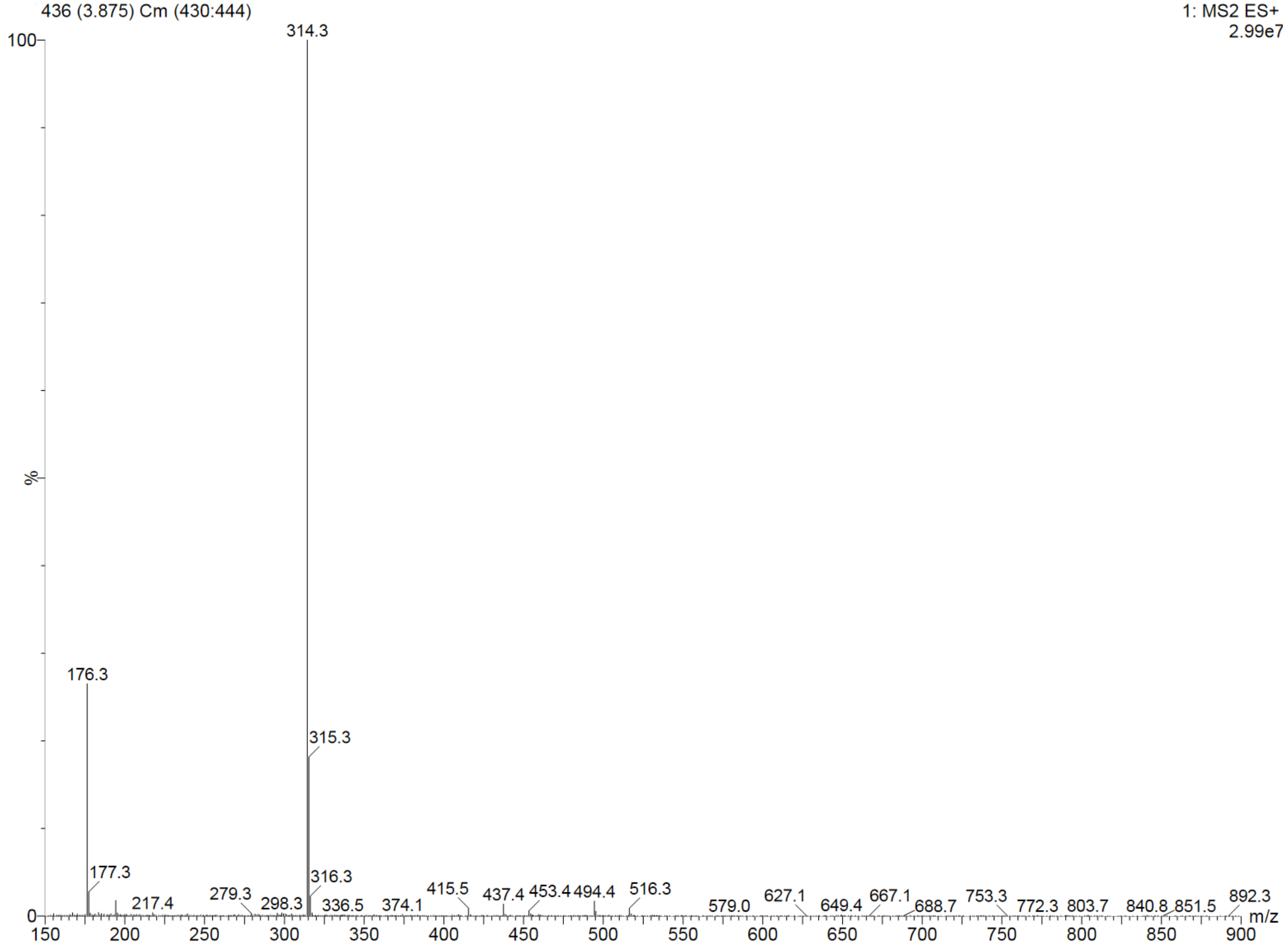
436 (3.875) Cm (430:444)
1: MS2 ES+
2.99e7
314.3
176.3
315.3
177.3
316.3
217.4
279.3
298.3
336.5
374.1
415.5
437.4
453.4
494.4
516.3
579.0
627.1
649.4
667.1
688.7
753.3
772.3
803.7
840.8
851.5
892.3
%
m/z
100
0
150
200
250
300
350
400
450
500
550
600
650
700
750
800
850
900

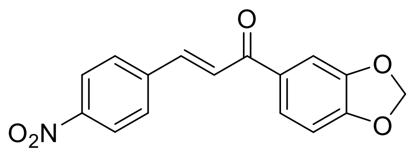
O
O
O
O_2N

9a

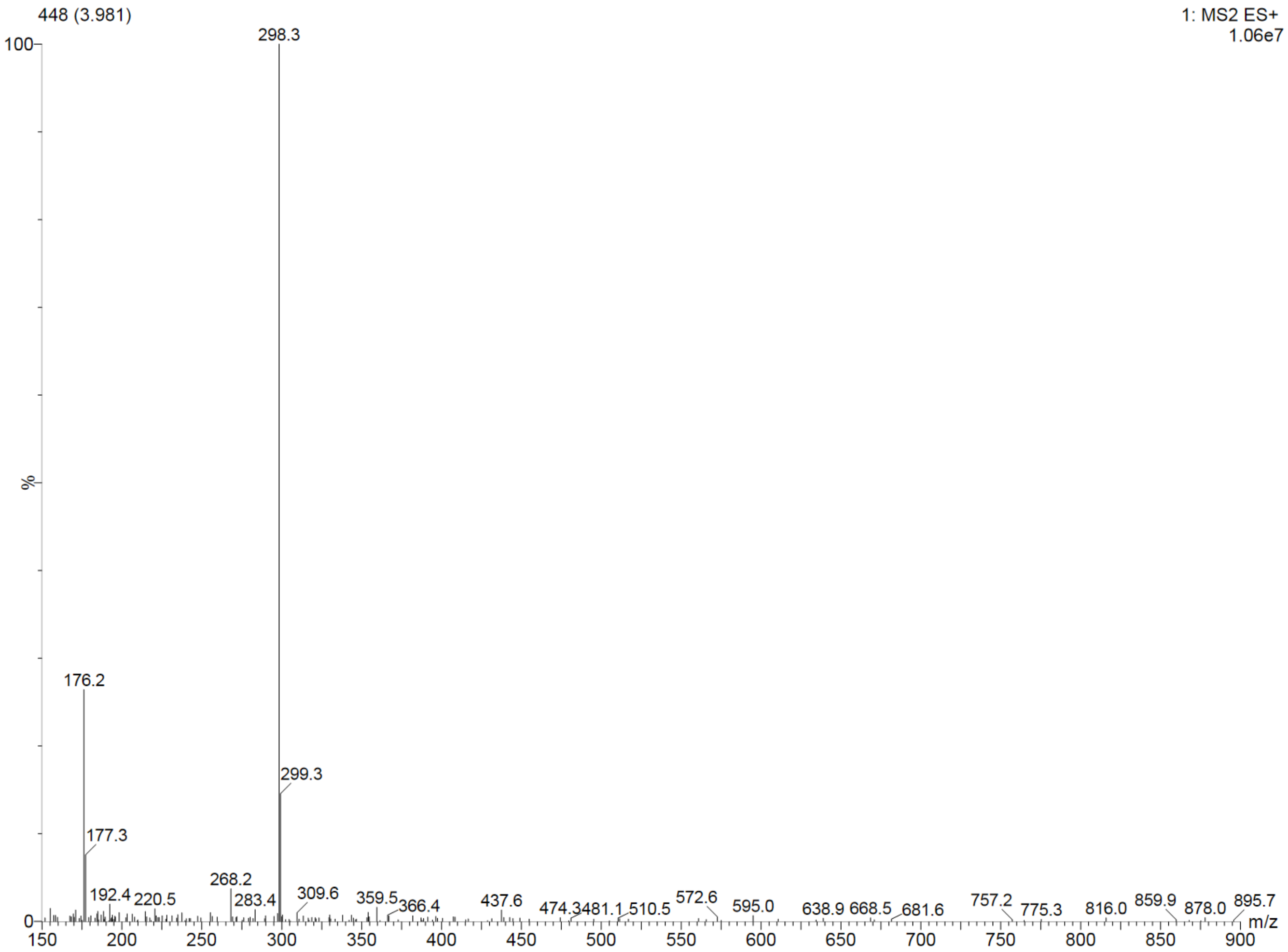
448 (3.981)
1: MS2 ES+
1.06e7
100
%
0
298.3
176.2
299.3
177.3
192.4
220.5
268.2
283.4
309.6
359.5
366.4
437.6
474.3
481.1
510.5
572.6
595.0
638.9
668.5
681.6
757.2
775.3
816.0
859.9
878.0
895.7
m/z
150
200
250
300
350
400
450
500
550
600
650
700
750
800
850
900

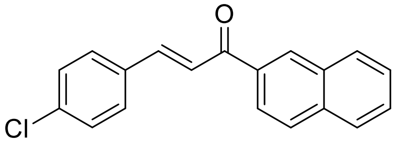
O
Cl

10a

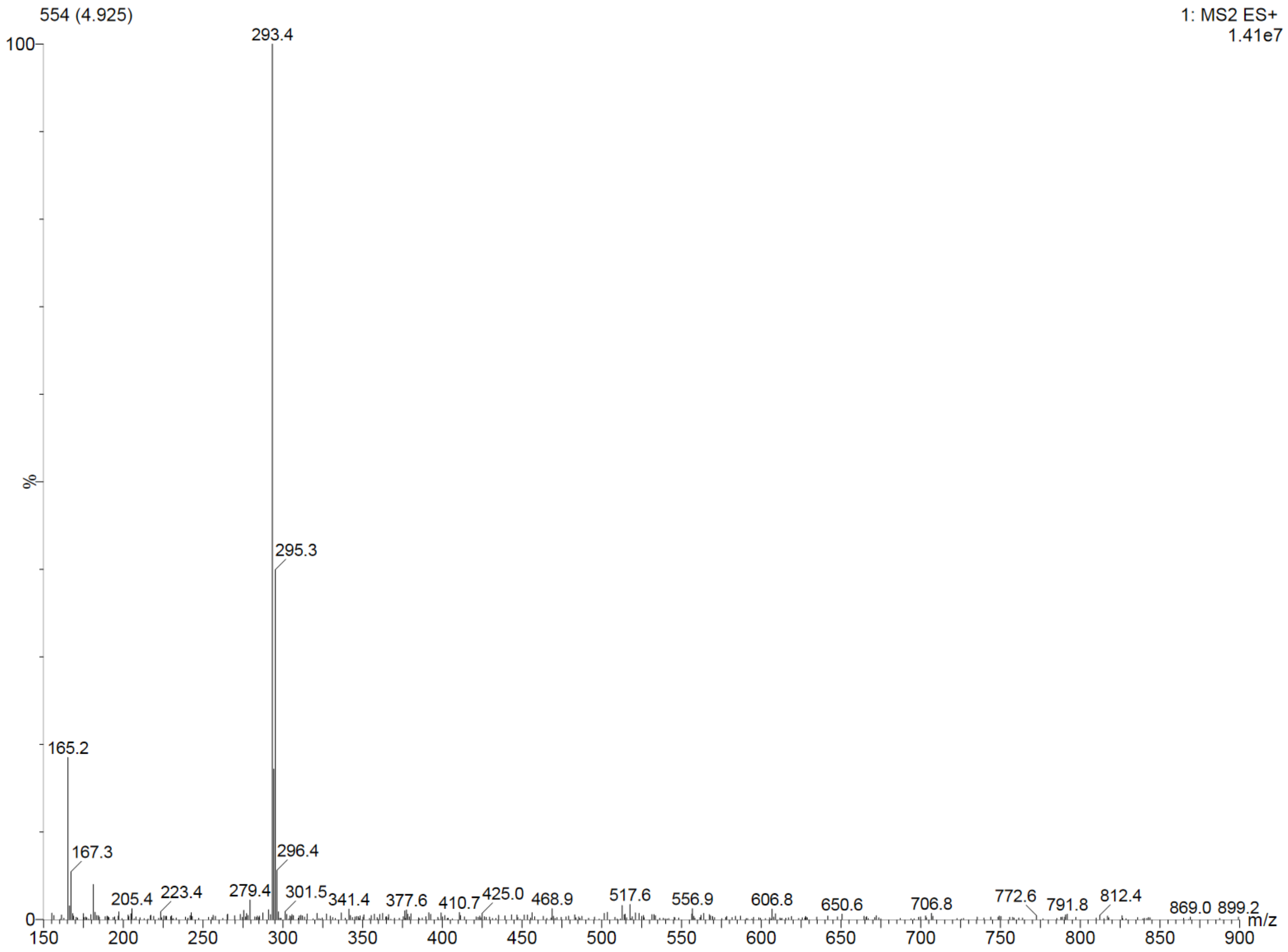
554 (4.925)
1: MS2 ES+
1.41e7
100
%
0
293.4
295.3
165.2
167.3
296.4
205.4
223.4
279.4
301.5
341.4
377.6
410.7
425.0
468.9
517.6
556.9
606.8
650.6
706.8
772.6
791.8
812.4
869.0
899.2
m/z
150
200
250
300
350
400
450
500
550
600
650
700
750
800
850
900

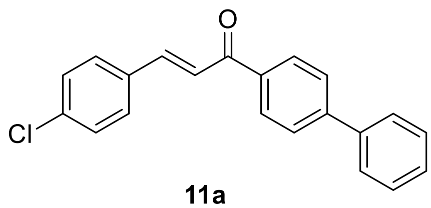
O
Cl

11a

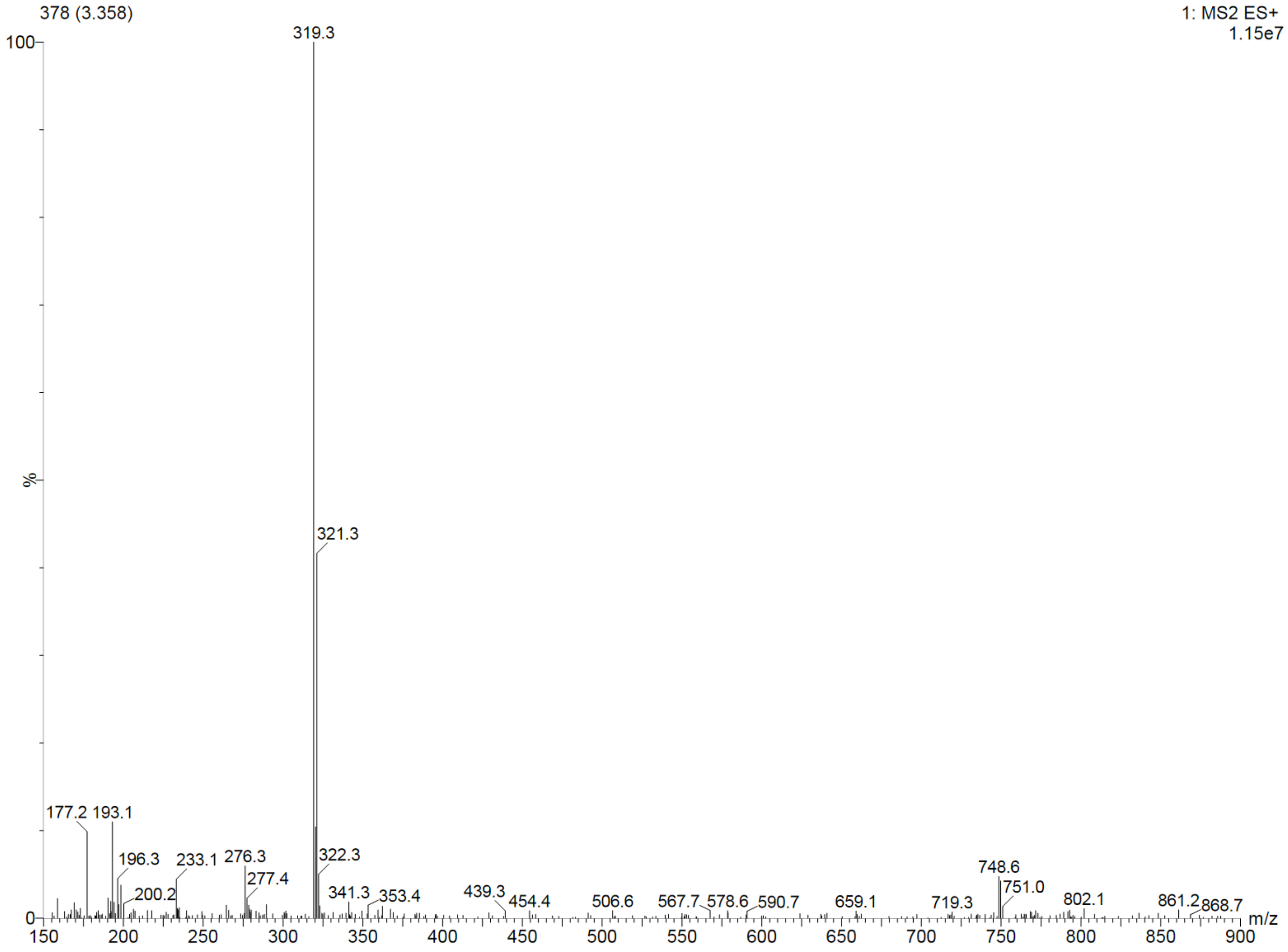
378 (3.358)
1: MS2 ES+
1.15e7
100
%
0
319.3
321.3
177.2 193.1
196.3
233.1
276.3
277.4
200.2
322.3
341.3
353.4
439.3
454.4
506.6
567.7
578.6
590.7
659.1
719.3
748.6
751.0
802.1
861.2
868.7
m/z
150
200
250
300
350
400
450
500
550
600
650
700
750
800
850
900

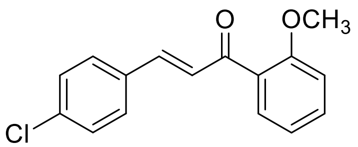
O
OCH3
Cl

12a

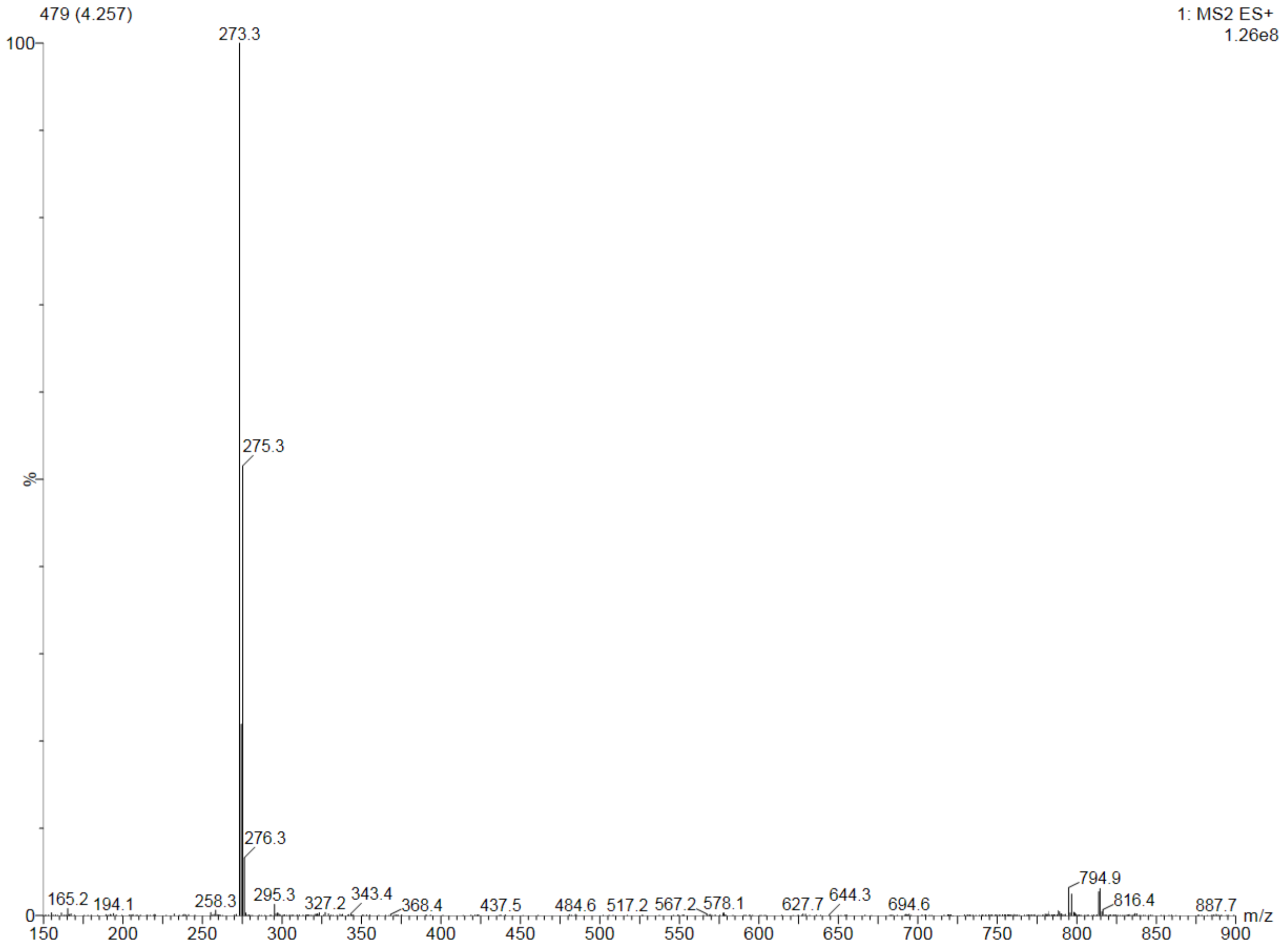
479 (4.257)
1: MS2 ES+
1.26e8
100
%
0
273.3
275.3
276.3
165.2
194.1
258.3
295.3
327.2
343.4
368.4
437.5
484.6
517.2
567.2
578.1
627.7
644.3
694.6
794.9
816.4
887.7
m/z
150
200
250
300
350
400
450
500
550
600
650
700
750
800
850
900

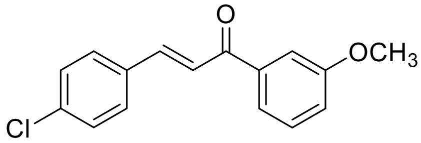
O
OCH3
Cl

13a

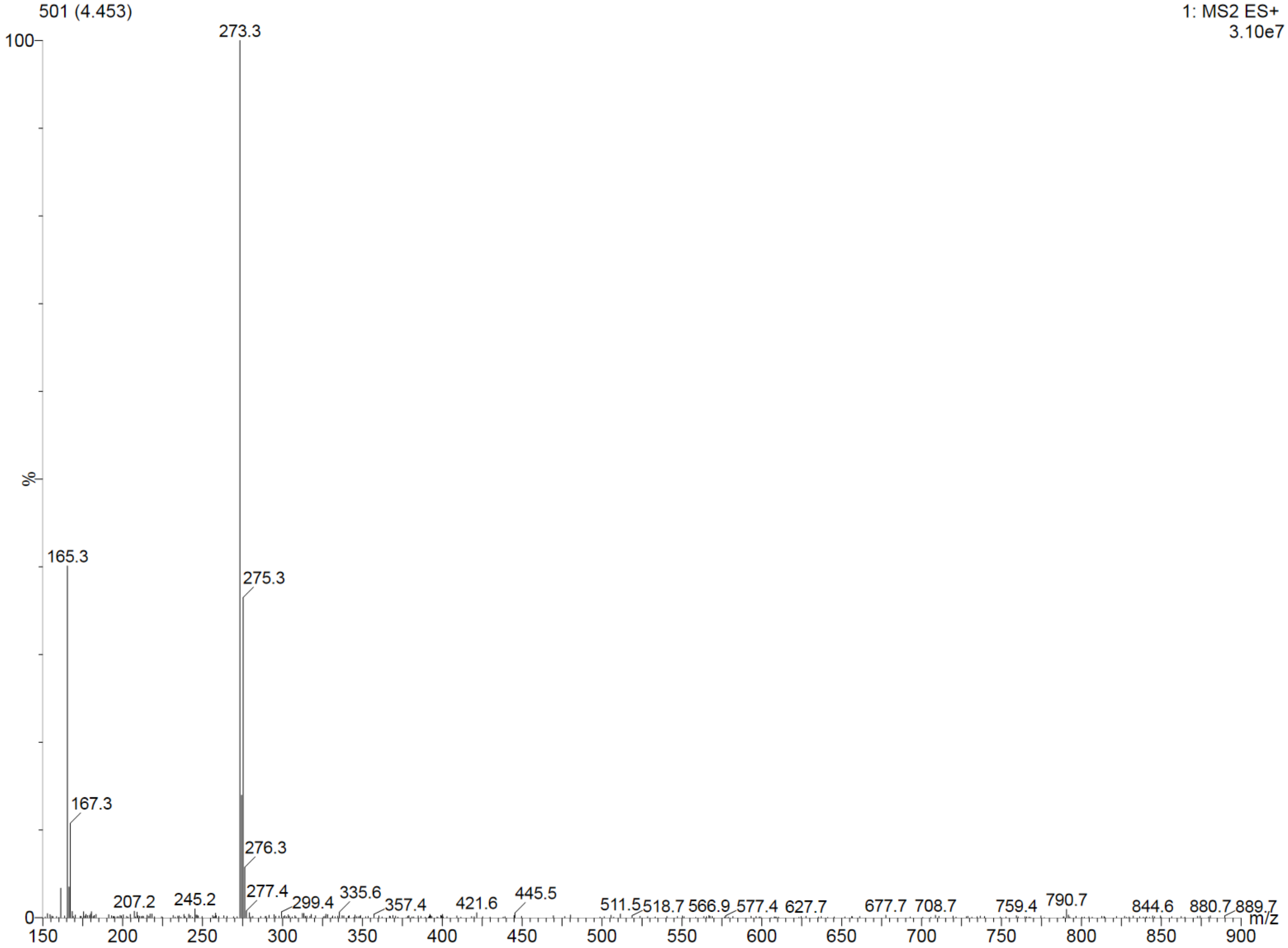
501 (4.453)
1: MS2 ES+
3.10e7
273.3
165.3
275.3
167.3
276.3
207.2
245.2
277.4
299.4
335.6
357.4
421.6
445.5
511.5
518.7
566.9
577.4
627.7
677.7
708.7
759.4
790.7
844.6
880.7
889.7
%
m/z
100
0
150
200
250
300
350
400
450
500
550
600
650
700
750
800
850
900

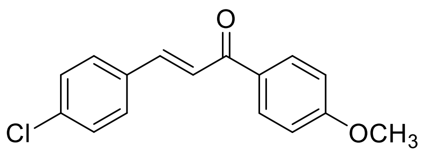

O
Cl
OCH3

14a

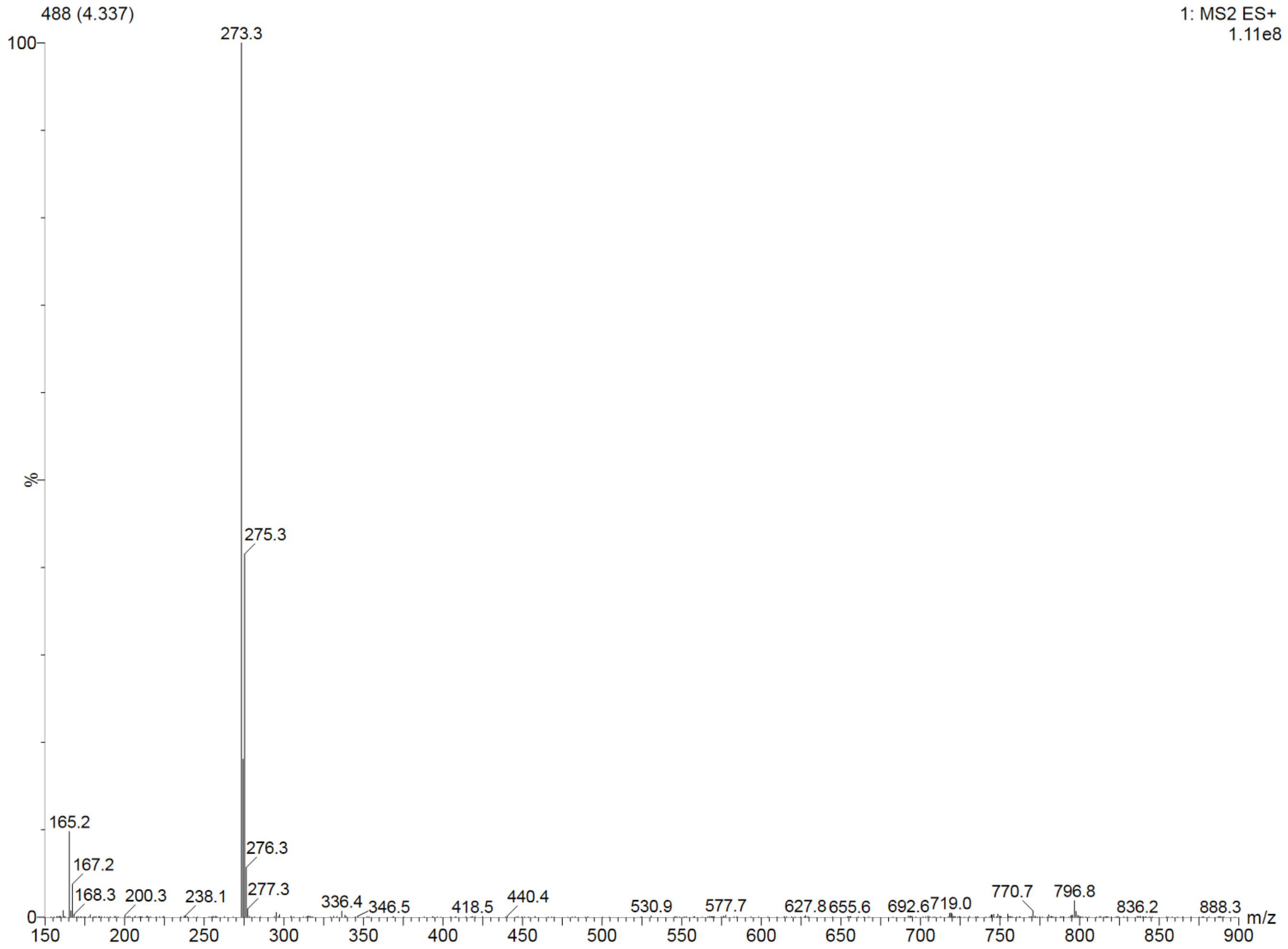

488 (4.337)
1: MS2 ES+
1.11e8
100
%
0
273.3
275.3
165.2
167.2
168.3
200.3
238.1
276.3
277.3
336.4
346.5
418.5
440.4
530.9
577.7
627.8
655.6
692.6
719.0
770.7
796.8
836.2
888.3
m/z
150
200
250
300
350
400
450
500
550
600
650
700
750
800
850
900

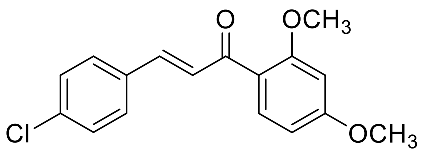
O
OCH3
OCH3
Cl

15a

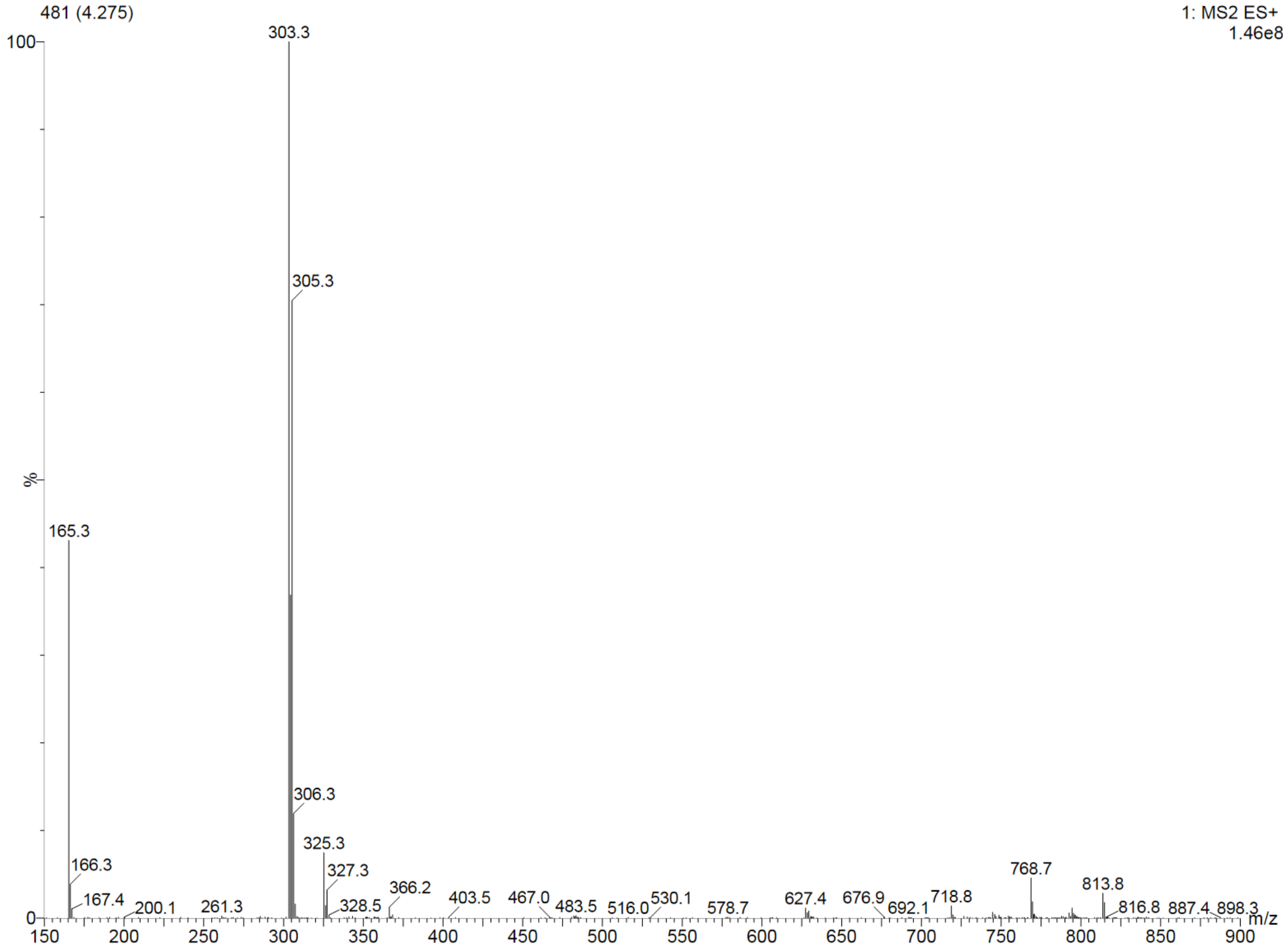
481 (4.275)
1: MS2 ES+
1.46e8
100
%
0
303.3
305.3
165.3
306.3
325.3
166.3
327.3
167.4
200.1
261.3
328.5
366.2
403.5
467.0
483.5
516.0
530.1
578.7
627.4
676.9
692.1
718.8
768.7
813.8
816.8
887.4
898.3
m/z
150
200
250
300
350
400
450
500
550
600
650
700
750
800
850
900

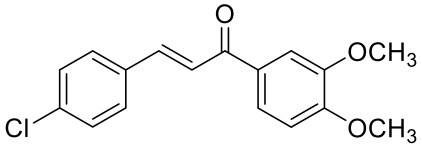
O
OCH3
OCH3
Cl

16a

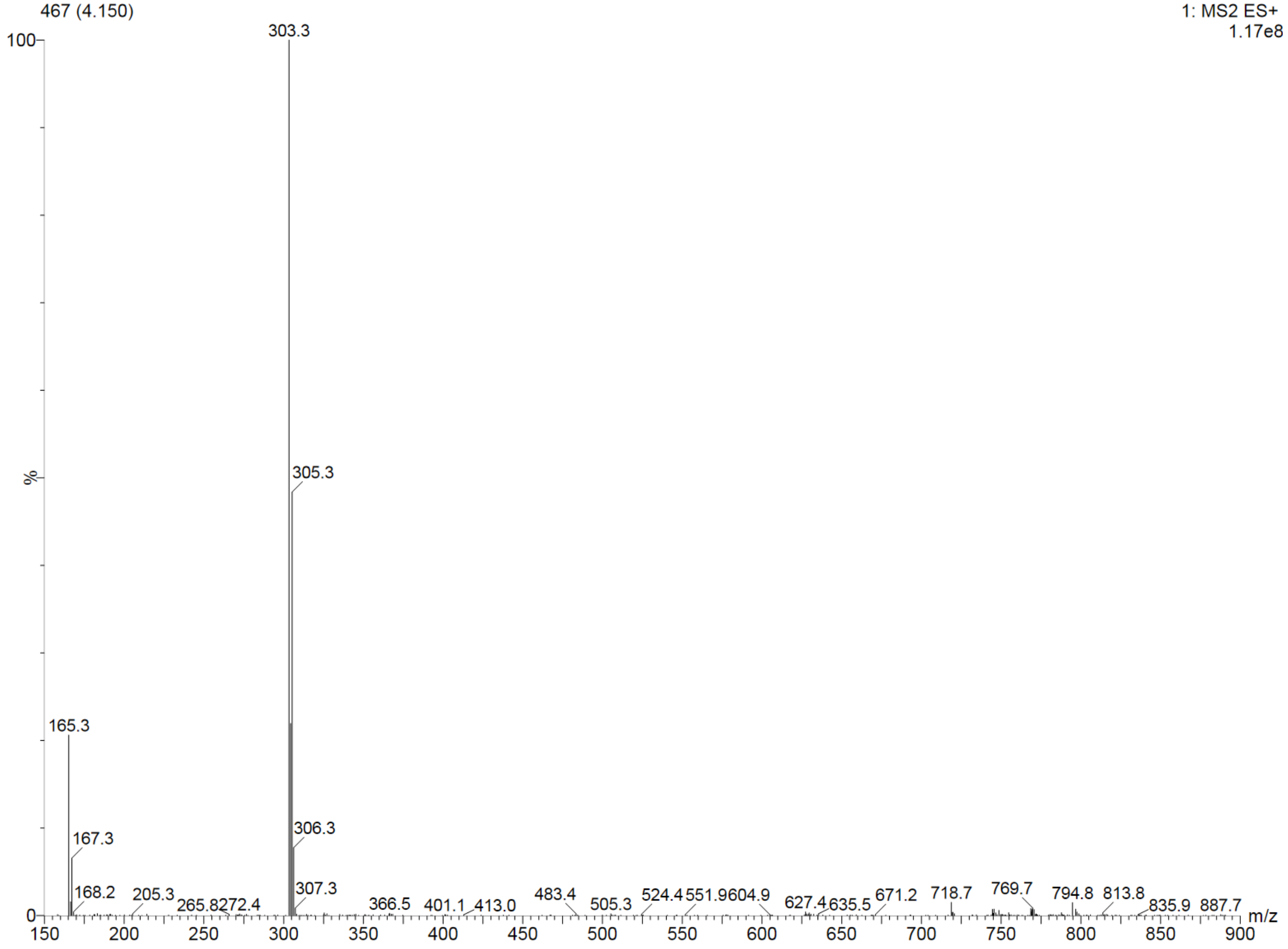
467 (4.150)
1: MS2 ES+
1.17e8
100
%
0
303.3
305.3
165.3
167.3
306.3
168.2
205.3
265.8272.4
307.3
366.5
401.1
413.0
483.4
505.3
524.4
551.9604.9
627.4
635.5
671.2
718.7
769.7
794.8
813.8
835.9
887.7
m/z
150
200
250
300
350
400
450
500
550
600
650
700
750
800
850
900

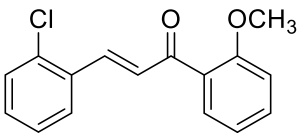

Cl
O
OCH3

17a

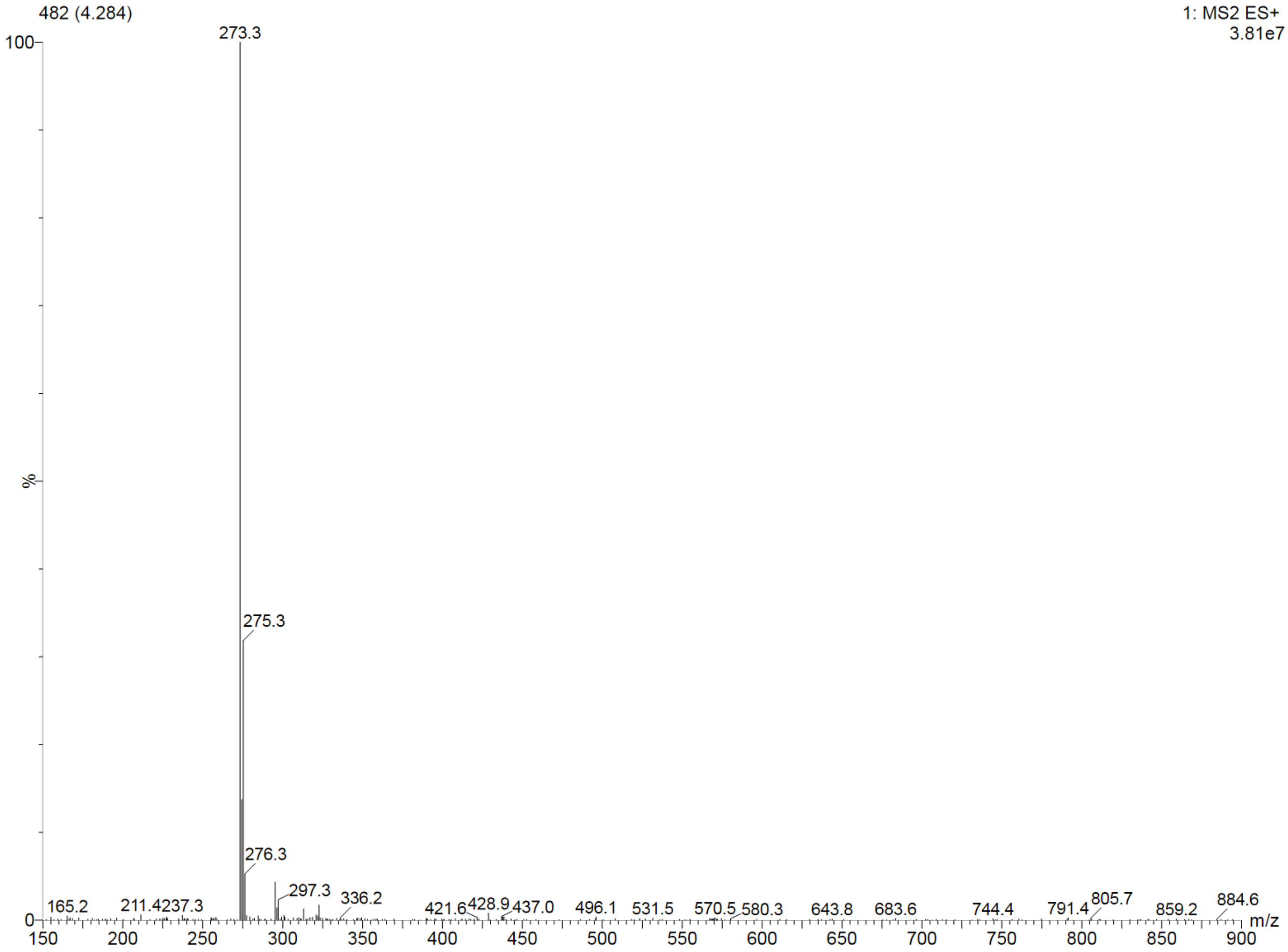

482 (4.284)
1: MS2 ES+
3.81e7
273.3
275.3
276.3
297.3
336.2
165.2
211.4237.3
421.6
428.9
437.0
496.1
531.5
570.5
580.3
643.8
683.6
744.4
791.4
805.7
859.2
884.6
100
%
0
m/z
150
200
250
300
350
400
450
500
550
600
650
700
750
800
850
900

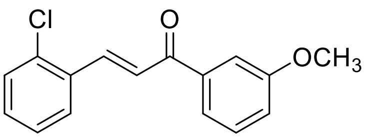
Cl
O
OCH3

18a

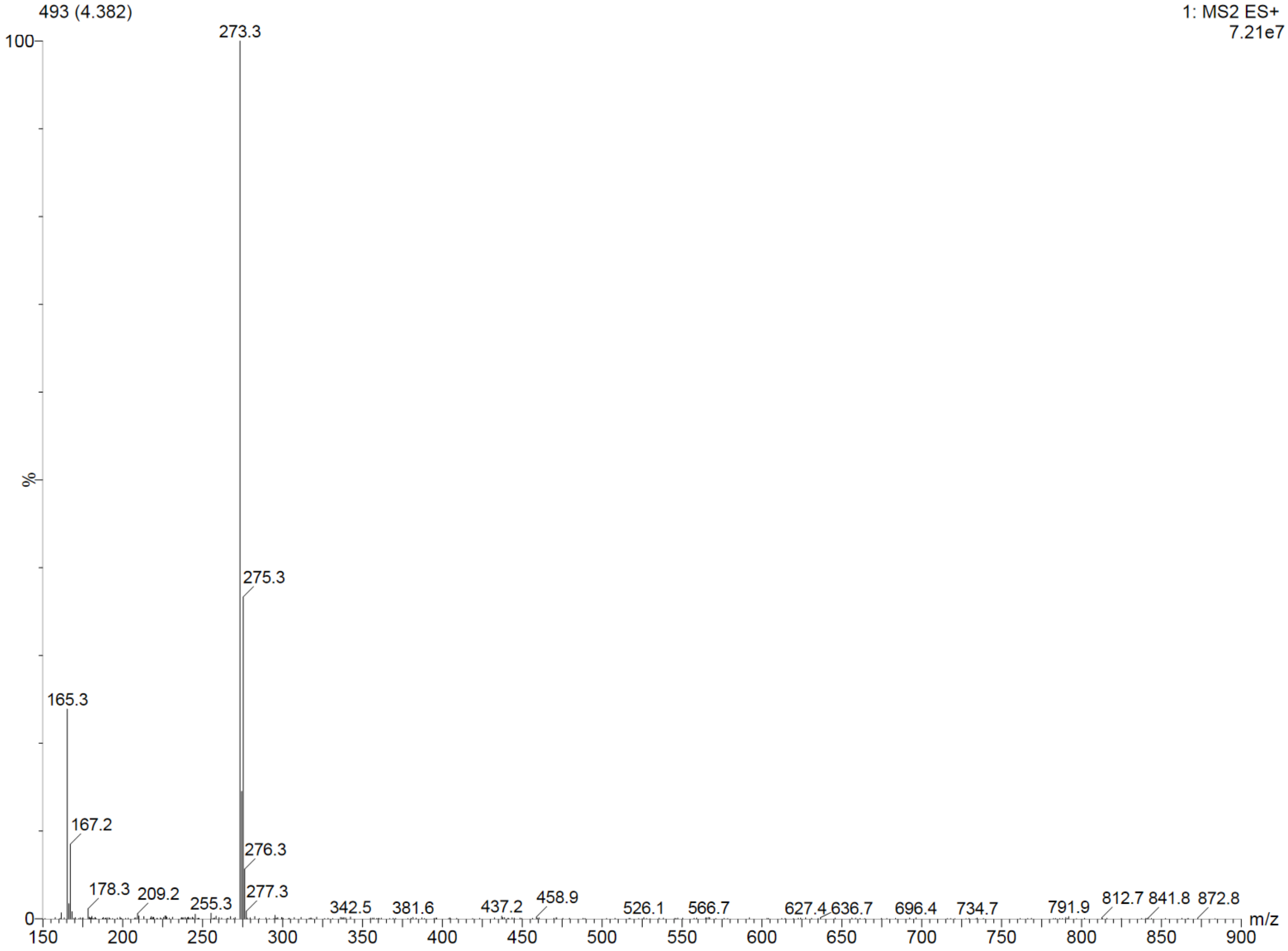
493 (4.382)
1: MS2 ES+
7.21e7
100
%
0
273.3
275.3
165.3
167.2
276.3
178.3
209.2
255.3
277.3
342.5
381.6
437.2
458.9
526.1
566.7
627.4
636.7
696.4
734.7
791.9
812.7
841.8
872.8
150
200
250
300
350
400
450
500
550
600
650
700
750
800
850
900
m/z

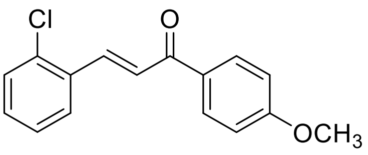
Cl
O
OCH3

19a

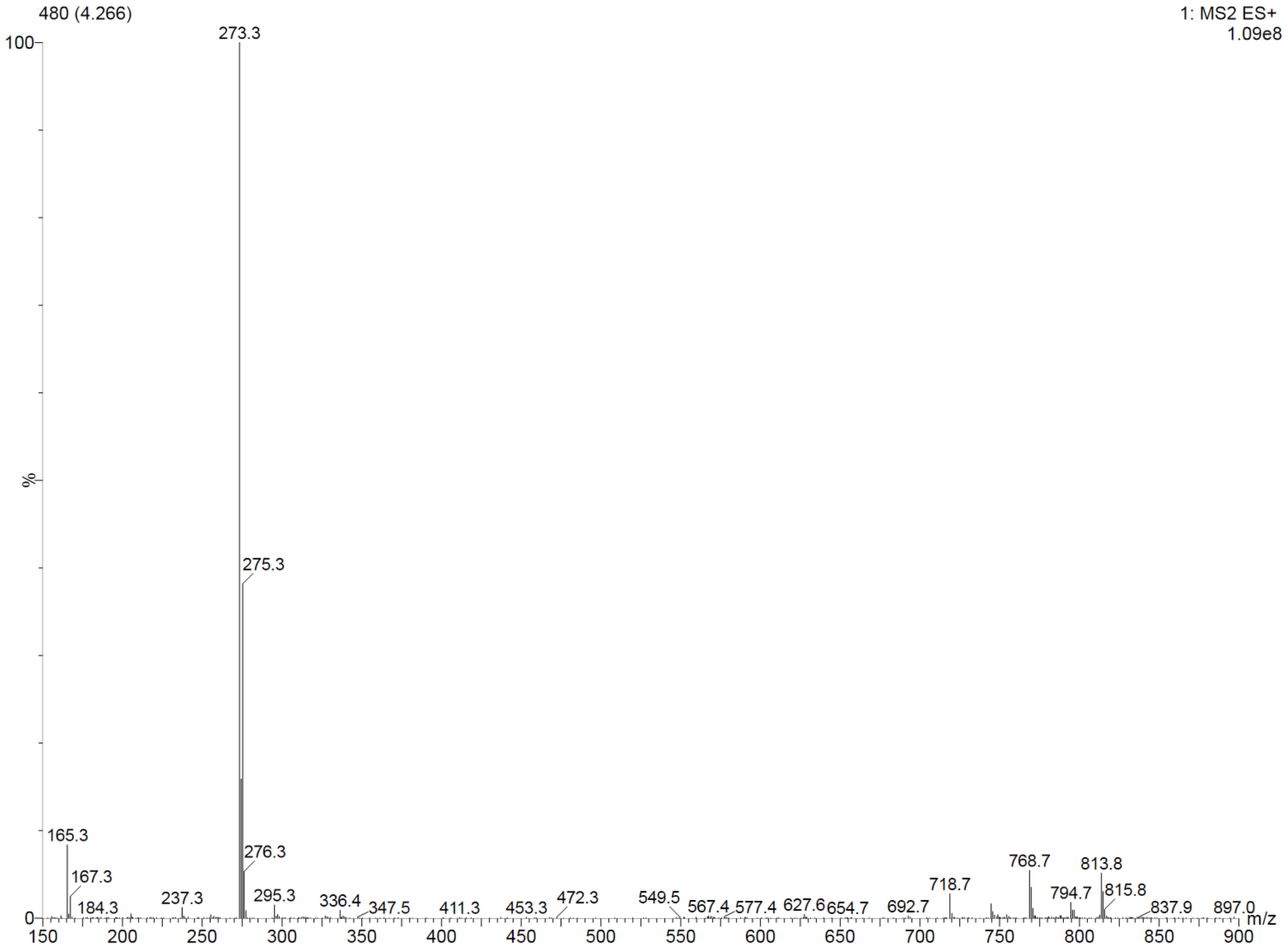
480 (4.266)
1: MS2 ES+
1.09e8
100
%
0
273.3
275.3
276.3
165.3
167.3
184.3
237.3
295.3
336.4
347.5
411.3
453.3
472.3
549.5
567.4
577.4
627.6
654.7
692.7
718.7
768.7
794.7
813.8
815.8
837.9
897.0
m/z
150
200
250
300
350
400
450
500
550
600
650
700
750
800
850
900

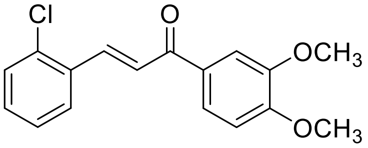
Cl
O
OCH3
OCH3

20a

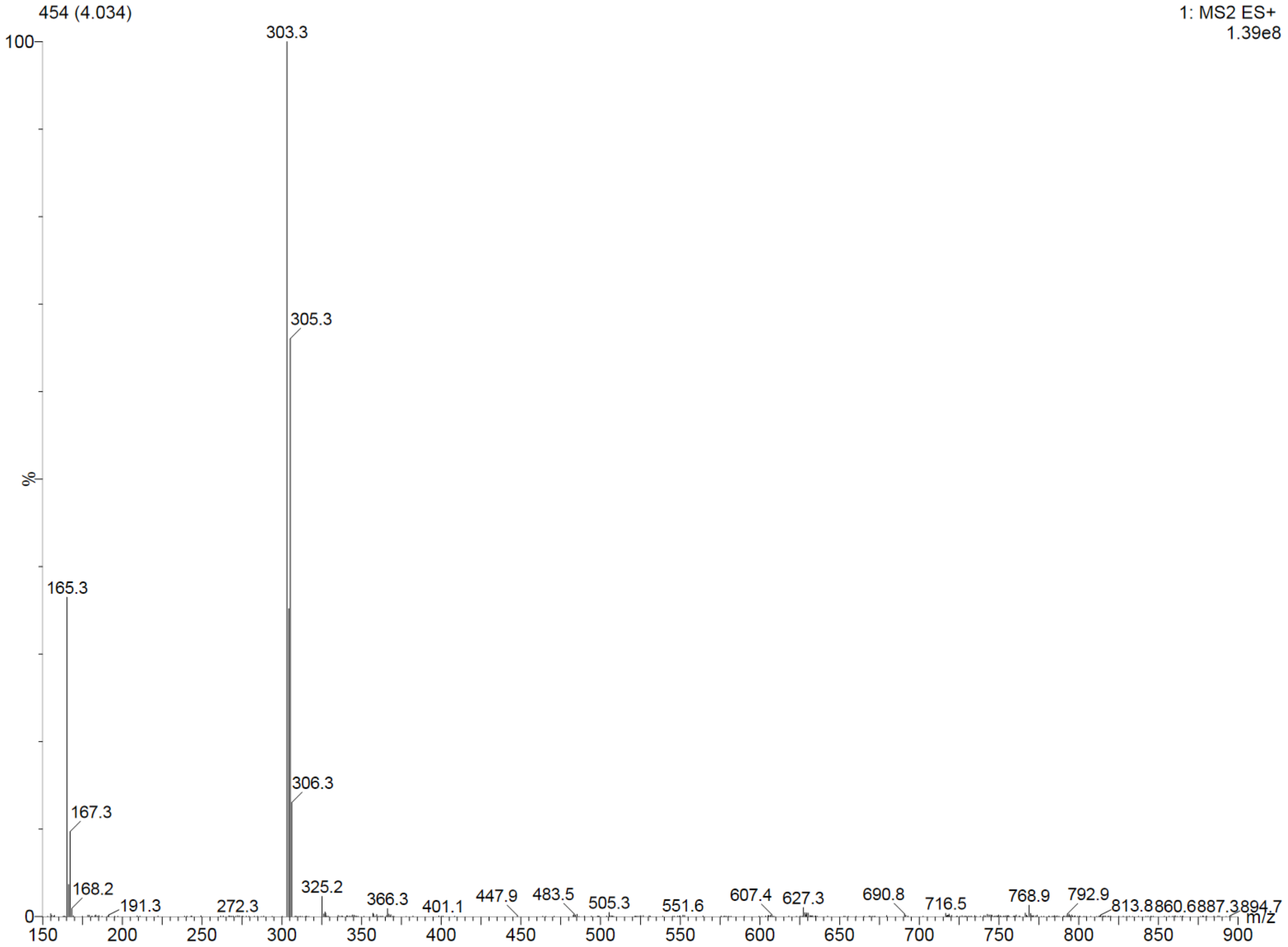
454 (4.034)
1: MS2 ES+
1.39e8
100
%
0
303.3
305.3
165.3
167.3
168.2
191.3
272.3
306.3
325.2
366.3
401.1
447.9
483.5
505.3
551.6
607.4
627.3
690.8
716.5
768.9
792.9
813.8
860.6
887.3
894.7
m/z
150
200
250
300
350
400
450
500
550
600
650
700
750
800
850
900

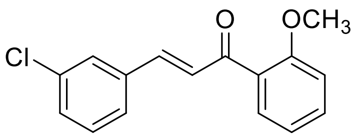

Cl
O
OCH3

21a

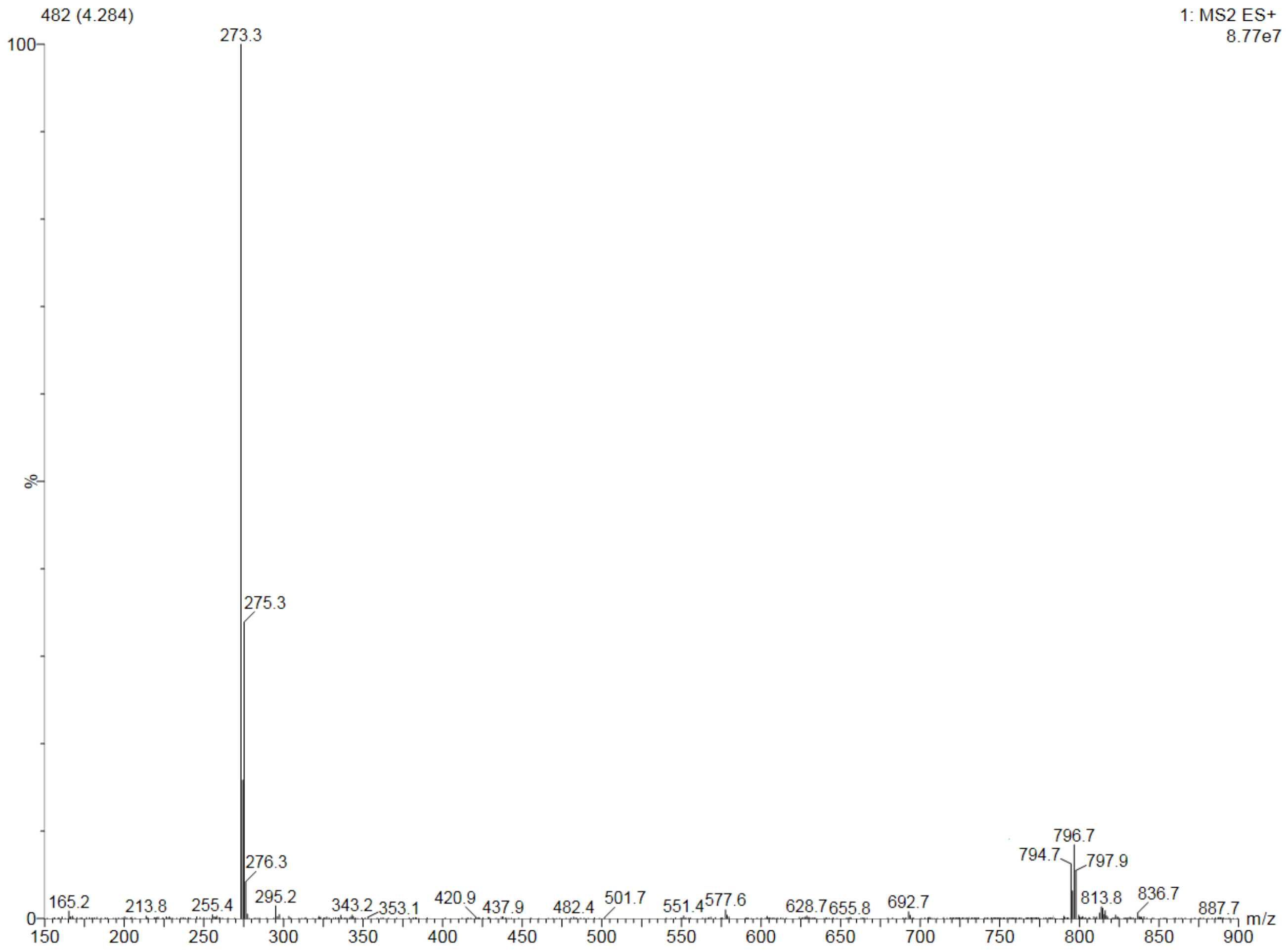

482 (4.284)
1: MS2 ES+
8.77e7
100
%
0
273.3
275.3
276.3
295.2
165.2
213.8
255.4
343.2
353.1
420.9
437.9
482.4
501.7
551.4
577.6
628.7
655.8
692.7
796.7
794.7
797.9
813.8
836.7
887.7
m/z
150
200
250
300
350
400
450
500
550
600
650
700
750
800
850
900

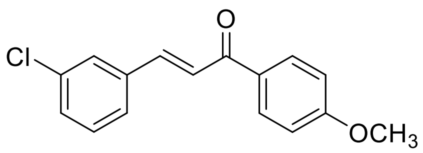
O
Cl
OCH3

22a

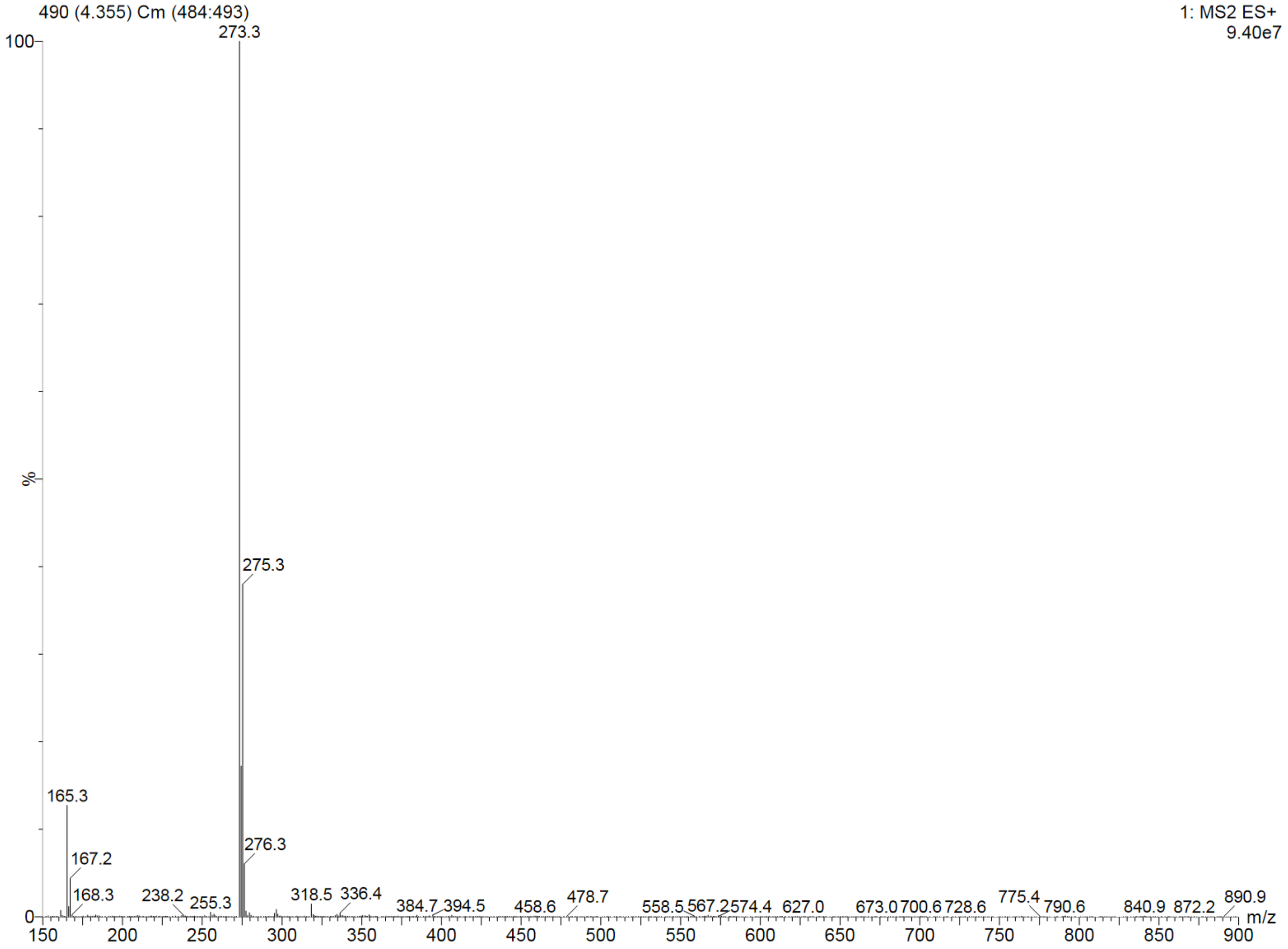
490 (4.355) Cm (484:493)
1: MS2 ES+
9.40e7
100
%
0
273.3
275.3
165.3
276.3
167.2
168.3
238.2
255.3
318.5
336.4
384.7
394.5
458.6
478.7
558.5
567.2
574.4
627.0
673.0
700.6
728.6
775.4
790.6
840.9
872.2
890.9
m/z
150
200
250
300
350
400
450
500
550
600
650
700
750
800
850
900

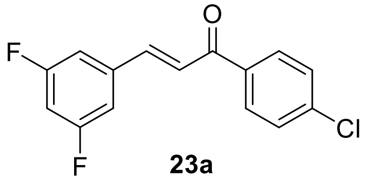
F
O
F
Cl

23a

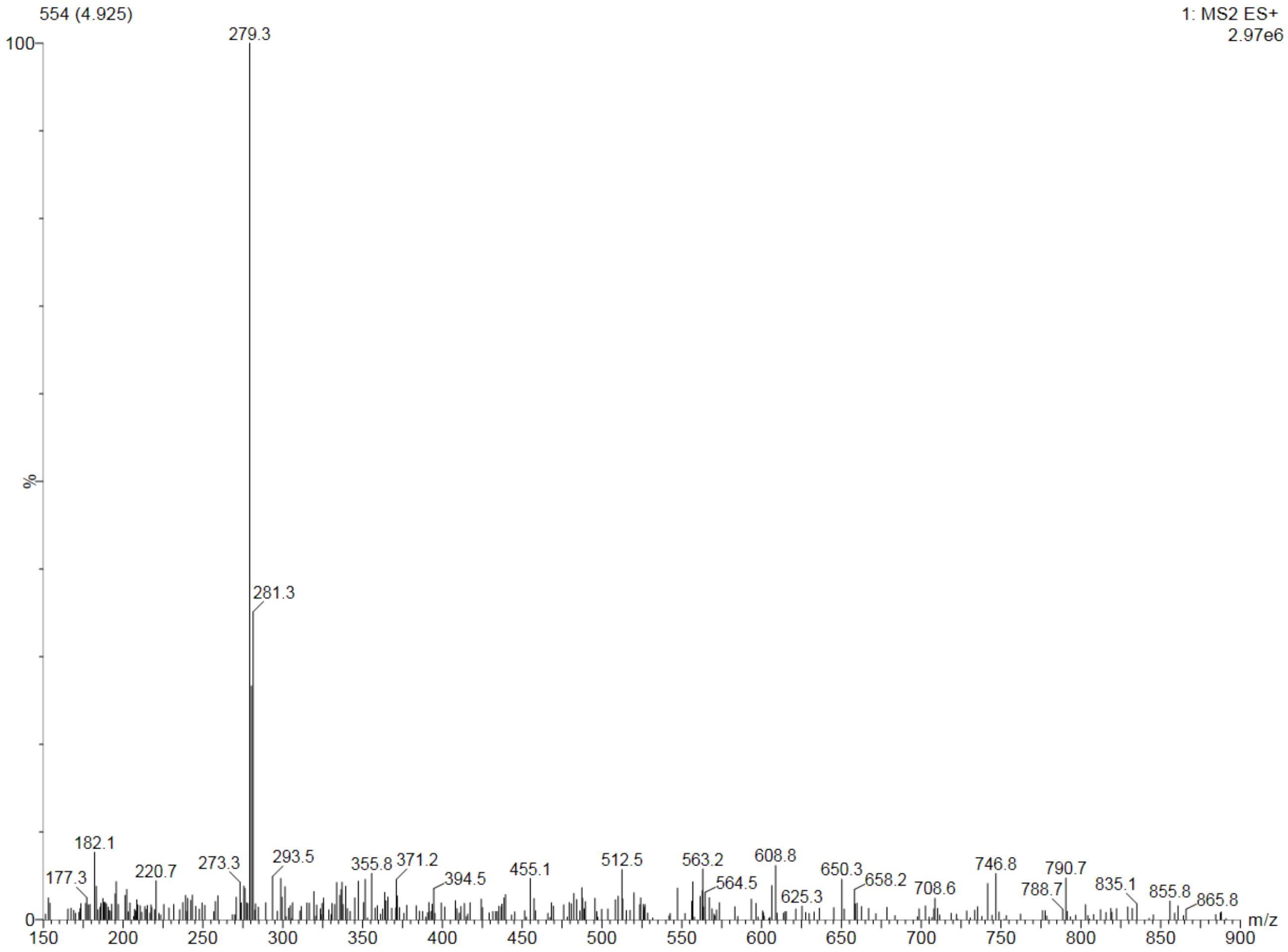
554 (4.925)
1: MS2 ES+
2.97e6
279.3
281.3
182.1
177.3
220.7
273.3
293.5
355.8
371.2
394.5
455.1
512.5
563.2
564.5
608.8
625.3
650.3
658.2
708.6
746.8
788.7
790.7
835.1
855.8
865.8
100
%
0
150
200
250
300
350
400
450
500
550
600
650
700
750
800
850
900
m/z

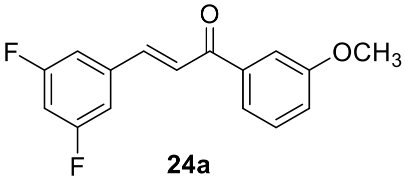
F
F
O
OCH3

24a

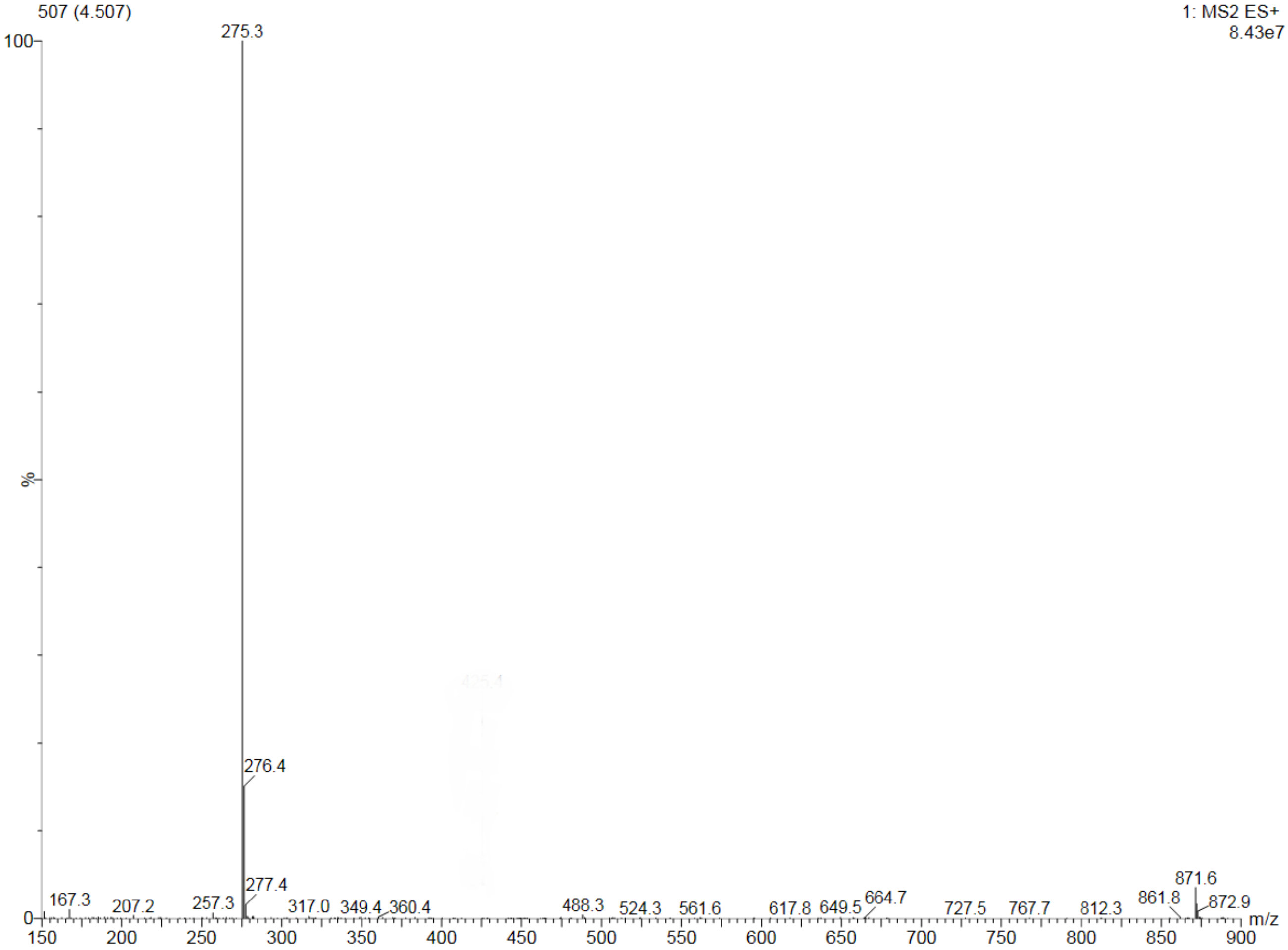
507 (4.507)
1: MS2 ES+
8.43e7
100
%
0
275.3
276.4
277.4
167.3
207.2
257.3
317.0
349.4
360.4
488.3
524.3
561.6
617.8
649.5
664.7
727.5
767.7
812.3
861.8
871.6
872.9
m/z
150
200
250
300
350
400
450
500
550
600
650
700
750
800
850
900

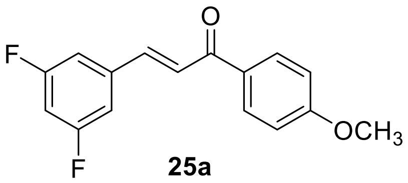
F
F
O
OCH3

25a

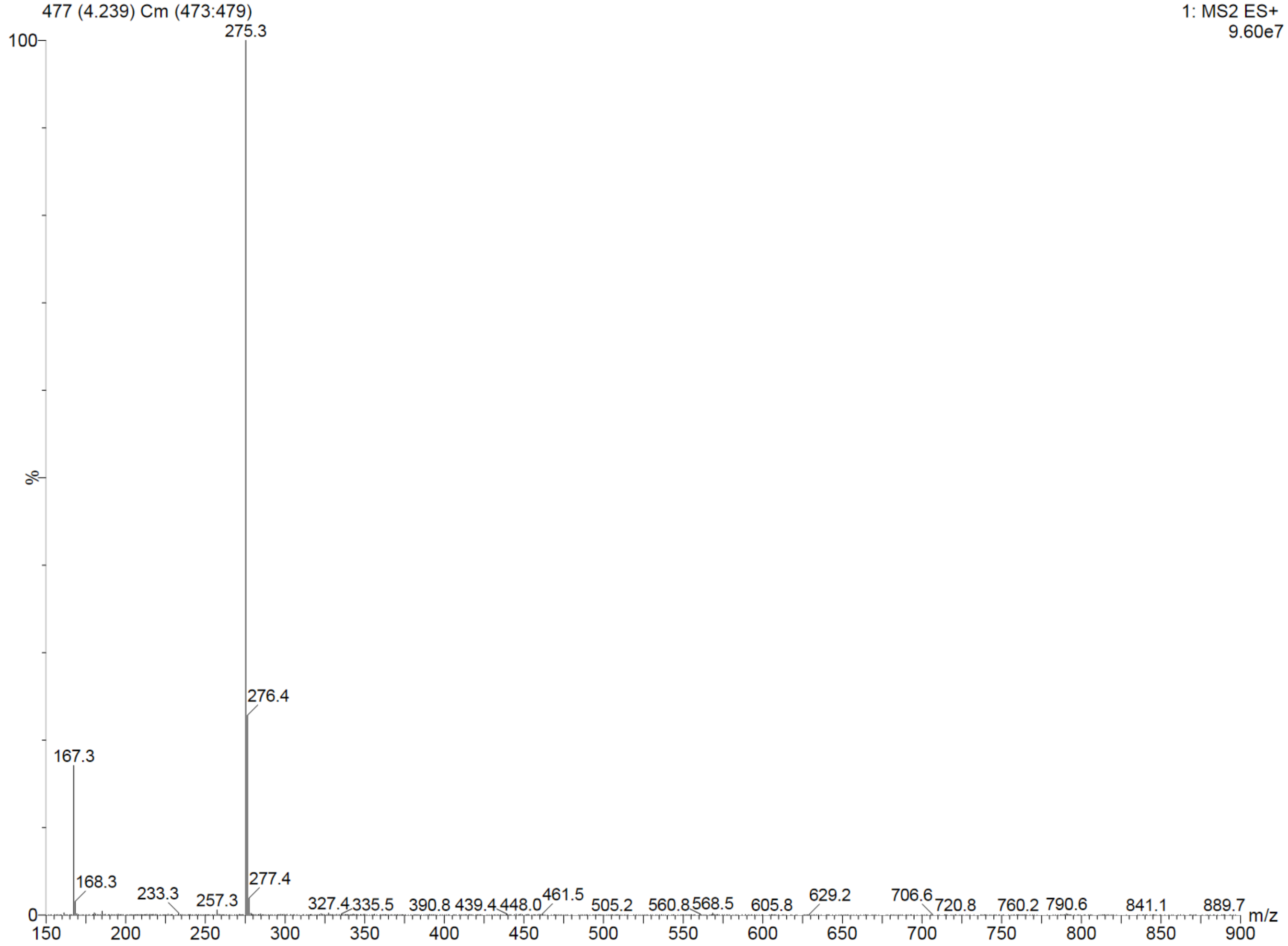
477 (4.239) Cm (473:479)
1: MS2 ES+
9.60e7
100
%
0
275.3
276.4
167.3
168.3
233.3
257.3
277.4
327.4
335.5
390.8
439.4
448.0
461.5
505.2
560.8
568.5
605.8
629.2
706.6
720.8
760.2
790.6
841.1
889.7
m/z
150
200
250
300
350
400
450
500
550
600
650
700
750
800
850
900

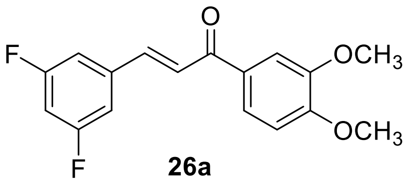
F
F
O
OCH3
OCH3

26a

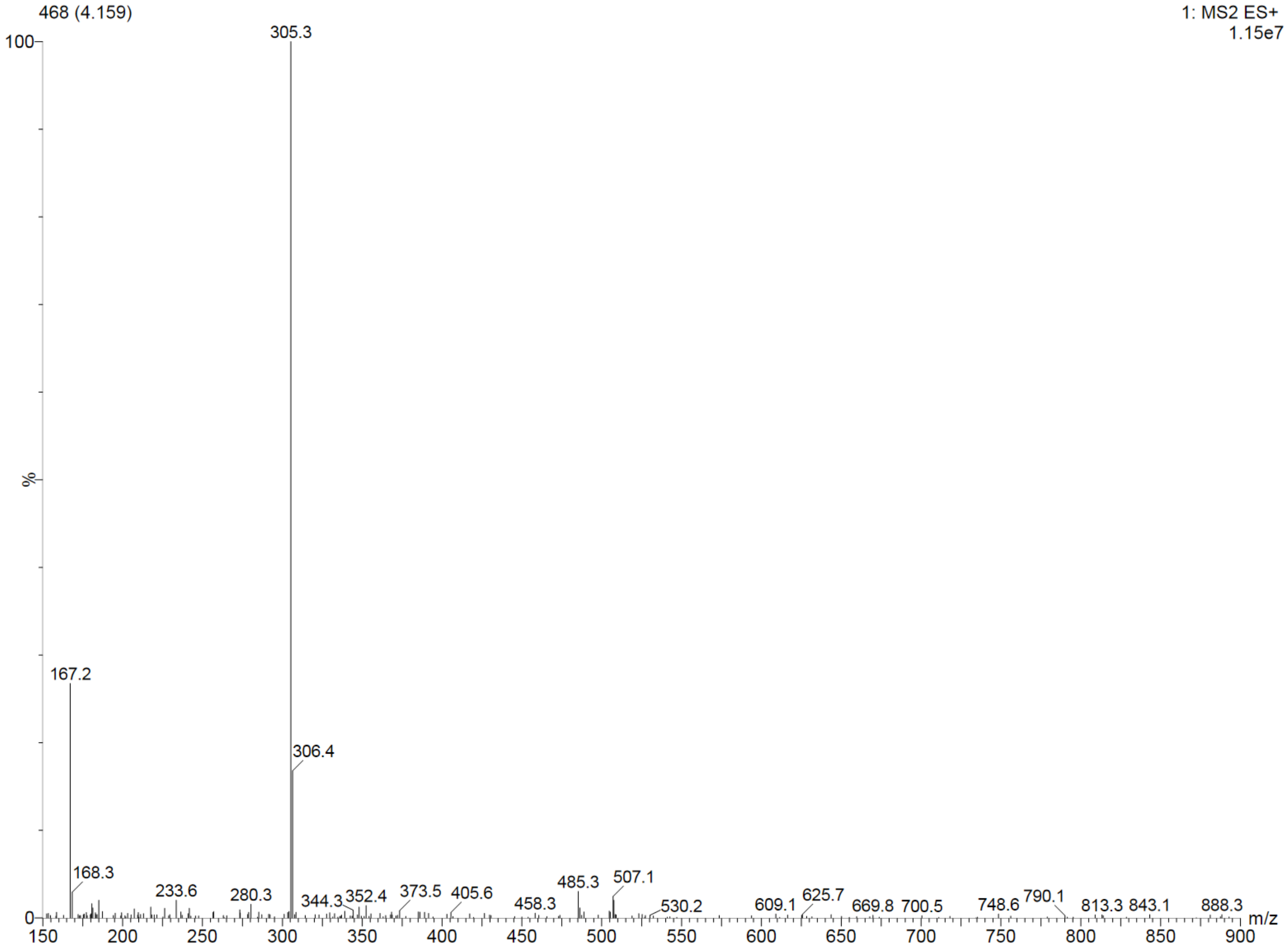
468 (4.159)
1: MS2 ES+
1.15e7
100
%
0
305.3
167.2
306.4
168.3
233.6
280.3
344.3
352.4
373.5
405.6
458.3
485.3
507.1
530.2
609.1
625.7
669.8
700.5
748.6
790.1
813.3
843.1
888.3
m/z
150
200
250
300
350
400
450
500
550
600
650
700
750
800
850
900
